# The long head of biceps at the shoulder: a scoping review

**DOI:** 10.1186/s12891-023-06346-5

**Published:** 2023-03-28

**Authors:** Brendan Diplock, Wayne Hing, Darryn Marks

**Affiliations:** Faculty of Health Sciences & Medicine, Bond University, Gold Coast, Australia

**Keywords:** Anatomy, Assessment, Function, Long head of biceps, Management, Scoping review, Shoulder

## Abstract

**Background:**

This review aimed to explore the available literature to update our understanding of the long head of biceps (LHB) at the shoulder. Synthesise our findings to identify emergent themes and knowledge gaps to inform future research and management directions.

**Methods:**

PubMed, Embase, Cinahl, SportDiscus, CENTRAL, and Web of Science were searched from inception to 31st December 2021. Articles were included if they referenced adult participants > 18 years of age and were written in English.

**Results:**

214 articles were included in the final analysis, and results were categorised into six emergent themes: (1) Anatomy - Normal anatomical variation of the biceps from aberrant origins, third and fourth accessory heads, and an absence of the LHB tendon (LHBT) are not necessarily benign, with shoulder pain and instability a commonly reported theme. (2) Function - Bicep’s role in glenohumeral elevation and stability in healthy shoulders is minimal. In contrast, LHB has a more significant role in shoulder stability and humeral head depression in subjects with rotator cuff failure or an absent LHBT. (3) Pathology - There is an association between LHB tendinopathy, rotator cuff disease, LHBT instability and occult rotator cuff tears. Early recruitment and hyperactivity of the LHB in subjects with symptomatic rotator cuff tears and instability suggest a potential compensatory role. (4) Assessment - The limited diagnostic utility of special orthopaedic tests in assessing LHBT pathology was a consistent theme. The utility of magnetic resonance imaging and ultrasound to identify full-thickness tendon tears and instability of the LHBT was moderate to high. However, the utility of clinical tests and imaging may be underestimated due to arthroscopy’s limitations in fully visualising the proximal LHBT. (5) Non-Surgical Management - Ultrasound-guided injections into the biceps sheath show greater accuracy and patient outcomes than blinded injections; however, the entry of injectate into the intraarticular glenohumeral joint may have unwanted complications. (6) Surgical management - For the surgical management of biceps pathology with or without rotator cuff pathology, both biceps tenodesis and tenotomy report similar improvements in pain without any significant adverse effect on strength or function. Tenodesis favoured higher overall constant scores and a lower incidence of Popeye deformity and cramping arm pain, with tenotomy trending to be more cost and time effective. For patients with a healthy LHBT, rotator cuff repair with adjunctive tenodesis or tenotomy fails to provide additional clinical improvements compared to rotator cuff repair in isolation.

**Conclusions:**

The scoping review highlights the variability of biceps anatomy, which is not necessarily benign and suggests a minimal role of the LHB in shoulder elevation and stability in healthy individuals. In contrast, individuals with rotator cuff tears experience proximal humeral migration and demonstrate hyperactivity of the LHB, suggesting a potential compensation role. The observed prevalence of LHBT pathology with rotator cuff tears is well established; however, the cause-and-effect relationship between LHBT pathology and rotator cuff disease is undetermined. The diagnostic utility of clinical tests and imaging to exclude LHBT pathology may be understated due to the limitations of arthroscopy to visualise the proximal LHBT fully. Rehabilitation programs for the LHB are understudied. Similar post-surgical clinical outcomes are observed for tenodesis and tenotomy for biceps and rotator cuff-related shoulder pain. Subjects undergoing biceps tenodesis are less likely to have cramping arm pain and a Popeye deformity than patients undergoing biceps tenotomy. The significance of routine surgical removal of the LHBT and sequelae on rotator cuff tear progression to failure and long-term shoulder function is unknown, and further research is required.

**Pre-registration:**

*OSF*: https://osf.io/erh9m

**Supplementary Information:**

The online version contains supplementary material available at 10.1186/s12891-023-06346-5.

## Background

The purpose of the Long Head of the Biceps (LHB) at the shoulder is an enigma. Its functional role at the shoulder and its contribution to glenohumeral joint arthrokinematics and stability remain poorly understood [1, 2]. Assessment of shoulder tendon pathology remains a clinical challenge due to the historically poor diagnostic utility of individual Orthopaedic Special Tests [3]. Generic management principles for shoulder pain typically include conservative treatment [1, 2, 4]. Often, specific physical therapy interventions and rehabilitation for the non-operative treatment of LHB-related shoulder pain are under-investigated.

In contrast, there is a plethora of literature on the surgical management (tenodesis vs. tenotomy) of LHB-related shoulder pain. Often surgical recommendations for routine LHB tenodesis/tenotomy with rotator cuff tears are dubious because the functional role of the LHB at the shoulder remains uncertain [5, 6]. Consequently, the impact of adjunctive LHBT surgery in rotator cuff-related shoulder pain on shoulder function remains a clinical quandary, with the longer-term implications for shoulder function and rotator cuff disease progression unknown.

Other fundamental questions about the LHB also remain unanswered. With the relationship between proprioception deficits at the shoulder and glenohumeral joint instability still unclear [7], perhaps the proprioceptive contribution of the LHB to the shoulder is desirable and worth retaining. Conversely, as previous reviews report normal anatomical variations of the LHBT, including a congenital absence [8], the LHBT may be considered vestigial, equivalent to the palmaris longus tendon of the wrist, implying its removal when pathognomonic lesions to be justified.

In summary, the function of the LHB at the shoulder remains poorly understood. Whilst previous narrative reviews [1, 2] provide a general overview of the LHB. A Preferred Reporting Items for Systematic Reviews and Meta-Analysis (PRISMA) research methodology and priori protocol in these historical reviews were lacking. They may represent an elevated risk of biased assessment [9, 10]. In addition to providing an update of the current literature, a scoping review protocol was chosen to; (a) explore the extent of available literature through a systematic PRISMA research methodology to decrease error and increase the reliability of assessment findings, (b) thoroughly investigate and synthesise the evidence into emergent themes, and (c) update our current understanding of the function of the LHB at the shoulder to inform future research and management directions. To our knowledge, this is the first scoping review on the LHB at the shoulder.

### Objective and research questions

The objectives of this review were: 1) review the research on (a) normal anatomy and function of the LHB at the shoulder, (b) abnormal pathology and assessment findings of the LHBT, (c) non-surgical and surgical management options for LHB-related shoulder pain and (d) functional impacts and disease sequelae of routine LHBT surgery in the operative management of rotator cuff related shoulder pain 2) synthesise findings, and 3) identify emergent themes and knowledge gaps for future research.

## Methods

### Protocol registration

A priori protocol was developed according to the Preferred Reporting Items for Systematic Reviews and Meta-Analysis Extension for Scoping Reviews (PRISMA-ScR): Checklist (Supplementary Table 1) and explanation [10]. This protocol was pre-registered with the Open Science Framework (OSF) https://osf.io/erh9m on September 12, 2021.

### Study design

The scoping review was guided by the methodology framework developed by Arksey and O’Malley [11], later refined by Levac, Colquhoun [12], Peters, Godfrey [13], Peters, Marnie [14] using a five-step search strategy of selected databases. (1) Identifying the research question; (2) Identifying relevant studies; (3) Selecting studies; (4) Charting the data, collating, and summarising; and (5) Reporting the results.

### Data sources and searches

The lead author ^(BD)^ undertook an initial pilot search of PubMed and Google scholar to identify articles on the topic and map key concepts. To develop a complete search strategy, synonyms were identified from text words in the titles and abstracts of relevant articles and the index terms used to describe the articles. A comprehensive search strategy, including all identified keywords and subject/index terms, was developed for PubMed and adapted using Polyglot [15] for all databases (PubMed MEDLINE, Embase, Cinahl, SportDiscus, CENTRAL, and Web of Science) by the lead investigator in consultation with a research university librarian (See Additional file 2). Following the search from inception to 31st December 2021, all citations were uploaded into EndNote 20.0.1 and duplicates were removed.

### Study selection

Titles and abstracts of eligible studies were screened against the inclusion criteria by the lead author ^(BD)^. Potential studies were retrieved in full text and assessed in detail against the eligibility criteria for inclusion in the scoping review. Full-text studies that did not meet inclusion criteria were excluded, and the reasons for exclusion were recorded and reported in the PRISMA Flow Diagram - Fig. 1 (See Additional file 3). All citations and references within selected studies were screened to identify additional studies for inclusion. The selection of studies was guided by the Participants-Concept-Context framework as recommended by the Joanna Briggs Institute (JBI) Methodological Guidelines for scoping reviews [14].

#### Participants

The study population was defined as adults (18 years or older) regardless of gender, a commonly used cut-off to distinguish the point between adolescence and adulthood.

#### Concept

Any published peer-reviewed literature reporting on the LHB’s role, function, or management at the shoulder and their outcomes.

#### Context and eligibility criteria

All full-text research articles produced in any year were eligible for inclusion. Exclusion criteria were defined as (1) non-English language, (2) no access to full text and (3) grey literature, including narrative literature reviews, thesis/dissertations, conference abstracts/proceedings, opinion pieces, magazine, and newspaper articles. Morrison, Polisena [16] found no evidence of a systematic bias from excluding languages other than English in a systematic review-based meta-analysis in conventional medicine.

### Quality assessment

Whilst the use of a critical appraisal tool was not required for this scoping review (Johanna Briggs Institute Reviewers’ Manual (2017) – Methodology for JBI scoping reviews [11, 12, 17]. The level of evidence for the 214 articles was independently assessed by the lead author ^(BD)^ using the JBI - levels of evidence guidelines and supporting documentation [18]. The level of evidence was verified by a blinded second assessor ^(HK)^, with eighteen discrepancies resolved with the assistance of a co-author ^(WH)^.

### Data extraction

Data from publications meeting eligibility criteria were extracted into customised tables. The data extraction tables were modified and revised iteratively during screening for each included study. Data charted in the final extraction tables included the author’s citation details, year of study, level of evidence and key study findings.

### Data analysis and synthesis

Studies were initially categorised according to three main domains (1) normal, (2) abnormal and (3) management. Key study characteristics and raw data were tabulated for each domain. Synthesis of key findings was performed to identify emergent themes and key concepts.

## Results

### Selection of sources of evidence

The search results are displayed in the PRISMA Flow Diagram - Fig. 1 (See Additional file 3). The search strategy generated 6031 results. Following duplicate removal and title and abstract screening, 425 full-text articles were reviewed for inclusion in the study and sixty-two additional records were identified through reference list searches totalling 487 full-text articles. Of these, 273 were excluded, and 214 articles were included in this scoping review.

### Characteristics of sources of evidence

The years of publication for included studies ranged from 1948 to 2021, with 2014 to 2021 producing the most publications (Table 1). Studies were categorised into emergent themes; (a) anatomy, (b) function, d) pathology, e) assessment and f) management. Emergent themes were further dichotomised into sub-themes and by the level of evidence (Table 2).

## Anatomy

The normal origin of the Long Head of the Biceps (LHB) has traditionally been from the supraglenoid tubercle and superior labrum, with its proximal tendon tracking distally through the rotator cuff interval where the biceps pulley envelops it and houses within the biceps groove. Upon exiting the biceps pulley, it travels under the transverse humeral ligament, where it joins the short head of the biceps to form the biceps brachii muscle belly, sharing a common distal tendon attachment onto the radial tuberosity ligament [19]. Thirty-six articles in Supplementary Table 2 include normal anatomical variations of the biceps brachii and the impact of aberrant origins, supernumerary accessory heads and a congenital absence of the LHBT on shoulder pain and function. Supplementary Table 3 includes three studies on the normal anatomy of the transverse humeral ligament.

**Table 1:** Characteristics of included studies by year

Year	1940–1969 (n = 1)	1970–1999 (n = 25)	2000–2004 (n = 15)	2005–2009 (n = 26)	2010–2014 (n = 40)	2015–2019 (n = 67)	2020–2022 (n = 40)
Reference number(s)	[20]	[21–45]	[46–60]	[8, 61–85]	[86–125]	[19, 126–191]	[192–231]

**Table 2:** Characteristics of included studies by themes and level of evidence

Themes	Level of evidence (LOE)				
Anatomy (n = 39)	Level 1 (n = 0)	Level 2 (n = 1)	Level 3 (n = 1)	Level 4 (n = 27)	Level 5 (n = 10)
Reference number(s)	-	[57]	[163]	[8, 34, 39, 54, 56, 58, 63, 68, 74, 79, 82, 105, 109, 123, 125, 129, 141, 144, 167, 171, 173, 184, 185, 189, 207, 209, 212]	[28, 33, 36, 69, 76, 80, 86, 113, 138, 219]
Function (n = 47)	Level 1 (n = 1)	Level 2 (n = 3)	Level 3 (n = 16)	Level 4 (n = 14)	Level 5 (n = 13)
Reference number(s)	[193]	[99, 120, 130]	[20, 25, 37, 42, 47, 51, 65, 83, 102, 103, 114, 116, 121, 190, 213, 228]	[21, 23, 29, 40, 44, 46, 52, 70, 78, 84, 101, 112, 117, 159]	[27, 30–32, 35, 38, 50, 85, 88, 94, 100, 106, 153]
Pathology (n = 37)	Level 1 (n = 2)	Level 2 (n = 4)	Level 3 (n = 18)	Level 4 (n = 10)	Level 5 (n = 3)
Reference number(s)	[178, 196]	[48, 89, 158, 198]	[53, 55, 61, 62, 87, 98, 148, 152, 157, 161, 177, 179, 191, 197, 203, 205, 217, 227]	[22, 45, 59, 75, 77, 111, 124, 137, 172, 195]	[24, 64, 96]
Assessment (n = 50)	Level 1 (n = 10)	Level 2 (n = 20)	Level 3 (n = 8)	Level 4 (n = 9)	Level 5 (n = 3)
Reference number(s)	[43, 95, 107, 108, 118, 128, 150, 183, 188, 221]	[41, 60, 67, 73, 81, 90, 104, 110, 115, 132, 139, 149, 165, 166, 168, 169, 175, 181, 187, 214]	[122, 134, 155, 156, 160, 162, 199, 206]	[49, 66, 72, 135, 140, 144, 164, 176, 180]	[119, 151, 220]
Management (n = 41)	Level 1 (n = 7)	Level 2 (n = 7)	Level 3 (n = 18)	Level 4 (n = 7)	Level 5 (n = 2)
Reference number(s)	[97, 131, 142, 201, 215, 230, 231]	[91, 114, 126, 182, 210, 226, 229]	[19, 26, 71, 92, 93, 136, 146, 154, 170, 174, 192, 194, 200, 204, 208, 216, 222, 223]	[133, 143, 145, 147, 202, 218, 224]	[186, 225]
Totals (n = 214)	Level 1 (n = 20)	Level 2 (n = 35)	Level 3 (n = 61)	Level 4 (n = 67)	Level 5 (n = 31)

### Anatomical variation

Of the thirty-nine articles on biceps brachii anatomy found, thirty-six papers reported anatomical variations from normal. Supplementary Table 2 details the reported variants by type and number. The most common observed anatomical variations pooled across all 36 studies included a) aberrant origins (n = 136), supernumerary accessory heads (n = 98) and LHBT absence (n = 48). The most common aberrant origin of the LHB at the shoulder was the presence of a bifurcate tendon origin in seven articles and an anomalous LHBT origin from the supraspinatus in six articles. A trifurcate origin of the LHBT was seen in three studies, with a solitary case of Chondroepitrochlearis and an LHBT traversing through a bifid Subscapularis found in one paper. Nine studies reported the presence of a third supernumerary head in forty-nine shoulders, and two reported a four-headed biceps variant in nine shoulders. The overall reported prevalence of anatomical variations of the biceps brachii in the case study series ranged from 2.2 to 70% [28, 34, 36, 57, 58, 86, 113, 163], as detailed in Supplementary Table 2. Three studies reported no distinct anatomical structure of the transverse humeral ligament to support a role in LHBT stability in the bicipital groove, as detailed in Supplementary Table 3.

### Impact of variation

Of the twenty individual case studies in Supplementary Tables 2, fourteen reported shoulder pain associated with the anatomical variation, an association between pain and aberrant origins in nine studies and pain and LHBT absence in five studies. In four case studies, three articles reported shoulder instability associated with an absence of the LHBT and shoulder instability and weakness in one case study. Shoulder instability and pain were reported in five articles, associated with aberrant origins in two case studies and LHBT absence in three case studies. Two articles observed a single aberrant origin and absent LHBT associated with shoulder pain and restriction of movement in two case studies. A solitary case study observed a Partial Articular-sided Supraspinatus Tendon Avulsion (PASTA) in two patients with an anomalous origin of the LHBT from the Supraspinatus. Two large case series studies and a single systematic review reported a positive correlation between (a) observed anatomical variances of the LHBT origin and the presence of anterosuperior labral fraying (odds ratio, 3.58; p = 0.000), abnormal superior glenohumeral ligament (odds ratio, 3.69; p = 0.012) [57], and (b) LHBT absence and the presence of shoulder pain (85.7%) and instability (37.1%) [167]. Whilst shoulder joint instability was more prevalent in biceps variations in the mesotenon; it did not reach significance (27.6%: 14.9%, p = 0.305) [163].

## Function

In this section of LHB function at the shoulder, forty-seven articles were categorised under the subthemes of (a) biceps electromyography, (b) shoulder biomechanics (Supplementary Table 4), (c) shoulder stability in vivo (Supplementary Table 5), (d) glenohumeral joint arthrokinematics in vitro (Supplementary Table 6), and (e) shoulder proprioception as detailed in Supplementary Table 7.

### Electromyography (EMG)

Twenty-seven studies used electromyography (EMG) to investigate the activity of the biceps brachii, as detailed in Supplementary Table 4. Thirteen studies differentiated long head vs. short head of biceps activity. Sixteen studies used surface electromyography (sEMG) to record biceps brachii activity during functional tasks of the shoulder [21, 29, 42, 44, 70, 83, 84, 103, 112, 114, 116, 117, 121, 159, 190, 213]. Four studies used fine wire electromyography (fwEMG) to record biceps brachii muscle activity [20, 23, 25, 51]. Seven studies used a combination of both sEMG and fwEMG for recording activity of the biceps brachii and other shoulder muscles, such as the rotator cuff and deltoid muscles [40, 46, 52, 101, 102, 120, 228].

### Biomechanics

Thirteen articles detailed in Supplementary Table 4 investigated the role of the LHB in healthy shoulders. Six studies demonstrated LHB muscle activity during shoulder elevation [20, 21, 44, 70, 103, 116]. Three studies validated LHB activity during overhead throwing and bowling [23, 84, 114]. In contrast, four studies supported no significant LHB activity during shoulder elevation [40, 52, 101, 121]. Studies using an elbow brace or elbow flexor fatigue protocol to minimise biceps function at the elbow confirmed an active role of the LHB during shoulder elevation in two studies [44, 116].

In contrast, three studies found no significant or minimal activity of the LHB during shoulder elevation [40, 52, 121]. Two studies of biceps brachii activity during common therapeutic shoulder exercises demonstrated the highest biceps activity in the sagittal plane, during unsupported elbow flexion and supination, from an extended position (e.g., shoulder extension) or during high-velocity explosive exercises such as underarm throwing [117, 159]. Two studies reported more significant activity and strength production of biceps brachii during biceps curl variations in shoulder elevation [83, 112]. One study reported more significant biceps brachii activity during isometric shoulder external rotation in sitting than supine. One study supported a pre-activation and a pre-setting role of the LHB and rotator cuff during shoulder rotation at 45° scaption in normal asymptomatic subjects [46].

### Shoulder stability

Six in vivo studies examined the role of the biceps brachii in shoulder stability, as detailed in Supplementary Table 5. Five studies support a role in glenohumeral joint stability and humeral head depression [37, 47, 56, 65, 78] and a single study reported a minor role in shoulder stability [99]. Active humeral head depression at the shoulder by the biceps brachii in patients with rotator cuff lesions was observed under radiology assessment in one study [47]. A large study by Walch, Edwards [65] of 291 patients undergoing LHB tenotomy for irreparable full thickness tears of the rotator cuff demonstrated a significant superior migration of the head of the humerus following tenotomy. A single case report of incidental arthroscopy findings of LHBT absence reported an association with shoulder instability which improved after thermal capsulorraphy [56]. A study of seven patients with an LHBT absence under arthroscopy/MRI demonstrated superior glenohumeral translation in shoulders compared to intact shoulders [37]. One study of thirty asymptomatic volunteers showed a dynamic role of the LHB in glenohumeral joint stability during low angles of shoulder elevation < 30° [78]. In contrast, a study of five patients undergoing biceps tenodesis for chronic biceps tenosynovitis demonstrated no significant alterations in glenohumeral joint position or activity after LHB tenotomy compared to healthy shoulders [99].

### Glenohumeral joint arthrokinematics

Thirteen in vitro cadaver studies detailed in Supplementary Table 6 examined the role of the LHB in intact shoulders and six articles with simulated shoulder injuries, including rotator cuff tears, Bankart and superior labral lesions [30–32, 35, 88, 94]. Ten studies demonstrated a shoulder stability role for the LHB [30–32, 35, 38, 85, 88, 94, 106, 153]. Six studies demonstrated a humeral head depressor role [27, 38, 50, 88, 106, 153]. One study demonstrated a glenohumeral joint compressor role [100]. The observed and often stated limitations of the cadaver studies found included rationales to determine appropriate biceps forces in vitro that accurately represent the normal physiological loads of the LHB in vivo. Three studies used a biceps load of 55-N force, predicted from the Physiological Cross-Sectional Area (PCSA) of the biceps brachii [35, 38, 88]. Two studies used progressive biceps loads of 10, 20 and 40-N based on previously published PCSA values [100, 153]. A single study used %PCSA (55-N) to determine a passive 5 N (10%) and active 25 N (50%) biceps preload [94]. A single study used PCSA to determine physiological biceps loads with no reported values stated [50]. Two studies used a biceps load of 1.5 and 3 kg [30] and 1.5 kg [31], proportional to PCSA. Two studies used a 3 kg [27] and a 20-N [106] simulated biceps load with no reported rationale. Two studies calculated physiological biceps loads using a combination of PCSA and EMG percentage of a maximal voluntary contraction during a functional task, e.g., throwing [32, 85].

### Shoulder proprioception

No direct evidence for or against the role of the LHB in proprioception at the shoulder was found. Two systematic reviews on proprioception and shoulder pain/pathology were found. Ager, Borms [193] reported moderate evidence for an affected sense of kinaesthesia and insufficient evidence for an impaired sense of force amongst painful shoulders. Fyhr, Gustavsson [130] reported deficits in movement sense and passive joint reposition sense following post-traumatic shoulder instability compared to the contralateral uninjured shoulders and controls, as detailed in Supplementary Table 7.

## Pathology

Thirty-five diagnostic studies in Supplementary Tables 8 and eight EMG studies, as detailed in Supplementary Tables 9, were categorised under the sub-themes of LHBT pathology, rotator cuff pathology and compensation.

### Long head of biceps pathology

As detailed in Supplementary Tables 8, common LHBT pathologies include tendon instability, tendinopathy (ruptures, tears, lesions, tendinitis, sheath effusions), bicep pulley lesions, bicipital groove dysplasia, labral variants/SLAP lesions and an absence of LHBT. Observed biceps pathology associated with the LHB’s anatomical variations involved anterosuperior labrum variants, a Buford complex, SLAP lesions, and biceps instability [53, 77, 205, 227]. Other pathological associations included; (a) LHBT pulley lesions and degenerative LHB tendinopathy with biceps instability and degenerative glenohumeral joint disease [111], (b) LHB tendinitis and pro-inflammatory mediators, neovascularisation, and gene expression [191], (c) absent LHBT and shoulder pain [179], (d) LHBT rupture, instability with shoulder pain and permanent elbow/forearm strength and endurance loss [62], and LHBT tears with occult distal biceps lesions extending beyond the biceps groove (Supplementary Table 15) [135].

### Rotator cuff pathology

Concomitant rotator cuff pathology associated with LHB tendinopathy includes rotator cuff tears, Supraspinatus and Subscapularis tears, Supraspinatus tendinopathy and rotator cuff tendinopathy. A high association was observed between LHBT instability and rotator cuff pathology in twelve articles, inclusive of Subscapularis tears in eight articles [61, 98, 137, 152, 178, 195, 196, 203], isolated Supraspinatus tears and Subscapularis tears in two studies [24, 227] and rotator cuff tears in two studies [45, 75]. A lower association was observed between the incidence of biceps pulley lesions and rotator cuff tears, SLAP lesions, anterosuperior impingement and increasing age [89]. Absence of the LHBT with rotator cuff tears [217]. Biceps groove morphology with LHBT instability and Subscapularis tendinopathy [157, 158]. LHBT sheath effusion and rotator cuff tears [148] and increased cross-sectional area of LHBT with rotator cuff tears [64] as detailed in Supplementary Table 8.

### Compensation

Eight EMG studies were found directly supporting increased muscle activation of the biceps brachii in the presence of rotator cuff pathology and shoulder instability, as detailed in Supplementary Table 9. Two studies demonstrated significant hyperactivity of the biceps brachii compared to control groups in the presence of rotator cuff tears during reaching and shoulder elevation tasks [42, 102]. A single study reported a significant delay in anticipatory muscle activation in subjects with rotator cuff tears during a ball drop task [213]. A recent study demonstrated both hyperactivity and pre-activation of the biceps brachii in patients with rotator cuff tears during reaching tests [228]. In support of biceps activity during throwing, two studies reported significant hyperactivity of the biceps brachii during simulated pitching and cocking phases of throwing in subjects with anterior shoulder instability vs. controls [25, 51]. In contrast to the observed changes in muscle activity with rotator cuff pathology, two studies reported no significant differences in (a) biceps brachii activity with the elbow immobilised in a brace during shoulder range of motion in patients with rotator cuff tears vs. controls [40] and (b) in activity or fatigue of the biceps brachii during a gripping task in patients with rotator cuff tears [120].

## Assessment

Assessing the LHBT in the clinical examination can be completed in several ways. Fifty articles investigating the assessment of the LHBT pathology at the shoulder were categorised into several sub-themes, including (a) screening questionnaires and (b) clinical examination (Supplementary Tables 10, 11 and 12), (c) diagnostic imaging such as magnetic resonance imaging/arthrography (Supplementary Table 13) and ultrasound, (d) diagnostic injections (Supplementary Table 14) and (e) arthroscopy (Supplementary Table 15).

### Screening questionnaires

As detailed in Supplementary Tables 10, a single article reported limited validity for using postoperative screening questionnaires to assess pre-operative LHBT pathology [149].

### Clinical examination

As detailed in Supplementary Tables 10, a single study established a clinical examination algorithm for the standardisation of shoulder assessment, including the differential diagnosis of biceps pathology [220]. Three studies examined the clinical utility of a symptom modification approach to diagnosing and managing shoulder musculoskeletal conditions, including Rotator cuff and LHB-related shoulder pain. Of two studies on the Shoulder Symptom Modification Procedure (SSMP), one [151] found high inter-rater reliability. Yet, the authors acknowledged that the efficacy of the Shoulder Symptom Modification Procedure (SSMP) in treating shoulder pain was yet to be established. The other more recent study [175] reported moderate inter-rater reliability but found the association between within-session and between-session changes in pain regarding treatment efficacy for the Shoulder Symptom Modification Procedure (SSMP) to be insufficient. The authors consequently concluded that the Shoulder Symptom Modification Procedure (SSMP) was not reliable or valid for managing shoulder pain. A single study of a symptom modification test cluster for shoulder instability reported a significant, meaningful relationship between GIRD - glenohumeral internal rotation deficit and an impingement GIRD apprehension-relocation test cluster [206].

Several systematic reviews and diagnostic studies examined the validity of traditional and contemporary Orthopaedic special tests for LHBT pathology with considerable variability found, whilst two studies showed a limited diagnostic yield of bicipital groove tenderness for LHB tendinopathy [73, 169], as detailed in Supplementary Table 11. Three high levels of evidence studies supported the use of test clusters to improve their diagnostic yield (biceps groove palpation, Speed’s, Yergason’s and Uppercut test) with higher diagnostic utility demonstrated when performed in combination with diagnostic ultrasound (Supplementary Table 12) [81, 169, 183].

### Magnetic resonance imaging and arthrography

Eighteen studies were found investigating the validity of Magnetic Resonance Imaging (MRI) and Arthrography (MRA), compared with arthroscopic surgery in seventeen of the eighteen studies found diagnosing proximal LHBT pathology at the shoulder, exclusive of SLAP lesions and labral pathology as detailed in Supplementary Table 13. Overall diagnostic accuracy, specificity, and sensitivity values for MRA were superior to MRI for LHBT pathology, though, the difference was negligible. Both diagnostic utilities varied considerably for LHB tendinopathy, including tears, tendinitis, tendinosis, instability, and pulley lesions. There was an overall trend for low sensitivity and high diagnostic accuracy and specificity values for the exclusion and detection of full thickness and complete tears of the LHBT for both MRI and MRA, respectively, compared with low specificity and sensitivity values to rule in and out partial thickness tears of the LHBT [60, 90, 118, 139, 150, 155, 180, 188]. LHBT instability demonstrated variable specificity, and sensitivity values with a trend for higher specificity values to rule in dislocation of the LHBT compared with subluxation, whilst the sensitivity values in many of the studies identifying LHBT instability were poor [60, 90, 118, 134, 139, 150, 155, 165, 180, 188]. In a single systematic comparison of MRI and MRA, MRA demonstrated higher specificity for detecting LHBT appearance and position than MRI [221]. A single study of biceps pulley lesions on MRA compared with arthroscopy reported high accuracy, sensitivity, and specificity values for MRA [104]. Contrary to clinical intuition, a single study of the impact of Body Mass Index (BMI) on MRI accuracy demonstrated significantly higher diagnostic accuracy of MRI for the detection of Bankart lesions in both overweight and obese patients when compared with patients with a healthy Body Mass Index (BMI) (Supplementary Table 10) [214].

### Diagnostic ultrasound

Ten studies examined the validity of diagnostic ultrasound in detecting LHBT pathology compared with arthroscopy in four studies, arthroscopy/open surgery in two, arthroscopy/arthrotomy in one and MRI in two studies, as detailed in Supplementary Table 14. Two articles reported high specificity and sensitivity values for ruling in and out full-thickness tears. In comparison, there was an observed trend for lower sensitivity values to rule out partial thickness tears of the LHBT [67, 95]. Two studies demonstrated variable specificity and sensitivity for complete tears [128, 181]. Two studies showed high specificity for LHBT rupture [49, 181]. Biceps instability demonstrated an overall trend for increased specificity and sensitivity values for ruling in and out dislocation > subluxation of the LHBT [43, 49, 67, 128, 181]. The specificity of diagnostic ultrasound to rule in tendinitis was high, and its sensitivity to rule out tendinitis was low [43, 110]. A single cross-sectional study comparing the diagnostic accuracy of ultrasound vs. MRI in detecting LHBT pathology to arthroscopy showed a trend for higher specificity for MRI compared to diagnostic ultrasound for identifying proximal LHBT pathology. Sensitivity values for both remained poor [162].

### Diagnostic injection

A single study on diagnostic injections for LHBT pathology reported high specificity but low sensitivity to exclude LHBT pathology [169]. A cadaver study by Gofeld, Hurdle [186] demonstrated the spread of injectate from the biceps sheath into the glenohumeral joint and cartilage in 92% of specimens suggesting the limited diagnostic utility of injections for LHB pathology as detailed in Supplementary Table 14.

### Arthroscopic surgery

Supplementary Table 15 detailed four clinical studies that presented findings of LHBT pathology (lipstick sign, sentinel sign, chondral print, and the ramp test). One study reported the presence of a detour sign on MRI as a useful clinical indicator of LHBT subluxation, occult Subscapularis, and Supraspinatus rotator cuff pathology [166]. Arthroscopic surgery as a standard gold reference for confirmation of LHBT pathology at the shoulder was used in most diagnostic and imaging studies, as detailed in Supplementary Tables 11, 12, 13, and 14. In Supplementary Tables 15, the limitations of arthroscopy to fully evaluate the proximal intra-articular LHBT-labral complex and identify occult extra-articular bicipital tunnel lesions were reported in four articles [119, 132, 140, 164].

## Management

In this section of Management, forty-one articles were categorised under non-surgical (Supplementary Tables 16 and 17) and surgical management. Surgical management was further dichotomised into operative intervention for (a) LHBT-related shoulder pathology (Supplementary Table 18), (b) rotator cuff-related shoulder pathology (Supplementary Table 19) and (c) rotator cuff and LHBT-related shoulder pathology, as detailed in Supplementary Table 20.

### Non-surgical management

Supplementary Table 16 details our limited findings of four articles reporting on the non-surgical management of LHB-related shoulder pain. A single paper presented a care pathway for shoulder pain management, including LHBT management in primary care [220]. Two EMG studies demonstrated a progression of biceps-targeted activity from least active to most active during common shoulder rehabilitation exercises in healthy subjects [117, 159]. A single study by McDevitt, Snodgrass [202] demonstrated support for including dry needling, eccentric, concentric exercise, and biceps brachii stretching for managing chronic LHB tendinopathy.

Seven studies investigated the injection of dye, local anaesthetic, and corticosteroid into the LHBT sheath (Supplementary Table 17). None compared injection with no injection or placebo. Six reported greater accuracy and pain relief when injections were guided by ultrasound or fluoroscopy than unguided injections [91, 93, 97, 126, 210, 232]. One cadaver study found a high rate of dye migration into the glenohumeral joint capsule and the potential for wanted or unwanted deposition of injectate into the intra-articular glenohumeral joint [186]. One article compared non-surgical care versus tenodesis for LHBT ruptures. Patients managed non-surgically tend to return to work quicker but at lesser capacity and with less elbow flexion and forearm supination strength than patients who underwent biceps tenodesis [26]. One systematic review reported common surgical indications for tenodesis, including LHBT tears, instability, and tenosynovitis [145].

### Surgical management

In total, thirty-five articles compared postoperative outcomes in patients undergoing LHB tenodesis versus tenotomy, with tenodesis the most common procedure reported (n = 9983) vs. tenotomy (n = 6855). Twenty-two articles examined LHB tenodesis and tenotomy outcomes in subjects with LHBT-related shoulder pathology in isolation (Supplementary Table 18). Six articles investigated the effects of adjunctive LHBT surgery in subjects with rotator cuff-related shoulder pathology (Supplementary Table 19), and seven articles reported tenodesis and tenotomy outcomes in subjects with concomitant rotator cuff and LHBT-related shoulder pathology, as detailed in Supplementary Table 20.

#### Surgery for LHBT pathology

For subjects undergoing tenodesis vs. tenotomy for LHBT-related shoulder pathology, the overall postoperative Patient Reported Outcome Measures (PROMS) were similar for both tenodesis and tenotomy, across the twenty-two articles found, with a minor trend for improved constant scores in favour of tenodesis in three articles [131, 194, 224]. A higher prevalence of Popeye deformity in fourteen articles and cramping arm pain in six were observed in patients who undertook tenotomy vs. tenodesis, as detailed in Supplementary Table 18. Decreased post-operative narcotic use and quicker pain relief was observed in one article for patients following biceps tenotomy [182], with longer surgical times observed in patients post-tenodesis [131]. Three studies that dichotomised their results by patient demographics showed a greater incidence of Popeye deformity in male patients [92], with less satisfaction in younger male patients following tenotomy compared with tenodesis [229]. Male gender, younger age, and active worker’s compensation were associated with increased complications following tenotomy [204]. For articles comparing LHB tenodesis vs. tenotomy for LHBT or SLAP pathology, similar trends were observed, with no significant differences in postoperative PROMS for pain, function, and strength [197, 215, 216]. However, a more significant trend was observed for increased shoulder flexion range of motion (ROM) and less postoperative stiffness in patients who undertook open compared with arthroscopic tenodesis [216]. One article by Horan, Dekker [197] comparing SLAP repair vs. biceps tenodesis in young overhead-throwing athletes demonstrated PROMS with a high rate of return to overhead-throwing sports. Similar strength scores were reported across all studies, apart from two articles reporting subjective biceps weakness at the elbow following tenotomy [92, 204]. A comparison of arthroscopic vs. open tenodesis reported greater postoperative shoulder flexion for patients who underwent open tenodesis [146]. Post-surgical follow-up in the twenty-two articles was highly variable, ranging from 3 months to 13 years, with many studies reporting a mean follow-up time ≥ two years. Result findings are medium-term and likely to represent post-surgical outcomes over time.

#### Adjunctive surgery on a healthy LHBT for rotator cuff pathology

Of the six articles reporting outcomes of adjunctive biceps surgery for repairable and irreparable rotator cuff tears, similar levels of patient satisfaction and PROMS were observed across all studies, as detailed in Supplementary Table 19. Two articles comparing post-operative clinical outcomes of patients undertaking either tenodesis or tenotomy for irreparable rotator cuff tears without rotator cuff repair reported high levels of patient satisfaction and PROMS [65, 71]. Of the four articles investigating adjunctive tenodesis or tenotomy with rotator cuff repair, higher surgical costs were reported in patients who underwent (a) rotator cuff repair with adjunctive tenodesis compared with adjunctive tenotomy or rotator cuff repair in isolation and (b) arthroscopic tenodesis compared with open biceps surgery [218, 222]. There was a trend for a higher incidence of Popeye deformity in three articles [71, 133, 154] and cramping arm pain in one article [154] for patients who underwent tenotomy. An article by Meraner, Sternberg [154] reported greater postoperative shoulder abduction strength in patients who underwent rotator cuff repair with adjunctive tenodesis compared with tenotomy. In addition to the six articles discussed, one clinical trial by Godenèche, Kempf [174], Supplementary Table 20 compared clinical outcomes following tenodesis or tenotomy in subjects with Supraspinatus rotator cuff tears and no LHBT pathology (normal) vs. Supraspinatus rotator cuff tears with LHBT pathology over a 10-year follow-up period. For patients with a healthy LHBT, similar constant scores were reported between Supraspinatus repair in isolation and Supraspinatus repair with either adjunctive tenodesis or tenotomy. The authors concluded that adjunctive LHBT surgery with Supraspinatus repairs should be avoided intraoperatively in patients with a normal LHBT. In the seven articles, post-surgical follow-up times ranged from 12 months to 10 years. Except for one 10-year study, most studies reported a mean follow-up time of ≤ 3 years. Result findings were, therefore, medium to long-term and likely represented stable post-surgical outcomes over time.

#### Surgery for rotator cuff and LHBT pathology

As detailed in Supplementary Tables 20, seven articles reported postoperative outcomes in subjects with concomitant rotator cuff and LHBT-related shoulder pathology. Five articles reported similar post-operative clinical results in subjects who undertook routine rotator cuff repair and adjunctive tenodesis or tenotomy, with a trend for higher constant scores in patients who undertook tenodesis [19, 142, 147, 170, 225]. Again, there was a trend for a higher incidence of Popeye deformity in three articles [147, 170, 225] and cramping arm pain in two papers [147, 225] in patients who underwent tenotomy with similar rotator cuff repair rates observed. Shorter operative times were observed in patients who underwent rotator cuff repair in isolation compared with patients who underwent rotator cuff repair and adjunctive tenodesis [142]. For subjects with a concomitant Supraspinatus tear and confirmed intraoperative LHBT pathology, Godenèche, Kempf [174] reported higher constant scores for function and strength in patients who underwent Supraspinatus repair with either adjunctive LHB tenodesis or tenotomy, compared with patients who underwent isolated Supraspinatus repair. Similar higher PROMS in subjects undergoing rotator cuff repair and adjunctive tenodesis or tenotomy compared with rotator cuff repair in isolation were observed in another article [19]. A study of patients with concomitant rotator cuff tears and SLAP lesions reported similar clinical outcomes in PROMS and shoulder ROM for patients who undertook rotator cuff repair with SLAP repair vs. subjects who undertook rotator cuff repair with adjunctive tenodesis [200]. Post-surgical follow-ups ranged from 1 month to 10 years, with most studies reporting a mean follow-up time ≤ to 4 years. Pooled result findings are medium-term and highly likely to represent post-surgical outcomes over time.

## Discussion

### Anatomy

Our scoping review observed considerable anatomical variation of the LHB from double or triple tendon aberrant origins and supernumerary third and fourth accessory heads to a congenital absence of the LHBT. These observed variations were associated with higher shoulder pain and instability prevalence. Morris, Guettler [233] and Kanatli, Ozturk [234] reported similar observations, concluding that anatomical variations of the LHB are not always benign. The role of the transverse humeral ligament in LHBT stability was unclear, with the literature invalidating the transverse humeral ligament as a distinct anatomical structure. The growing consensus in the literature supports the biceps pulley as the primary stabiliser of the LHBT in the bicipital groove [235].

### Function

The role of the biceps brachii at the elbow joint and its contribution to elbow flexion and forearm supination is well documented and understood in the literature [19]. Our scoping review found a lack of consensus for an active role of LHB at the shoulder during shoulder elevation and throwing activities, with a minor role in shoulder stability in normal healthy shoulders. In contrast, we found an established humeral head depressor function at the shoulder in (a) subjects with rotator cuff tears and shoulder instability, (b) in patients following adjunctive LHB tenotomy for irreparable rotator cuff tears, (c) in patients with an absent LHBT and (d) during physiological loading of the biceps brachii in cadaver shoulders with simulated rotator cuff and labral lesions. The role of the LHB in proprioception at the shoulder was underdetermined. Our scoping review found that both sEMG and fwEMG can be used effectively to investigate biceps brachii and LHB activity. Chalmers, Cip [116] established that both the long and short heads of the biceps brachii have separate muscular fibres and can be studied effectively through sEMG. Marri and Swaminathan [236] confirmed the usefulness of sEMG analysis of the biceps brachii in training and rehabilitation programs.

### Pathology

There was an established association between LHB tendinopathy and rotator cuff pathology and LHBT instability and occult Subscapularis tears in our scoping review, with EMG studies demonstrating hyperactivity of LHB in the presence of rotator cuff tears and shoulder instability, suggesting a compensation role of the LHB in pathological shoulders. Similar changes in muscle activation of the deep abdominal muscles have been established in people with chronic low back pain [237–239]. Our conclusions agree with Khandare, Arce [240] and Kaar [241] that further research is required to determine if the LHB muscle compensates for rotator cuff pathology and shoulder instability. We hypothesise that the increased biceps activity observed in patients with rotator cuff deficiency may explain the high prevalence of concomitant LHBT pathology with rotator cuff disease severity as it attempts to compensate for rotator cuff deficiency at the shoulder. Occult Subscapularis tears typically occur when biceps sling integrity is compromised, displacing the unstable LHBT anteromedially into the Subscapularis tendon, leading to subsequent tearing [242–244]. Other potential mechanisms of LHB tendinopathy include mechanical constriction and irritation from a bottleneck narrowing of the biceps groove [245].

### Assessment

The limited diagnostic utility of screening questionnaires, special orthopaedic tests, and palpation in the differential diagnosis of LHBT pathology was a consistent theme in our scoping review. Due to the variable diagnostic utility of special orthopaedic tests of the shoulder [3, 246–255], it was not surprising that the diagnostic accuracy of special orthopaedic tests to identify LHBT pathology was also highly variable, with the use of test clusters improving test accuracy. The use of a symptom modification approach during the physical examination of the shoulder is contemporary [256]. Our study findings agree with the conclusions drawn by Lehman [257] that the definitive mechanisms underpinning the symptom modification approach are yet to be fully understood, and more research is required to validate its use in shoulder pain. Proximal LHBT tenderness at the bicipital groove seems to have little utility for diagnosing LHBT pathology.

The American College of Radiology Appropriateness Criteria for Atraumatic Shoulder Pain recommends MRI or diagnostic ultrasound be performed when LHBT pathology is suspected, and initial radiographs are normal or inconclusive. Our scoping review findings support using MRA over MRI to diagnose LHBT appearance, position, and biceps pulley lesions for LHBT pathology. Most MRI and MRA studies reported a high utility for detecting full thickness and complete tears of the LHBT; however, the utility for MR imaging to detect and exclude partial thickness tears of the LHBT was low. Overall MR imaging to detect LHBT instability was found to be variable; though, there was an observed trend for higher specificity to rule in LHBT dislocation compared with subluxation. MRI and MRA sensitivity values were lower than the reported specificity values.

Similar to our MR findings, several studies reported the higher diagnostic utility of ultrasound for biceps dislocation greater than subluxation and the detection of full-thickness tears of the LHBT. However, its ability to identify partial-thickness tears of the LHBT was less accurate. Similar findings were found for diagnosing biceps tendinopathy. Study comparisons between diagnostic ultrasound and MRI reported high specificity values for MRI greater than diagnostic ultrasound for proximal LHBT pathology, but sensitivity values for both were overall poor. A recent literature review by Ostrowski, Beaumont [258] found similar results with a reported high accuracy of musculoskeletal ultrasound to diagnose LHBT dislocation, subluxation, and full-thickness tears, and less accuracy in diagnosing partial-thickness tears and tendonitis. Arthroscopy was the gold reference standard in most diagnostic tests and imaging studies. The reported limitations of arthroscopy to fully visualise the proximal LHBT at the glenohumeral joint in our scoping review findings may underestimate their reported accuracy and sensitivity values, and more research is required to validate their use in the diagnosis of LHB-related shoulder pain and tendon pathology.

### Non-surgical management

There were limited result findings for the conservative management of LHBT pathology due to the lack of articles found, with subjects managed non-surgically trending to return to work quicker but at lesser capacity and with less elbow/forearm strength than subjects undergoing LHB tenodesis. Similar to our observations, a systematic review by Castro, Corrêa [259] reported low to very-low-quality evidence to support the conservative management of rotator cuff and biceps tendinopathy-related shoulder pain. Generic rehabilitation principles for tendinopathy typically include progressive muscle strengthening for tendon remodelling and adaption to load [260, 261]. From the anecdotal research we found on biceps activity during common shoulder rehabilitation exercises, a potential LHB rehabilitation exercise program may include a progression of biceps curls into elevation, shoulder front raises in external rotation, elbow extension and supination, and a sport-specific program of overhead to underarm throwing drills. Similar authors have inferred the need for research validating the use of LHB strengthening exercises for rotator cuff-related shoulder pain, shoulder impingement and instability [30–32, 38, 50, 85, 88].

This review found no studies that compared LHB injection with no injection, indicating that the current literature does not provide guidance on the efficacy of injection for LHBT pathology. In contrast, a prior review reported that it might give temporary pain relief for inflammatory conditions of the LHBT [262]. If a clinical decision is made to use LHBT sheath injection, our results consistently indicate that image-guided procedures are more accurate and should be preferred over unguided techniques. Injected substances can migrate from the sheath into the communicating glenohumeral joint capsule, exposing the joint to a variety of potential corticosteroid adverse effects, including known catabolic effects on the tendon, ligament, adipose and cartilage tissue [262–264]. In addition, high concentrations of commonly used local anaesthetics such as lidocaine and bupivacaine are chondrotoxic [265–267]. These risks must be weighed against pain-induced morbidity and progressive shoulder dysfunction associated with LHBT pathology. Until further research clarifies the efficacy of injection compared with other treatment options, clinicians must continue to make clinical decisions based on the individual patient’s circumstances.

### Surgical management

For the surgical management of long head of biceps pathology with or without rotator cuff pathology, our scoping review found similar improvements in patient-reported outcomes for pain and function post-operatively for tenodesis or tenotomy, with tenodesis trending towards higher constant scores and tenotomy trending to be more cost and time effective. At the same time, patients undergoing tenodesis were less likely to have post-surgical complications of Popeye deformity and cramping arm pain. A similar trend was observed in a recent systematic review by Aldon-Villegas, Perez-Cabezas [268], with fewer Popeye signs and cramping arm pain following tenodesis compared with tenotomy. A slight trend was observed for reduced operative time, narcotic use and quicker pain relief in patients undergoing tenotomy. In studies that dichotomised their results by age, less satisfaction was observed in younger male patients following tenotomy compared with tenodesis.

The systematic inclusion of adjunctive LHB tenotomy or tenodesis on a healthy normal LHBT with rotator cuff repair has been reported as an increasing trend in the literature [269]. Our findings support avoiding adjunctive LHBT surgery with rotator cuff repairs in subjects with a normal LHBT because similar clinical outcomes following Supraspinatus repair in isolation vs. Supraspinatus repair with adjunctive biceps tenodesis/tenotomy were found. Similar patient-reported outcome measures (PROMS) in subjects undergoing rotator cuff repair and adjunctive tenodesis or tenotomy compared with rotator cuff repair in isolation were observed by Watson, Robbins [19]. Whether to include routine biceps tenodesis/tenotomy with rotator cuff repair presents a surgical conundrum similar to the standard inclusion of bursectomy with subacromial decompression surgery for subacromial pain syndrome when evidence supports identical clinical outcomes for bursectomy vs. bursectomy with acromioplasty [270]. The question of whether LHB tenodesis or tenotomy should be routinely performed in arthroscopic rotator cuff repairs was recently discussed by Moorthy and Tan [271], concluding that whilst there is a role for biceps surgery in rotator cuff repair, further higher-level studies are needed to determine if tenodesis and tenotomy should be performed routinely instead of selectively in rotator cuff tears. Goldfarb and Yamaguchi [6] advocated that routine tenodesis be avoided during operative treatment for rotator cuff tears as LHB tendinopathy may be reversible.

### Limitations

This scoping review was limited to English publications, excluding full-text articles published in other languages. Previous narrative reviews and grey literature were excluded, including conference abstracts and thesis dissertations. The lead author ^(BD)^ independently performed the screening, inclusion, exclusion, and data extraction. Whilst the level of evidence for each article was assessed, the methodological quality of the studies was not appraised critically as per guidelines for completing scoping reviews. Articles biased towards rotator cuff pathology and intra-articular SLAP or labral pathology were excluded from this scoping review.

### Conclusions and recommendations

The scoping review highlights; **(a) Anatomy** – Common anatomical variations of the biceps brachii, associated with shoulder pain and instability, a potential role for the biceps sling in LHBT stability with minimal support for the transverse humeral ligament as a separate, distinct anatomical structure. **(b) Function** - the minimal role of the LHB in shoulder elevation and glenohumeral stability in healthy shoulders. In pathological shoulders, proximal humeral migration, and hyperactivity of the LHB suggest a potential compensation function in individuals with symptomatic rotator cuff tears or shoulder instability. However, changes in LHB muscle activity in subjects with asymptomatic rotator cuff pathology are undetermined. Further research is required in both asymptomatic and symptomatic rotator cuff tear populations to determine whether there is a compensation role of the LHB at the shoulder. **(c) Pathology** - The prevalence of concomitant LHBT pathology with rotator cuff tears is well established; however, the cause-and-effect relationship between LHBT pathology and rotator cuff disease is undetermined. Further research is required to establish if the LHBT becomes overloaded and, in turn, pathological as it tries to compensate for the loss of function of a torn rotator cuff or is a secondary effect of degenerative shoulder pathology, pain, and weakness. **(d) Assessment** - The diagnostic utility of both clinical orthopaedic tests and diagnostic imaging to exclude LHBT pathology may be understated due to the inherent limitations of arthroscopy as the standard gold reference to visualise the proximal LHBT at the glenohumeral joint fully. **(e) Non-surgical management** - Conservative rehabilitation programs of the shoulder that include biceps strengthening protocols are understudied, and the evidence to support the inclusion of LHB rehabilitation in the management of rotator cuff and biceps-related shoulder pain is lacking. Further research is required to understand how a biceps-focused rehabilitation program may improve shoulder pain and functional outcomes in patients with symptomatic rotator cuff tears and b) prevent or reduce the likelihood of an asymptomatic rotator cuff tear becoming symptomatic and progressing to failure. **(f) Surgical management** - Similar post-surgical clinical outcomes are observed for tenodesis and tenotomy for biceps and concomitant rotator cuff-related shoulder pain, with subjects undergoing biceps tenodesis less likely to have cramping arm pain and a Popeye deformity compared to patients undergoing biceps tenotomy. The significance of routine surgical removal of the LHBT and sequelae on rotator cuff tear progression to failure and long-term shoulder function is unknown, and further research is required.

## Electronic supplementary material

Below is the link to the electronic supplementary material.

Supplementary Material 1

Supplementary Material 2

Supplementary Material 3

Supplementary Material 4

Supplementary Material 5

Supplementary Material 6

Supplementary Material 7

Supplementary Material 8

Supplementary Material 9

Supplementary Material 10

Supplementary Material 11

Supplementary Material 12

Supplementary Material 13

Supplementary Material 14

Supplementary Material 15

Supplementary Material 16

Supplementary Material 17

Supplementary Material 18

Supplementary Material 19

Supplementary Material 20

Supplementary Material 21

Supplementary Material 22

## Data Availability

All data generated or analysed during this study are included in this published article [and its supplementary information files].

## Abbreviations

ABS: Absence of LHB
BB: Biceps Brachii
DUS: Diagnostic Ultrasound
F: Female
N: Force
GHJ: Glenohumeral Joint
ISP: Infraspinatus
K: Kappa Coefficient
α: Krippendorff’s Alpha Coefficient
LOE: Level of Evidence
LHB: Long Head of Biceps
LHBT: Long Head of Biceps Tendon
M: Male
NA: Not Applicable
n: Number
PROMS: Patient Reported Outcome Measures
p: P-value
PCC: Pearson’s Correlation Coefficient
ROM: Range of Motion
RC: Rotator Cuff
RCR: Rotator Cuff Repair
SHB: Short Head of Biceps
r: Spearman’s Rank Correlation Coefficient
ρ: Spearman’s correlation coefficient
SLAP: Superior Labrum Anterior Posterior
SSC: Subscapularis
SSP: Supraspinatus
TD: Tenodesis
TT: Tenotomy
