# Supplementary material for: The long head of biceps at the shoulder: a scoping review
Source: BMC Musculoskelet Disord. 2023 Mar 28;24:232. doi: 10.1186/s12891-023-06346-5 (PMC10044783; doi:10.1186/s12891-023-06346-5)
Supplement: Supplementary file 1 — Supplementary Material 1 [file 12891_2023_6346_MOESM1_ESM.docx]

# Additional file 2: Appendix 1_BMC.docx; Search strategy for the scoping review

## Basic search strategy (scoping and systematic reviews)

### Key concepts

| Concept 1 | Concept 2 | Concept 3 | Concept 4 |
| --- | --- | --- | --- |
| “Long head of biceps” | role | shoulder | management |

### Major search topics

| (“long head of biceps” OR LHB) | (Role OR function) | (Shoulder OR GHJ) | management |
| --- | --- | --- | --- |

### Basic search strategy

| (“long head of biceps” OR LHB) **AND** (role OR function) **AND** (shoulder OR GHJ) **AND** (management) |
| --- |

## Initial Basic search strategy to identify similar reviews

| Database | Date | URL search of similar studies found | Hits |
| --- | --- | --- | --- |
| PubMed | 30/07/21 | <https://pubmed.ncbi.nlm.nih.gov/?term=%28%E2%80%9Clong+head+of+biceps%E2%80%9D+OR+LHB%29+AND+%28role+OR+function%29+AND+%28shoulder+OR+GHJ%29+AND+%28management%29> | 1  [1]  Literature review only |
| Epistemonikos | 30/07/21 | <https://www.epistemonikos.org/en/search?q=%28%E2%80%9Clong+head+of+biceps%E2%80%9D+OR+LHB%29+AND+%28role+OR+function%29+AND+%28shoulder+OR+GHJ%29+AND+%28management%29> | 0 |
| PROSPERO | 30/07/21 | <https://www.crd.york.ac.uk/prospero/#searchadvanced> | 0 |
| OSF | 30/07/21 | <https://osf.io/search/?q=(%E2%80%9Clong%20head%20of%20biceps%E2%80%9D%20OR%20LHB)%20AND%20(role%20OR%20function)%20AND%20(shoulder%20OR%20GHJ)%20AND%20(management)&page=1> | 0 |

| Database | Date | URL Search | No of Results | Comments |
| --- | --- | --- | --- | --- |
| PubMed | 30/07/21 | <https://pubmed.ncbi.nlm.nih.gov/?term=%28%E2%80%9Clong+head+of+biceps%E2%80%9D+OR+LHB%29+AND+%28role+OR+function%29+AND+%28shoulder+OR+GHJ%29+AND+%28management%29> | 30 | Four relevant studies.   1. [2]. 2. [1]. 3. [3] 4. [4] |
| Google Scholar | 30/07/21 | <https://scholar.google.com/scholar?hl=en&as_sdt=0%2C5&q=%28%E2%80%9Clong+head+of+biceps%E2%80%9D+OR+LHB%29+AND+%28role+OR+function%29+AND+%28shoulder+OR+GHJ%29+AND+%28management%29&btnG=> | 4,280 | Many studies look at one concept. No studies looking at all concepts |

## Advanced search strategy for PubMed

| **Main concepts** | **Concept 1** | **Concept 2** | **Concept 3** | **Concept 4** |
| --- | --- | --- | --- | --- |
| Synonyms  joined with OR  and with  PubMed  title/abstract  field codes  and wild cards* | (“long head of biceps”[Title/Abstract] OR LHB[Title/Abstract] OR “biceps brachii”[Title/Abstract] OR “biceps sling”[Title/Abstract] OR “biceps pulley”[Title/Abstract] OR “superior labrum anterior and posterior”[Title/Abstract] OR SLAP[Title/Abstract] OR (biceps[Title/Abstract] AND tendin*[Title/Abstract]) OR (biceps[Title/Abstract] AND sublux*[Title/Abstract]) OR (biceps[Title/Abstract] AND tear[Title/Abstract]) OR (biceps[Title/Abstract] AND pain[Title/Abstract])) | (role[Title/Abstract] OR function[Title/Abstract] OR dysfunction[Title/Abstract] OR EMG[Title/Abstract] OR electromyography[Title/Abstract] OR anatomy[Title/Abstract] OR compensat*[Title/Abstract] OR substitut*[Title/Abstract] OR adapt*[Title/Abstract] OR overactiv*[Title/Abstract] OR hyperactiv*[Title/Abstract] OR underactiv*[Title/Abstract] OR hypoactiv*[Title/Abstract] OR diagnosis[Title/Abstract] OR pathology[Title/Abstract] OR symptomology[Title/Abstract] OR tests[Title/Abstract] OR imaging[Title/Abstract] OR biomechanic*[Title/Abstract] OR arthrokinematics[Title/Abstract] OR proprioception[Title/Abstract] OR kinematics[Title/Abstract] OR “firing patterns”[Title/Abstract] OR “range of motion”[Title/Abstract] OR ROM[Title/Abstract] OR recruit*[Title/Abstract] OR activity[Title/Abstract] OR (muscle[Title/Abstract] AND activation[Title/Abstract]) OR (movement[Title/Abstract] AND patterns[Title/Abstract]) OR elevation[Title/Abstract] OR reach*[Title/Abstract] OR “activities of daily living”[Title/Abstract] OR ADL[Title/Abstract] OR coactivate*[Title/Abstract] OR synergistic[Title/Abstract] OR strength[Title/Abstract] OR weakness[Title/Abstract] OR stability[Title/Abstract] OR depressor[Title/Abstract] OR instability[Title/Abstract] OR stable[Title/Abstract] OR unstable[Title/Abstract] OR stabili*[Title/Abstract] OR sublux*[Title/Abstract] OR dislocate*[Title/Abstract] OR enlocat*[Title/Abstract] OR migrat*[Title/Abstract] OR translat*[Title/Abstract] OR ultrasound[Title/Abstract] OR US[Title/Abstract] OR “diagnostic ultrasound”[Title/Abstract] OR DUS[Title/Abstract] OR “magnetic resonance imaging”[Title/Abstract] OR MRI[Title/Abstract] OR MR[Title/Abstract] OR MRA[Title/Abstract] OR arthrogram[Title/Abstract] OR arthroscopy[Title/Abstract] OR “orthopaedic special tests”[Title/Abstract] OR OST[Title/Abstract] OR test*[Title/Abstract] OR assess*[Title/Abstract] OR exam*[Title/Abstract] OR physiology[Title/Abstract] OR pathophysiology[Title/Abstract]) | (shoulder[Title/Abstract] OR GHJ[Title/Abstract] OR GHJ[Title/Abstract] OR “HOH”[Title/Abstract] OR “head of humerus”[Title/Abstract] OR HOH[Title/Abstract] OR “proximal humeral”[Title/Abstract] OR “RC”[Title/Abstract] OR “RC interval”[Title/Abstract] OR “RC cable”[Title/Abstract] OR supraspinatus[Title/Abstract] OR infraspinatus[Title/Abstract] OR subscapularis[Title/Abstract] OR teres minor[Title/Abstract] OR deltoid[Title/Abstract]) | (management[Title/Abstract] OR treatment[Title/Abstract] OR intervention[Title/Abstract] OR “evidence based practice”[Title/Abstract] OR surg*[Title/Abstract] OR non-surg*[Title/Abstract] OR operat*[Title/Abstract] OR “post-operative”[Title/Abstract] OR “post-surgical”[Title/Abstract] OR procedure[Title/Abstract] OR physiotherapy[Title/Abstract] OR “physical therapy”[Title/Abstract] OR therapy[Title/Abstract] OR rehab*[Title/Abstract] OR conservative[Title/Abstract] OR tenodesis[Title/Abstract] OR tenotomy[Title/Abstract] OR orthopaedic*[Title/Abstract]) |
| MeSH terms  joined with OR |  | (“Electromyography”[Mesh] OR  “Physiology”[Mesh] OR “physiopathology”[Subheading] OR  “Anatomy”[Mesh] OR  “Magnetic Resonance Imaging”[Mesh] OR “Diagnosis, Differential”[Mesh] OR “Physical Examination”[Mesh] OR Diagnosis”[Mesh]) OR “diagnosis” [Subheading] OR “diagnostic imaging”[Subheading] OR “ultrasonography”[MeSH Terms] OR “ultrasonics”[MeSH Terms]) | (“Shoulder”[Mesh]  OR “Shoulder Joint”[Mesh]) | (“Evidence-Based Practice”[Mesh] OR “Physical Therapy Modalities”[Mesh] OR “Orthopedics”[Mesh] OR “Surgical Procedures, Operative”[Mesh] OR “General Surgery”[Mesh] OR “Treatment Outcome”[Mesh] OR “Orthopedic Procedures”[Mesh] OR “Exercise Therapy”[Mesh] OR “Conservative Treatment”[Mesh] OR “Tenodesis”[Mesh] OR “Tenotomy”[Mesh] OR “Arthroscopy”[Mesh]) |

## PubMed search strategy

(“long head of biceps”[Title/Abstract] OR LHB[Title/Abstract] OR “biceps brachii”[Title/Abstract] OR “biceps sling”[Title/Abstract] OR “biceps pulley”[Title/Abstract] OR “superior labrum anterior and posterior”[Title/Abstract] OR SLAP[Title/Abstract] OR (biceps[Title/Abstract] AND tendin*[Title/Abstract]) OR (biceps[Title/Abstract] AND sublux*[Title/Abstract]) OR (biceps[Title/Abstract] AND tear[Title/Abstract]) OR (biceps[Title/Abstract] AND pain[Title/Abstract])) AND (role[Title/Abstract] OR function[Title/Abstract] OR dysfunction[Title/Abstract] OR EMG[Title/Abstract] OR electromyography[Title/Abstract] OR anatomy[Title/Abstract] OR compensat*[Title/Abstract] OR substitut*[Title/Abstract] OR adapt*[Title/Abstract] OR overactiv*[Title/Abstract] OR hyperactiv*[Title/Abstract] OR underactiv*[Title/Abstract] OR hypoactiv*[Title/Abstract] OR diagnosis[Title/Abstract] OR pathology[Title/Abstract] OR symptomology[Title/Abstract] OR tests[Title/Abstract] OR imaging[Title/Abstract] OR biomechanic*[Title/Abstract] OR arthrokinematics[Title/Abstract] OR proprioception[Title/Abstract] OR kinematics[Title/Abstract] OR “firing patterns”[Title/Abstract] OR “range of motion”[Title/Abstract] OR ROM[Title/Abstract] OR recruit*[Title/Abstract] OR activity[Title/Abstract] OR (muscle[Title/Abstract] AND activation[Title/Abstract]) OR (movement[Title/Abstract] AND patterns[Title/Abstract]) OR elevation[Title/Abstract] OR reach*[Title/Abstract] OR “activities of daily living”[Title/Abstract] OR ADL[Title/Abstract] OR coactivate*[Title/Abstract] OR synergistic[Title/Abstract] OR strength[Title/Abstract] OR weakness[Title/Abstract] OR stability[Title/Abstract] OR depressor[Title/Abstract] OR instability[Title/Abstract] OR stable[Title/Abstract] OR unstable[Title/Abstract] OR stabili*[Title/Abstract] OR sublux*[Title/Abstract] OR dislocate*[Title/Abstract] OR enlocat*[Title/Abstract] OR migrat*[Title/Abstract] OR translat*[Title/Abstract] OR ultrasound[Title/Abstract] OR US[Title/Abstract] OR “diagnostic ultrasound”[Title/Abstract] OR DUS[Title/Abstract] OR “magnetic resonance imaging”[Title/Abstract] OR MRI[Title/Abstract] OR MR[Title/Abstract] OR MRA[Title/Abstract] OR arthrogram[Title/Abstract] OR arthroscopy[Title/Abstract] OR “orthopaedic special tests”[Title/Abstract] OR OST[Title/Abstract] OR test*[Title/Abstract] OR assess*[Title/Abstract] OR exam*[Title/Abstract] OR physiology[Title/Abstract] OR pathophysiology[Title/Abstract] OR (“Electromyography”[Mesh] OR “Physiology”[Mesh] OR “physiopathology”[Subheading] OR “Anatomy”[Mesh] OR “Magnetic Resonance Imaging”[Mesh] OR “Diagnosis, Differential”[Mesh] OR “Physical Examination”[Mesh] OR “Diagnosis”[Mesh] OR “diagnosis”[Subheading] OR “diagnostic imaging”[Subheading] OR “ultrasonography”[MeSH Terms] OR “ultrasonics”[MeSH Terms]) AND (shoulder[Title/Abstract] OR GHJ[Title/Abstract] OR GHJ[Title/Abstract] OR “HOH”[Title/Abstract] OR “head of humerus”[Title/Abstract] OR HOH[Title/Abstract] OR “proximal humeral”[Title/Abstract] OR “RC”[Title/Abstract] OR “RC interval”[Title/Abstract] OR “RC cable”[Title/Abstract] OR supraspinatus[Title/Abstract] OR infraspinatus[Title/Abstract] OR subscapularis[Title/Abstract] OR “teres minor”[Title/Abstract] OR deltoid[Title/Abstract] OR “Shoulder”[Mesh] OR “Shoulder Joint”[Mesh]) AND (management[Title/Abstract] OR treatment[Title/Abstract] OR intervention[Title/Abstract] OR “evidence based practice”[Title/Abstract] OR surg*[Title/Abstract] OR non-surg*[Title/Abstract] OR operat*[Title/Abstract] OR “post-operative”[Title/Abstract] OR “post-surgical”[Title/Abstract] OR procedure[Title/Abstract] OR physiotherapy[Title/Abstract] OR “physical therapy”[Title/Abstract] OR therapy[Title/Abstract] OR rehab*[Title/Abstract] OR conservative[Title/Abstract] OR tenodesis[Title/Abstract] OR tenotomy[Title/Abstract] OR orthopaedic*[Title/Abstract] OR “Evidence-Based Practice”[Mesh] OR “Physical Therapy Modalities”[Mesh] OR “Orthopedics”[Mesh] OR “Surgical Procedures, Operative”[Mesh] OR “General Surgery”[Mesh] OR “Treatment Outcome”[Mesh] OR “Orthopedic Procedures”[Mesh] OR “Exercise Therapy”[Mesh] OR “Conservative Treatment”[Mesh] OR “Tenodesis”[Mesh] OR “Tenotomy”[Mesh] OR “Arthroscopy”[Mesh])

## Search log by database

| **Database** | **Date searched** | **Search strategy** | **No of results** | **URL** |
| --- | --- | --- | --- | --- |
| MEDLINE on PubMed | 16/09/2021 | (“long head of biceps”[tiab] OR LHB[tiab] OR “biceps brachii”[tiab] OR “biceps sling”[tiab] OR “biceps pulley”[tiab] OR “superior labrum anterior and posterior”[tiab] OR SLAP[tiab] OR (biceps[tiab] AND tendin*[tiab]) OR (biceps[tiab] AND sublux*[tiab]) OR (biceps[tiab] AND tear[tiab]) OR (biceps[tiab] AND pain[tiab])) AND (role[tiab] OR function[tiab] OR dysfunction[tiab] OR EMG[tiab] OR electromyography[tiab] OR anatomy[tiab] OR compensat*[tiab] OR substitut*[tiab] OR adapt*[tiab] OR overactiv*[tiab] OR hyperactiv*[tiab] OR underactiv*[tiab] OR hypoactiv*[tiab] OR diagnosis[tiab] OR pathology[tiab] OR symptomology[tiab] OR tests[tiab] OR imaging[tiab] OR biomechanic*[tiab] OR arthrokinematics[tiab] OR proprioception[tiab] OR kinematics[tiab] OR “firing patterns”[tiab] OR “range of motion”[tiab] OR ROM[tiab] OR recruit*[tiab] OR activity[tiab] OR (muscle[tiab] AND activation[tiab]) OR (movement[tiab] AND patterns[tiab]) OR elevation[tiab] OR reach*[tiab] OR “activities of daily living”[tiab] OR ADL[tiab] OR coactivate*[tiab] OR synergistic[tiab] OR strength[tiab] OR weakness[tiab] OR stability[tiab] OR depressor[tiab] OR instability[tiab] OR stable[tiab] OR unstable[tiab] OR stabili*[tiab] OR sublux*[tiab] OR dislocate*[tiab] OR enlocat*[tiab] OR migrat*[tiab] OR translat*[tiab] OR ultrasound[tiab] OR US[tiab] OR “diagnostic ultrasound”[tiab] OR DUS[tiab] OR “magnetic resonance imaging”[tiab] OR MRI[tiab] OR MR[tiab] OR MRA[tiab] OR arthrogram[tiab] OR arthroscopy[tiab] OR “orthopaedic special tests”[tiab] OR OST[tiab] OR test*[tiab] OR assess*[tiab] OR exam*[tiab] OR physiology[tiab] OR pathophysiology[tiab] OR (Electromyography[Mesh] OR Physiology[Mesh] OR “Physiopathology”[sh] OR Anatomy[Mesh] OR “Magnetic Resonance Imaging”[Mesh] OR “Diagnosis, Differential”[Mesh] OR “Physical Examination”[Mesh] OR Diagnosis[Mesh] OR “Diagnosis”[sh] OR “Diagnostic Imaging”[sh] OR ultrasonography[Mesh] OR ultrasonics[Mesh]) AND (shoulder[tiab] OR GHJ[tiab] OR GHJ[tiab] OR “HOH”[tiab] OR “head of humerus”[tiab] OR HOH[tiab] OR “proximal humeral”[tiab] OR “RC”[tiab] OR “RC interval”[tiab] OR “RC cable”[tiab] OR supraspinatus[tiab] OR infraspinatus[tiab] OR subscapularis[tiab] OR “teres minor”[tiab] OR deltoid[tiab] OR Shoulder[Mesh] OR “Shoulder Joint”[Mesh]) AND (management[tiab] OR treatment[tiab] OR intervention[tiab] OR “evidence based practice”[tiab] OR surg*[tiab] OR non-surg*[tiab] OR operat*[tiab] OR post-operative[tiab] OR post-surgical[tiab] OR procedure[tiab] OR physiotherapy[tiab] OR “physical therapy”[tiab] OR therapy[tiab] OR rehab*[tiab] OR conservative[tiab] OR tenodesis[tiab] OR tenotomy[tiab] OR orthopaedic*[tiab] OR “Evidence-Based Practice”[Mesh] OR “Physical Therapy Modalities”[Mesh] OR Orthopedics[Mesh] OR “Surgical Procedures, Operative”[Mesh] OR “General Surgery”[Mesh] OR “Treatment Outcome”[Mesh] OR “Orthopedic Procedures”[Mesh] OR “Exercise Therapy”[Mesh] OR “Conservative Treatment”[Mesh] OR Tenodesis[Mesh] OR Tenotomy[Mesh] OR Arthroscopy[Mesh])) | 2,110 | <https://pubmed.ncbi.nlm.nih.gov/searches/6188787/?mode=full&sort=date> |
| Embase (Elsevier) | 16/09/2021 | (“long head of biceps”:ti,ab OR LHB:ti,ab OR “biceps brachii”:ti,ab OR “biceps sling”:ti,ab OR “biceps pulley”:ti,ab OR “superior labrum anterior and posterior”:ti,ab OR SLAP:ti,ab OR (biceps:ti,ab AND tendin*:ti,ab) OR (biceps:ti,ab AND sublux*:ti,ab) OR (biceps:ti,ab AND tear:ti,ab) OR (biceps:ti,ab AND pain:ti,ab)) AND (role:ti,ab OR function:ti,ab OR dysfunction:ti,ab OR EMG:ti,ab OR electromyography:ti,ab OR anatomy:ti,ab OR compensat*:ti,ab OR substitut*:ti,ab OR adapt*:ti,ab OR overactiv*:ti,ab OR hyperactiv*:ti,ab OR underactiv*:ti,ab OR hypoactiv*:ti,ab OR diagnosis:ti,ab OR pathology:ti,ab OR symptomology:ti,ab OR tests:ti,ab OR imaging:ti,ab OR biomechanic*:ti,ab OR arthrokinematics:ti,ab OR proprioception:ti,ab OR kinematics:ti,ab OR “firing patterns”:ti,ab OR “range of motion”:ti,ab OR ROM:ti,ab OR recruit*:ti,ab OR activity:ti,ab OR (muscle:ti,ab AND activation:ti,ab) OR (movement:ti,ab AND patterns:ti,ab) OR elevation:ti,ab OR reach*:ti,ab OR “activities of daily living”:ti,ab OR ADL:ti,ab OR coactivate*:ti,ab OR synergistic:ti,ab OR strength:ti,ab OR weakness:ti,ab OR stability:ti,ab OR depressor:ti,ab OR instability:ti,ab OR stable:ti,ab OR unstable:ti,ab OR stabili*:ti,ab OR sublux*:ti,ab OR dislocate*:ti,ab OR enlocat*:ti,ab OR migrat*:ti,ab OR translat*:ti,ab OR ultrasound:ti,ab OR US:ti,ab OR “diagnostic ultrasound”:ti,ab OR DUS:ti,ab OR “magnetic resonance imaging”:ti,ab OR MRI:ti,ab OR MR:ti,ab OR MRA:ti,ab OR arthrogram:ti,ab OR arthroscopy:ti,ab OR “orthopaedic special tests”:ti,ab OR OST:ti,ab OR test*:ti,ab OR assess*:ti,ab OR exam*:ti,ab OR physiology:ti,ab OR pathophysiology:ti,ab OR (Electromyography/exp OR Physiology/exp OR “Physiopathology” OR Anatomy/exp OR “Magnetic Resonance Imaging”/exp OR “Diagnosis, Differential”/exp OR “Physical Examination”/exp OR Diagnosis/exp OR “Diagnosis” OR “Diagnostic Imaging” OR ultrasonography/exp OR ultrasonics/exp) AND (shoulder:ti,ab OR GHJ:ti,ab OR GHJ:ti,ab OR “HOH”:ti,ab OR “head of humerus”:ti,ab OR HOH:ti,ab OR “proximal humeral”:ti,ab OR “RC”:ti,ab OR “RC interval”:ti,ab OR “RC cable”:ti,ab OR supraspinatus:ti,ab OR infraspinatus:ti,ab OR subscapularis:ti,ab OR “teres minor”:ti,ab OR deltoid:ti,ab OR Shoulder/exp OR “Shoulder Joint”/exp) AND (management:ti,ab OR treatment:ti,ab OR intervention:ti,ab OR “evidence based practice”:ti,ab OR surg*:ti,ab OR non-surg*:ti,ab OR operat*:ti,ab OR post-operative:ti,ab OR post-surgical:ti,ab OR procedure:ti,ab OR physiotherapy:ti,ab OR “physical therapy”:ti,ab OR therapy:ti,ab OR rehab*:ti,ab OR conservative:ti,ab OR tenodesis:ti,ab OR tenotomy:ti,ab OR orthopaedic*:ti,ab OR “Evidence-Based Practice”/exp OR “Physical Therapy Modalities”/exp OR Orthopedics/exp OR “Surgical Procedures, Operative”/exp OR “General Surgery”/exp OR “Treatment Outcome”/exp OR “Orthopedic Procedures”/exp OR “Exercise Therapy”/exp OR “Conservative Treatment”/exp OR Tenodesis/exp OR Tenotomy/exp OR Arthroscopy/exp)) | 2416 | <https://www-embase-com.ezproxy.bond.edu.au/#advancedSearch/resultspage> |
| Cinahl (Ebsco) | 16/09/2021 | ((TI “long head of biceps” OR AB “long head of biceps”) OR (TI LHB OR AB LHB) OR (TI “biceps brachii” OR AB “biceps brachii”) OR (TI “biceps sling” OR AB “biceps sling”) OR (TI “biceps pulley” OR AB “biceps pulley”) OR (TI “superior labrum anterior and posterior” OR AB “superior labrum anterior and posterior”) OR (TI SLAP OR AB SLAP) OR ((TI biceps OR AB biceps) AND (TI tendin* OR AB tendin*)) OR ((TI biceps OR AB biceps) AND (TI sublux* OR AB sublux*)) OR ((TI biceps OR AB biceps) AND (TI tear OR AB tear)) OR ((TI biceps OR AB biceps) AND (TI pain OR AB pain))) AND ((TI role OR AB role) OR (TI function OR AB function) OR (TI dysfunction OR AB dysfunction) OR (TI EMG OR AB EMG) OR (TI electromyography OR AB electromyography) OR (TI anatomy OR AB anatomy) OR (TI compensat* OR AB compensat*) OR (TI substitut* OR AB substitut*) OR (TI adapt* OR AB adapt*) OR (TI overactiv* OR AB overactiv*) OR (TI hyperactiv* OR AB hyperactiv*) OR (TI underactiv* OR AB underactiv*) OR (TI hypoactiv* OR AB hypoactiv*) OR (TI diagnosis OR AB diagnosis) OR (TI pathology OR AB pathology) OR (TI symptomology OR AB symptomology) OR (TI tests OR AB tests) OR (TI imaging OR AB imaging) OR (TI biomechanic* OR AB biomechanic*) OR (TI arthrokinematics OR AB arthrokinematics) OR (TI proprioception OR AB proprioception) OR (TI kinematics OR AB kinematics) OR (TI “firing patterns” OR AB “firing patterns”) OR (TI “range of motion” OR AB “range of motion”) OR (TI ROM OR AB ROM) OR (TI recruit* OR AB recruit*) OR (TI activity OR AB activity) OR ((TI muscle OR AB muscle) AND (TI activation OR AB activation)) OR ((TI movement OR AB movement) AND (TI patterns OR AB patterns)) OR (TI elevation OR AB elevation) OR (TI reach* OR AB reach*) OR (TI “activities of daily living” OR AB “activities of daily living”) OR (TI ADL OR AB ADL) OR (TI coactivate* OR AB coactivate*) OR (TI synergistic OR AB synergistic) OR (TI strength OR AB strength) OR (TI weakness OR AB weakness) OR (TI stability OR AB stability) OR (TI depressor OR AB depressor) OR (TI instability OR AB instability) OR (TI stable OR AB stable) OR (TI unstable OR AB unstable) OR (TI stabili* OR AB stabili*) OR (TI sublux* OR AB sublux*) OR (TI dislocate* OR AB dislocate*) OR (TI enlocat* OR AB enlocat*) OR (TI migrat* OR AB migrat*) OR (TI translat* OR AB translat*) OR (TI ultrasound OR AB ultrasound) OR (TI US OR AB US) OR (TI “diagnostic ultrasound” OR AB “diagnostic ultrasound”) OR (TI DUS OR AB DUS) OR (TI “magnetic resonance imaging” OR AB “magnetic resonance imaging”) OR (TI MRI OR AB MRI) OR (TI MR OR AB MR) OR (TI MRA OR AB MRA) OR (TI arthrogram OR AB arthrogram) OR (TI arthroscopy OR AB arthroscopy) OR (TI “orthopaedic special tests” OR AB “orthopaedic special tests”) OR (TI OST OR AB OST) OR (TI test* OR AB test*) OR (TI assess* OR AB assess*) OR (TI exam* OR AB exam*) OR (TI physiology OR AB physiology) OR (TI pathophysiology OR AB pathophysiology) OR ((MH Electromyography+) OR (MH Physiology+) OR “Physiopathology” OR (MH Anatomy+) OR (MH “Magnetic Resonance Imaging”+) OR (MH “Diagnosis, Differential”+) OR (MH “Physical Examination”+) OR (MH Diagnosis+) OR “Diagnosis” OR “Diagnostic Imaging” OR (MH ultrasonography+) OR (MH ultrasonics+)) AND ((TI shoulder OR AB shoulder) OR (TI GHJ OR AB GHJ) OR (TI GHJ OR AB GHJ) OR (TI “HOH” OR AB “HOH”) OR (TI “head of humerus” OR AB “head of humerus”) OR (TI HOH OR AB HOH) OR (TI “proximal humeral” OR AB “proximal humeral”) OR (TI “RC” OR AB “RC”) OR (TI “RC interval” OR AB “RC interval”) OR (TI “RC cable” OR AB “RC cable”) OR (TI supraspinatus OR AB supraspinatus) OR (TI infraspinatus OR AB infraspinatus) OR (TI subscapularis OR AB subscapularis) OR (TI “teres minor” OR AB “teres minor”) OR (TI deltoid OR AB deltoid) OR (MH Shoulder+) OR (MH “Shoulder Joint”+)) AND ((TI management OR AB management) OR (TI treatment OR AB treatment) OR (TI intervention OR AB intervention) OR (TI “evidence based practice” OR AB “evidence based practice”) OR (TI surg* OR AB surg*) OR (TI non-surg* OR AB non-surg*) OR (TI operat* OR AB operat*) OR (TI post-operative OR AB post-operative) OR (TI post-surgical OR AB post-surgical) OR (TI procedure OR AB procedure) OR (TI physiotherapy OR AB physiotherapy) OR (TI “physical therapy” OR AB “physical therapy”) OR (TI therapy OR AB therapy) OR (TI rehab* OR AB rehab*) OR (TI conservative OR AB conservative) OR (TI tenodesis OR AB tenodesis) OR (TI tenotomy OR AB tenotomy) OR (TI orthopaedic* OR AB orthopaedic*) OR (MH “Evidence-Based Practice”+) OR (MH “Physical Therapy Modalities”+) OR (MH Orthopedics+) OR (MH “Surgical Procedures, Operative”+) OR (MH “General Surgery”+) OR (MH “Treatment Outcome”+) OR (MH “Orthopedic Procedures”+) OR (MH “Exercise Therapy”+) OR (MH “Conservative Treatment”+) OR (MH Tenodesis+) OR (MH Tenotomy+) OR (MH Arthroscopy+))) | 445 | <https://web.a.ebscohost.com/ehost/resultsadvanced?vid=27&sid=121acb93-e0c1-485f-b537-4fe89f1870da%40sdc-v-sessmgr01> |
| SPORTDiscus | 16/09/2021 | ((TI “long head of biceps” OR AB “long head of biceps”) OR (TI “LHB” OR AB “LHB”) OR (TI “biceps brachii” OR AB “biceps brachii”) OR (TI “biceps sling” OR AB “biceps sling”) OR (TI “biceps pulley” OR AB “biceps pulley”) OR (TI “superior labrum anterior and posterior” OR AB “superior labrum anterior and posterior”) OR (TI “SLAP” OR AB “SLAP”) OR ((TI “biceps” OR AB “biceps”) AND (TI “tendin*” OR AB “tendin*”)) OR ((TI “biceps” OR AB “biceps”) AND (TI “sublux*” OR AB “sublux*”)) OR ((TI “biceps” OR AB “biceps”) AND (TI “tear” OR AB “tear”)) OR ((TI “biceps” OR AB “biceps”) AND (TI “pain” OR AB “pain”))) AND ((TI “role” OR AB “role”) OR (TI “function” OR AB “function”) OR (TI “dysfunction” OR AB “dysfunction”) OR (TI “EMG” OR AB “EMG”) OR (TI “electromyography” OR AB “electromyography”) OR (TI “anatomy” OR AB “anatomy”) OR (TI “compensat*” OR AB “compensat*”) OR (TI “substitut*” OR AB “substitut*”) OR (TI “adapt*” OR AB “adapt*”) OR (TI “overactiv*” OR AB “overactiv*”) OR (TI “hyperactiv*” OR AB “hyperactiv*”) OR (TI “underactiv*” OR AB “underactiv*”) OR (TI “hypoactiv*” OR AB “hypoactiv*”) OR (TI “diagnosis” OR AB “diagnosis”) OR (TI “pathology” OR AB “pathology”) OR (TI “symptomology” OR AB “symptomology”) OR (TI “tests” OR AB “tests”) OR (TI “imaging” OR AB “imaging”) OR (TI “biomechanic*” OR AB “biomechanic*”) OR (TI “arthrokinematics” OR AB “arthrokinematics”) OR (TI “proprioception” OR AB “proprioception”) OR (TI “kinematics” OR AB “kinematics”) OR (TI “firing patterns” OR AB “firing patterns”) OR (TI “range of motion” OR AB “range of motion”) OR (TI “ROM” OR AB “ROM”) OR (TI “recruit*” OR AB “recruit*”) OR (TI “activity” OR AB “activity”) OR ((TI “muscle” OR AB “muscle”) AND (TI “activation” OR AB “activation”)) OR ((TI “movement” OR AB “movement”) AND (TI “patterns” OR AB “patterns”)) OR (TI “elevation” OR AB “elevation”) OR (TI “reach*” OR AB “reach*”) OR (TI “activities of daily living” OR AB “activities of daily living”) OR (TI “ADL” OR AB “ADL”) OR (TI “coactivate*” OR AB “coactivate*”) OR (TI “synergistic” OR AB “synergistic”) OR (TI “strength” OR AB “strength”) OR (TI “weakness” OR AB “weakness”) OR (TI “stability” OR AB “stability”) OR (TI “depressor” OR AB “depressor”) OR (TI “instability” OR AB “instability”) OR (TI “stable” OR AB “stable”) OR (TI “unstable” OR AB “unstable”) OR (TI “stabili*” OR AB “stabili*”) OR (TI “sublux*” OR AB “sublux*”) OR (TI “dislocate*” OR AB “dislocate*”) OR (TI “enlocat*” OR AB “enlocat*”) OR (TI “migrat*” OR AB “migrat*”) OR (TI “translat*” OR AB “translat*”) OR (TI “ultrasound” OR AB “ultrasound”) OR (TI “US” OR AB “US”) OR (TI “diagnostic ultrasound” OR AB “diagnostic ultrasound”) OR (TI “DUS” OR AB “DUS”) OR (TI “magnetic resonance imaging” OR AB “magnetic resonance imaging”) OR (TI “MRI” OR AB “MRI”) OR (TI “MR” OR AB “MR”) OR (TI “MRA” OR AB “MRA”) OR (TI “arthrogram” OR AB “arthrogram”) OR (TI “arthroscopy” OR AB “arthroscopy”) OR (TI “orthopaedic special tests” OR AB “orthopaedic special tests”) OR (TI “OST” OR AB “OST”) OR (TI “test*” OR AB “test*”) OR (TI “assess*” OR AB “assess*”) OR (TI “exam*” OR AB “exam*”) OR (TI “physiology” OR AB “physiology”) OR (TI “pathophysiology” OR AB “pathophysiology”) OR (DE “Electromyography” OR DE “Physiology” OR “Physiopathology” OR DE “Anatomy” OR DE “Magnetic Resonance Imaging” OR DE “Diagnosis, Differential” OR DE “Physical Examination” OR DE “Diagnosis” OR “Diagnosis” OR “Diagnostic Imaging” OR DE “ultrasonography” OR DE “ultrasonics”) AND ((TI “shoulder” OR AB “shoulder”) OR (TI “GHJ” OR AB “GHJ”) OR (TI “GHJ” OR AB “GHJ”) OR (TI “HOH” OR AB “HOH”) OR (TI “head of humerus” OR AB “head of humerus”) OR (TI “HOH” OR AB “HOH”) OR (TI “proximal humeral” OR AB “proximal humeral”) OR (TI “RC” OR AB “RC”) OR (TI “RC interval” OR AB “RC interval”) OR (TI “RC cable” OR AB “RC cable”) OR (TI “supraspinatus” OR AB “supraspinatus”) OR (TI “infraspinatus” OR AB “infraspinatus”) OR (TI “subscapularis” OR AB “subscapularis”) OR (TI “teres minor” OR AB “teres minor”) OR (TI “deltoid” OR AB “deltoid”) OR DE “Shoulder” OR DE “Shoulder Joint”) AND ((TI “management” OR AB “management”) OR (TI “treatment” OR AB “treatment”) OR (TI “intervention” OR AB “intervention”) OR (TI “evidence based practice” OR AB “evidence based practice”) OR (TI “surg*” OR AB “surg*”) OR (TI “non-surg*” OR AB “non-surg*”) OR (TI “operat*” OR AB “operat*”) OR (TI “post-operative” OR AB “post-operative”) OR (TI “post-surgical” OR AB “post-surgical”) OR (TI “procedure” OR AB “procedure”) OR (TI “physiotherapy” OR AB “physiotherapy”) OR (TI “physical therapy” OR AB “physical therapy”) OR (TI “therapy” OR AB “therapy”) OR (TI “rehab*” OR AB “rehab*”) OR (TI “conservative” OR AB “conservative”) OR (TI “tenodesis” OR AB “tenodesis”) OR (TI “tenotomy” OR AB “tenotomy”) OR (TI “orthopaedic*” OR AB “orthopaedic*”) OR DE “Evidence-Based Practice” OR DE “Physical Therapy Modalities” OR DE “Orthopedics” OR DE “Surgical Procedures, Operative” OR DE “General Surgery” OR DE “Treatment Outcome” OR DE “Orthopedic Procedures” OR DE “Exercise Therapy” OR DE “Conservative Treatment” OR DE “Tenodesis” OR DE “Tenotomy” OR DE “Arthroscopy”)) | 419 | <https://web.a.ebscohost.com/ehost/resultsadvanced?vid=8&sid=121acb93-e0c1-485f-b537-4fe89f1870da%40sdc-v-sessmgr01> |
| CENTRAL | 16/09/2021 | (“long head of biceps”:ti,ab OR LHB:ti,ab OR “biceps brachii”:ti,ab OR “biceps sling”:ti,ab OR “biceps pulley”:ti,ab OR “superior labrum anterior and posterior”:ti,ab OR SLAP:ti,ab OR (biceps:ti,ab AND tendin*:ti,ab) OR (biceps:ti,ab AND sublux*:ti,ab) OR (biceps:ti,ab AND tear:ti,ab) OR (biceps:ti,ab AND pain:ti,ab)) AND (role:ti,ab OR function:ti,ab OR dysfunction:ti,ab OR EMG:ti,ab OR electromyography:ti,ab OR anatomy:ti,ab OR compensat*:ti,ab OR substitut*:ti,ab OR adapt*:ti,ab OR overactiv*:ti,ab OR hyperactiv*:ti,ab OR underactiv*:ti,ab OR hypoactiv*:ti,ab OR diagnosis:ti,ab OR pathology:ti,ab OR symptomology:ti,ab OR tests:ti,ab OR imaging:ti,ab OR biomechanic*:ti,ab OR arthrokinematics:ti,ab OR proprioception:ti,ab OR kinematics:ti,ab OR “firing patterns”:ti,ab OR “range of motion”:ti,ab OR ROM:ti,ab OR recruit*:ti,ab OR activity:ti,ab OR (muscle:ti,ab AND activation:ti,ab) OR (movement:ti,ab AND patterns:ti,ab) OR elevation:ti,ab OR reach*:ti,ab OR “activities of daily living”:ti,ab OR ADL:ti,ab OR coactivate*:ti,ab OR synergistic:ti,ab OR strength:ti,ab OR weakness:ti,ab OR stability:ti,ab OR depressor:ti,ab OR instability:ti,ab OR stable:ti,ab OR unstable:ti,ab OR stabili*:ti,ab OR sublux*:ti,ab OR dislocate*:ti,ab OR enlocat*:ti,ab OR migrat*:ti,ab OR translat*:ti,ab OR ultrasound:ti,ab OR US:ti,ab OR “diagnostic ultrasound”:ti,ab OR DUS:ti,ab OR “magnetic resonance imaging”:ti,ab OR MRI:ti,ab OR MR:ti,ab OR MRA:ti,ab OR arthrogram:ti,ab OR arthroscopy:ti,ab OR “orthopaedic special tests”:ti,ab OR OST:ti,ab OR test*:ti,ab OR assess*:ti,ab OR exam*:ti,ab OR physiology:ti,ab OR pathophysiology:ti,ab) AND (shoulder:ti,ab OR GHJ:ti,ab OR GHJ:ti,ab OR “HOH”:ti,ab OR “head of humerus”:ti,ab OR HOH:ti,ab OR “proximal humeral”:ti,ab OR “RC”:ti,ab OR “RC interval”:ti,ab OR “RC cable”:ti,ab OR supraspinatus:ti,ab OR infraspinatus:ti,ab OR subscapularis:ti,ab OR “teres minor”:ti,ab OR deltoid:ti,ab) AND (management:ti,ab OR treatment:ti,ab OR intervention:ti,ab OR “evidence based practice”:ti,ab OR surg*:ti,ab OR non-surg*:ti,ab OR operat*:ti,ab OR post-operative:ti,ab OR post-surgical:ti,ab OR procedure:ti,ab OR physiotherapy:ti,ab OR “physical therapy”:ti,ab OR therapy:ti,ab OR rehab*:ti,ab OR conservative:ti,ab OR tenodesis:ti,ab OR tenotomy:ti,ab OR orthopaedic*:ti,ab) | 227 | <https://www.cochranelibrary.com/search> |
| Scopus (basic search) | 07/08/2021 | (“long head of biceps” OR LHB OR “biceps brachii” OR “biceps sling” OR “biceps pulley” OR “superior labrum anterior and posterior” OR SLAP OR (biceps AND tendin*) OR (biceps AND sublux*) OR (biceps AND tear) OR (biceps AND pain)) AND (role OR function OR dysfunction OR EMG OR electromyography OR anatomy OR compensat* OR substitut* OR adapt* OR overactiv* OR hyperactiv* OR underactiv* OR hypoactiv* OR diagnosis OR pathology OR symptomology OR tests OR imaging OR biomechanic* OR arthrokinematics OR proprioception OR kinematics OR “firing patterns” OR “range of motion” OR ROM OR recruit* OR activity OR (muscle AND activation) OR (movement AND patterns) OR elevation OR reach* OR “activities of daily living” OR ADL OR coactivate* OR synergistic OR strength OR weakness OR stability OR depressor OR instability OR stable OR unstable OR stabili* OR sublux* OR dislocate* OR enlocat* OR migrat* OR translat* OR ultrasound OR US OR “diagnostic ultrasound” OR DUS OR “magnetic resonance imaging” OR MRI OR MR OR MRA OR arthrogram OR arthroscopy OR “orthopaedic special tests” OR OST OR test* OR assess* OR exam* OR physiology OR pathophysiology OR (Electromyography OR Physiology OR “Physiopathology” OR Anatomy OR “Magnetic Resonance Imaging” OR “Diagnosis, Differential” OR “Physical Examination” OR Diagnosis OR “Diagnosis” OR “Diagnostic Imaging” OR ultrasonography OR ultrasonics) AND (shoulder OR GHJ OR GHJ OR “HOH” OR “head of humerus” OR HOH OR “proximal humeral” OR “RC” OR “RC interval” OR “RC cable” OR supraspinatus OR infraspinatus OR subscapularis OR “teres minor” OR deltoid OR Shoulder OR “Shoulder Joint”) AND (management OR treatment OR intervention OR “evidence based practice” OR surg* OR non-surg* OR operat* OR post-operative OR post-surgical OR procedure OR physiotherapy OR “physical therapy” OR therapy OR rehab* OR conservative OR tenodesis OR tenotomy OR orthopaedic* OR “Evidence-Based Practice” OR “Physical Therapy Modalities” OR Orthopedics OR “Surgical Procedures, Operative” OR “General Surgery” OR “Treatment Outcome” OR “Orthopedic Procedures” OR “Exercise Therapy” OR “Conservative Treatment” OR Tenodesis OR Tenotomy OR Arthroscopy)) |  |  |
| Scopus (advanced search) | 16/09/2021 | (TITLE-ABS(“long head of biceps”) OR TITLE-ABS(LHB) OR TITLE-ABS(“biceps brachii”) OR TITLE-ABS(“biceps sling”) OR TITLE-ABS(“biceps pulley”) OR TITLE-ABS(“superior labrum anterior and posterior”) OR TITLE-ABS(SLAP) OR (TITLE-ABS(biceps) AND TITLE-ABS(tendin*)) OR (TITLE-ABS(biceps) AND TITLE-ABS(sublux*)) OR (TITLE-ABS(biceps) AND TITLE-ABS(tear)) OR (TITLE-ABS(biceps) AND TITLE-ABS(pain))) AND (TITLE-ABS(role) OR TITLE-ABS(function) OR TITLE-ABS(dysfunction) OR TITLE-ABS(EMG) OR TITLE-ABS(electromyography) OR TITLE-ABS(anatomy) OR TITLE-ABS(compensat*) OR TITLE-ABS(substitut*) OR TITLE-ABS(adapt*) OR TITLE-ABS(overactiv*) OR TITLE-ABS(hyperactiv*) OR TITLE-ABS(underactiv*) OR TITLE-ABS(hypoactiv*) OR TITLE-ABS(diagnosis) OR TITLE-ABS(pathology) OR TITLE-ABS(symptomology) OR TITLE-ABS(tests) OR TITLE-ABS(imaging) OR TITLE-ABS(biomechanic*) OR TITLE-ABS(arthrokinematics) OR TITLE-ABS(proprioception) OR TITLE-ABS(kinematics) OR TITLE-ABS(“firing patterns”) OR TITLE-ABS(“range of motion”) OR TITLE-ABS(ROM) OR TITLE-ABS(recruit*) OR TITLE-ABS(activity) OR (TITLE-ABS(muscle) AND TITLE-ABS(activation)) OR (TITLE-ABS(movement) AND TITLE-ABS(patterns)) OR TITLE-ABS(elevation) OR TITLE-ABS(reach*) OR TITLE-ABS(“activities of daily living”) OR TITLE-ABS(ADL) OR TITLE-ABS(coactivate*) OR TITLE-ABS(synergistic) OR TITLE-ABS(strength) OR TITLE-ABS(weakness) OR TITLE-ABS(stability) OR TITLE-ABS(depressor) OR TITLE-ABS(instability) OR TITLE-ABS(stable) OR TITLE-ABS(unstable) OR TITLE-ABS(stabili*) OR TITLE-ABS(sublux*) OR TITLE-ABS(dislocate*) OR TITLE-ABS(enlocat*) OR TITLE-ABS(migrat*) OR TITLE-ABS(translat*) OR TITLE-ABS(ultrasound) OR TITLE-ABS(US) OR TITLE-ABS(“diagnostic ultrasound”) OR TITLE-ABS(DUS) OR TITLE-ABS(“magnetic resonance imaging”) OR TITLE-ABS(MRI) OR TITLE-ABS(MR) OR TITLE-ABS(MRA) OR TITLE-ABS(arthrogram) OR TITLE-ABS(arthroscopy) OR TITLE-ABS(“orthopaedic special tests”) OR TITLE-ABS(OST) OR TITLE-ABS(test*) OR TITLE-ABS(assess*) OR TITLE-ABS(exam*) OR TITLE-ABS(physiology) OR TITLE-ABS(pathophysiology) OR (INDEXTERMS(Electromyography) OR INDEXTERMS(Physiology) OR “Physiopathology” OR INDEXTERMS(Anatomy) OR INDEXTERMS(“Magnetic Resonance Imaging”) OR INDEXTERMS(“Diagnosis, Differential”) OR INDEXTERMS(“Physical Examination”) OR INDEXTERMS(Diagnosis) OR “Diagnosis” OR “Diagnostic Imaging” OR INDEXTERMS(ultrasonography) OR INDEXTERMS(ultrasonics)) AND (TITLE-ABS(shoulder) OR TITLE-ABS(GHJ) OR TITLE-ABS(GHJ) OR TITLE-ABS(“HOH”) OR TITLE-ABS(“head of humerus”) OR TITLE-ABS(HOH) OR TITLE-ABS(“proximal humeral”) OR TITLE-ABS(“RC”) OR TITLE-ABS(“RC interval”) OR TITLE-ABS(“RC cable”) OR TITLE-ABS(supraspinatus) OR TITLE-ABS(infraspinatus) OR TITLE-ABS(subscapularis) OR TITLE-ABS(“teres minor”) OR TITLE-ABS(deltoid) OR INDEXTERMS(Shoulder) OR INDEXTERMS(“Shoulder Joint”)) AND (TITLE-ABS(management) OR TITLE-ABS(treatment) OR TITLE-ABS(intervention) OR TITLE-ABS(“evidence based practice”) OR TITLE-ABS(surg*) OR TITLE-ABS(non-surg*) OR TITLE-ABS(operat*) OR TITLE-ABS(post-operative) OR TITLE-ABS(post-surgical) OR TITLE-ABS(procedure) OR TITLE-ABS(physiotherapy) OR TITLE-ABS(“physical therapy”) OR TITLE-ABS(therapy) OR TITLE-ABS(rehab*) OR TITLE-ABS(conservative) OR TITLE-ABS(tenodesis) OR TITLE-ABS(tenotomy) OR TITLE-ABS(orthopaedic*) OR INDEXTERMS(“Evidence-Based Practice”) OR INDEXTERMS(“Physical Therapy Modalities”) OR INDEXTERMS(Orthopedics) OR INDEXTERMS(“Surgical Procedures, Operative”) OR INDEXTERMS(“General Surgery”) OR INDEXTERMS(“Treatment Outcome”) OR INDEXTERMS(“Orthopedic Procedures”) OR INDEXTERMS(“Exercise Therapy”) OR INDEXTERMS(“Conservative Treatment”) OR INDEXTERMS(Tenodesis) OR INDEXTERMS(Tenotomy) OR INDEXTERMS(Arthroscopy))) |  |  |
| WOS | 16/09/2021 | (“long head of biceps” OR LHB OR “biceps brachii” OR “biceps sling” OR “biceps pulley” OR “superior labrum anterior and posterior” OR SLAP OR (biceps AND tendin*) OR (biceps AND sublux*) OR (biceps AND tear) OR (biceps AND pain)) AND (role OR function OR dysfunction OR EMG OR electromyography OR anatomy OR compensat* OR substitut* OR adapt* OR overactiv* OR hyperactiv* OR underactiv* OR hypoactiv* OR diagnosis OR pathology OR symptomology OR tests OR imaging OR biomechanic* OR arthrokinematics OR proprioception OR kinematics OR “firing patterns” OR “range of motion” OR ROM OR recruit* OR activity OR (muscle AND activation) OR (movement AND patterns) OR elevation OR reach* OR “activities of daily living” OR ADL OR coactivate* OR synergistic OR strength OR weakness OR stability OR depressor OR instability OR stable OR unstable OR stabili* OR sublux* OR dislocate* OR enlocat* OR migrat* OR translat* OR ultrasound OR US OR “diagnostic ultrasound” OR DUS OR “magnetic resonance imaging” OR MRI OR MR OR MRA OR arthrogram OR arthroscopy OR “orthopaedic special tests” OR OST OR test* OR assess* OR exam* OR physiology OR pathophysiology OR (Electromyography OR Physiology OR “Physiopathology” OR Anatomy OR “Magnetic Resonance Imaging” OR “Diagnosis, Differential” OR “Physical Examination” OR Diagnosis OR “Diagnosis” OR “Diagnostic Imaging” OR ultrasonography OR ultrasonics) AND (shoulder OR GHJ OR GHJ OR “HOH” OR “head of humerus” OR HOH OR “proximal humeral” OR “RC” OR “RC interval” OR “RC cable” OR supraspinatus OR infraspinatus OR subscapularis OR “teres minor” OR deltoid OR Shoulder OR “Shoulder Joint”) AND (management OR treatment OR intervention OR “evidence based practice” OR surg* OR non-surg* OR operat* OR post-operative OR post-surgical OR procedure OR physiotherapy OR “physical therapy” OR therapy OR rehab* OR conservative OR tenodesis OR tenotomy OR orthopaedic* OR “Evidence-Based Practice” OR “Physical Therapy Modalities” OR Orthopedics OR “Surgical Procedures, Operative” OR “General Surgery” OR “Treatment Outcome” OR “Orthopedic Procedures” OR “Exercise Therapy” OR “Conservative Treatment” OR Tenodesis OR Tenotomy OR Arthroscopy)) | 414  Limited by title | <https://www-webofscience-com.ezproxy.bond.edu.au/wos/woscc/summary/e064f2a3-238f-477f-a7fe-493a8807302c-0897af63/relevance/1> |

## References

1. Elser F, Braun S, Dewing CB, Giphart JE, Millett PJ. Anatomy, function, injuries, and treatment of the long head of the biceps brachii tendon. Arthroscopy. 2011;27(4):581-92.

2. Chalmers PN, Cip J, Trombley R, Cole BJ, Wimmer MA, Romeo AA, et al. Glenohumeral Function of the Long Head of the Biceps Muscle: An Electromyographic Analysis. Orthopaedic journal of sports medicine. 2014;2(2):2325967114523902.

3. Sarmento M. Long head of biceps: from anatomy to treatment. Acta reumatologica portuguesa. 2015;40(1):26-33.

4. Warner JJ, McMahon PJ. The role of the long head of the biceps brachii in superior stability of the glenohumeral joint. The Journal of bone and joint surgery American volume. 1995;77(3):366-72.
