# Supplementary material for: The long head of biceps at the shoulder: a scoping review
Source: BMC Musculoskelet Disord. 2023 Mar 28;24:232. doi: 10.1186/s12891-023-06346-5 (PMC10044783; doi:10.1186/s12891-023-06346-5)
Supplement: Supplementary file 4 — Supplementary Material 4 [file 12891_2023_6346_MOESM4_ESM.docx]

# Additional file 4: Supplementary Table 2_BMC.docx; Anatomical variations of the Biceps Brachii

| Author | LOE | Symptoms/Pathology | Intervention | Anatomical variation (No) | Associated pathology |
| --- | --- | --- | --- | --- | --- |
| Ayoubi et al. (2021) | IV | Case study - 51M Sh pain. | Incidental arthroscopy (n=1) | Aberrant origin (n=1) | LHBT origin located inside bifid SSC tendon. LHBT partial thickness tear and tendinopathy. |
| Banerjee and Patel (2015) | IV | Case study - 49F Sh pain. | Incidental arthroscopy (n=1) | Aberrant origin (n=1) | Bifurcated origin of the LHBT from the undersurface of the SSP tendon. |
| Carroll et al. (2019) | IV | NA. | Cadaver dissection (n=1) | Aberrant origin (n=1), 3H biceps (n=1) | Bilateral supernumerary 3rd head of the BB from the posterior surface of the PM tendon with chondroepitrochlearis. |
| Cutler et al. (2018) | IV | Case study - 42M Sh pain.  MRI - PTT SSP. | Incidental arthroscopy (n=1) | Aberrant origin (n=1) | Trifurcate origin of the LHBT from the a) SSP tendon, b) superior labrum, and c) RC interval. |
| El Abiad et al. (2019) | IV | Case study - 25M Sh pain. | Incidental MRI (n=1) | Absent LHBT (n=1) | Absent LHBT and bicipital groove associated with a large insertion of the SSC muscle and SSP tear. |
| Enad (2004) | IV | Case study 1 - 35M Sh pain.  Case study 1 - 46M Sh pain. | Diagnostic arthroscopy (n=90) | Aberrant origin (n=2) | Bifurcate LHBT origin from the a) supraglenoid tubercle and b) posterosuperior capsulolabral.  *Prevalence of bifurcate origin = 2.2% |
| Enix et al. (2021) | V | NA. | Cadaver dissection (n=1) | 3H biceps (n=1) | Tricipital supernumerary head of the BB muscle originating from the lateral portion of the proximal 3rd of the humerus. |
| Franco et al. (2005) | IV | Case study - 37M Sh weakness and instability. MRI - anterior labral tear. | Incidental arthroscopy (n=1) | Absent LHBT (n=1) | Absence of the LHBT, with attenuated anterior structures and widened RC interval. |
| Fraser et al. (2015) | IV | NA. | Cadaver dissection (n=1) | 3H biceps (n=1) | SHB tendon origin from a) coracoid process, b) inferolateral surface of the PM muscle. LHBT origin from the supraglenoid tubercle. |
| Ghalayini et al. (2007) | IV | Case study 1 - 37F Sh instability.  Case study 2 - 28F Sh instability.  Case study 3 - 34F Sh pain and stiffness. | Incidental arthroscopy (n=3) | Absent LHBT (n=3) | Case study 1 - MRI - absence of the LHBT and biceps groove.  Case study 2 - MRI - subacromial impingement.  Case study 3 - Initial MRI - SSP tendon tear. |
| Gheno et al. (2010) | V | NA | Cadaver MRI (n=10) | 3H biceps (n=2) | Two origins from the greater tuberosity near the articular capsule, intimate with the LHBT in the bicipital groove.  *Prevalence of accessory head of BB = 20%. |
| Gillardin et al. (2013) | IV | Case study - 22M absent LHBT on DUS. | Diagnostic MRA (n=1) | Absence LHBT (n=1) | Shallow biceps groove. |
| Glueck et al. (2003) | IV | Case study - 25F Sh pain and instability. | Diagnostic arthroscopy (n=1) | Absence LHBT (n=1) | Congenital absence of the LHBT. Absent RC interval foramen for the LHBT. |
| Jeung Yeol et al. (2017) | III | NA. | Arthroscopic images and medical records of (n=660) | Aberrant origin of LHB (n=43):   - 67.4% mesotenon - 32.6% adherent - 0% split - 0% ABS | RC tear n=23 (53.5%), superior labral tear n=11 (25.6%), adhesive capsulitis n=8 (18.6%), anterior instability n=8 (18.6%).  Sh joint instability was more prevalent in the mesotenon group vs. normal group. Not significant (27.6%: 14.9%, p= 0.305).  *Overall prevalence of anatomical variation of LHB = 6.5%. |
| Kim et al. (2007) | IV | Case study - 27F Sh pain, instability. MRI – ISP tear. | Incidental arthroscopy (n=1) | Aberrant origin (n=1) | Bifurcated LHBT 1cm distal to its tendon origin from the supraglenoid tubercle. |
| Koplas et al. (2009) | IV | Case study - 40M Sh pain. MRI - absence LHBT with shallow biceps groove. FTT SSP and superior labrum tear. | Diagnostic arthroscopy (n=1) | Absent LHBT (n=2) | Bilateral congenital absence of the LHBT. |
| Kosugi et al. (1992) | V | NA. | 546 Sh cadaver dissections (n=273) | 3H biceps (n=34)  4H biceps (n=7) | 3-headed BB - Origin varied from a) shaft of the humerus between coracobrachialis and brachialis muscles (n=39), b) shaft of the humerus and medial brachial intermuscular septum (n=27), c) shaft of the humerus and other sites (n=3), d) medial brachial intermuscular septum (n=2) and e) other, including the PM or the deltoid, greater tubercle, or articular capsule (n=4).  4-headed BB - Origin varied from a) shaft of humerus (n=2), b) tendon of PM (n = 3), c) greater tubercle (n=1), and d) articular capsule (n=1). The presence of a supernumerary head affected the course and branching of the musculocutaneous nerve.  *Prevalence of supernumerary heads of the BB = 13.7% |
| Kumar et al. (2017) | IV | Systematic review: Sh pain (85.7%), Sh instability (37.1%). *Cadaveric studies excluded. | Arthroscopy studies reporting the absence of  LHBT | Absent LHBT (n=35) | LHBT is the most common variant and is missed in 60% of the cases on the initial MRI.  *Prevalence: ABS and the presence of Sh pain (85.7%) and instability (37.1%) |
| Mariani et al. (1997) | IV | Case study - 23M Sh pain. MRI - hyperintensity SSP tendon. | Incidental arthroscopy (n=1) | Absent LHBT (n=1) | Nil other. |
| Nasr and Hussein (2013) | V | NA. | 100 Sh cadaver dissections (n=50) | Anatomical variation (n=10):   - Aberrant origin (n=9): - 3H (n=7) - 4H (n=2) - Aberrant insertion (n=1) | 3H BB - Origin varied from a) anteromedial surface of the humerus between the coracobrachialis insertion and the origin of brachialis muscle (60%), b) middle of the medial border of the humerus (30%), c) supernumerary heads inserted into the common tendon of BB muscle in (70%), d) LHBT received the extra-heads in 2 (20%) cases and the supernumerary heads united with SHB muscle in n=1 (10%) limb only.  4H BB - Origin varied from a) GHJ capsule (1%) and b) coracoid process of scapular (1%). The biceps common tendon insertion received the supernumerary heads in 7% of the limbs. However, the extra-head fused with the long head in 2 (2%) limbs and united with the short head in 1 (1%) limb.  *Overall prevalence of anatomical variation of BB = 10% |
| Pandey et al. (2014) | IV | Case study - 36M Sh pain. DUS - SSP tendinopathy with dynamic impingement, thickened subacromial-subdeltoid bursa. | Incidental arthroscopy (n=1) | Aberrant origin (n=1) | Bifurcated LHBT with one origin from a) superior labrum and b) posterosuperior capsule. The intraarticular portion of LHBT is adherent to the undersurface of the SSP tendon. |
| Rao et al. (2003) | II | Case series – Sh pain. | Diagnostic arthroscopy (n=546) | Aberrant origin (n=73) | Anterosuperior labrum variations a) sublabral foramen in n=18 (3.3%), b) sublabral foramen with a cord-like middle GHL in n=47 (8.6%), c) absence of labral tissue at the anterosuperior portion of the labrum with a cord-like middle GHL in n=8 (1.5%).  Three independent variables strongly associated with anterosuperior labral variations: anterosuperior labral variation: a) anterosuperior labral fraying (odds ratio, 3.58; p=0.000), b) abnormal superior GHL (odds ratio, 3.69; p=0.012) and c) Passive IR in 90deg of Sh Abd (odds ratio, 1.13; p=0.046).  *Prevalence of anterosuperior labrum variations = 13.4%. |
| Refior and Sowa (1995) | V | NA. | 104 Sh cadaver dissections (n=52) | Aberrant origin (n=104) | 30% of Sh LHBT originated from the supraglenoid tubercle, and 45% of Sh LHBT originated in a Y-form directly from the glenoid labrum with fibres visible from both the ventral and the dorsal aspects of the labrum. 25% of the Sh LHBT originated from the supraglenoid tubercle and the labrum.  *Prevalence of aberrant origins = 70%. |
| Sabzevari et al. (2019) | IV | Case series - Sh pain. | Diagnostic arthroscopy (n=7) | Aberrant origin (n=7) | Anomalous origins of the LHBT from a) medial insertion of LHBT to the superior labrum (n=2), b) proximal insertion of LHBT to the SSP confluent with the superior labrum (n=2), c) bifid LHBT (n=1) and d) insertion of LHBT into the superior capsular tissues at the RC interval, just proximal to its insertion site on the superior labrum (n=2). |
| Sayeed et al. (2008) | IV | Case study - 18M Sh pain and instability. CT - marked glenoid dysplasia. MRA - absent LHBT with shallow biceps groove and large posterior labral tear. | Diagnostic arthroscopy (n=1) | Absent LHBT (n=1) | Congenital absence of the LHBT. Absent rotator interval foramen for the LHBT.  Associated pathology - Complex labral tear partially detached from the posterior glenoid. |
| Schoenleber and Spinner (2006) | V | NA. | Cadaver dissection (n=1) | 3H biceps (n=1) | 3H BB - Origin from the tendinous aspect of the deltoid muscle, running distally as a narrow band converging with the LHB and SHB to form the common tendon insertion. |
| Smith et al. (2002) | IV | Case study - 16M recurrent Sh dislocation. | Arthroscopic evaluation (n=1) | Absent LHBT (n=1) | Congenital absence of LHBT with a significant hypoplastic superior labrum, redundant inferior capsular pouch and attenuated and redundant anterior, middle, and inferior GHL. |
| Traverso et al. (2020) | IV | Case study - 18M Sh pain. MRI - LHBT rupture. | Diagnostic MRI (n=1) | Absent LHBT (n=1) | Repeat MRI - agenesis of LHBT. |
| Vangsness Jr et al. (1994) | V | NA. | Cadaver dissections (n=100) | Aberrant origin (n=50) | LHBT origin - Approx. 50% of specimens attached to the supraglenoid tubercle and 50% from the superior glenoid labrum. Main labral origins; a) type 1 (22%) - all the labral attachment to the posterior labrum, with none to the anterior labrum, b) type 2 (33%) - most of the labral attachment to the posterior labrum, with a small contribution to the anterior labrum, c) type 3 (37%) - equal contributions of the labral attachment to the anterior and posterior labrum, d) type 4 (8%) - most of the labral attachment to the anterior labrum, with a small contribution to the posterior labrum. |
| Vijayabhaskar et al. (2008) | V | NA. | Cadaver dissection (n=1) | 3H biceps (n=1) | Unilateral 3H BB - Origin from tendon of deltoid. |
| Wade et al. (2020) | IV | Case study - 30M Sh pain and instability. MRI - PTT SSP tendon and fraying of the anteroinferior glenoid labrum. | Incidental arthroscopy (n=1) | Aberrant origin (n=1) | Anomalous LHBT origin from the inferior surface of the SSP muscle outside the capsule. |
| Williams et al. (1994) | IV | Case series - Sh pain. | Arthroscopy videotapes (n=200) | Aberrant origin (n=27) | Twenty-four patients (12%) - sublabral foramen below the anterosuperior labrum.  Eighteen patients (9%) - cord-like Middle GHL attached directly to the labrum near the sublabral foramen, superior to anterior glenoid notch.  3 patients (1.5%) - Buford complex.  *Prevalence anatomic labral variations = 13.5% |
| Winston et al. (2017) | IV | Case study - 18M Sh instability. MRI - anterior Bankart & Hill-Sachs lesion. Atypical non-visualisation of LHBT and absence of bicipital groove. | Diagnostic arthroscopy (n=1) | Absent LHBT (n=1) | Nil other. |
| Wittstein et al. (2012) | IV | Case study 1 - 42M Sh pain.  Case study 2 - 38F Sh pain. | Incidental arthroscopy (n=2) | Aberrant origin (n=2) | Case 1 - bifurcate LHBT with Y shape origin from the a) rotator cable and b) scapular neck medial to the supraglenoid. Associated pathology *Under surface tearing of SSP.  Case 2 - bifurcate LHBT with Y -shape origin from the a) rotator cable and b) scapular neck medial to the superior glenoid. |
| Yershov and Hudák (2015) | IV | NA. | Cadaver dissection (n=1) | 3H biceps (n=1) | 3H BB - Origin from coracoid process origin with SHB. The third head split from the SHB with three different humeral insertions enfolding the median nerve and the brachial artery. |
| Zhang et al. (2014) | IV | Case study 1 - 26M Sh pain. MRI - PASTA lesion and aberrant origin of the LHBT.  Case study 2 - 39M Sh pain. MRA - PASTA lesion and aberrant origin of the LHBT. | Incidental MRI/Arthroscopy (n=2) | Aberrant origin (n=2) | Case 1 - LHBT origin from the anterior edge of the SSP tendon insertion and the area of the PASTA lesion.  Case 2 - LHBT origin from SSP tendon at its anterior edge with associated PASTA lesion. |

List of Abbreviations: Abduction (Abd); Absence of LHB (ABS); Biceps Brachii (BB); Computerised Tomography (CT); Diagnostic Ultrasound (DUS); Four Headed (4H); Full Thickness Tear (FTT); Glenohumeral Ligament (GHL); Infraspinatus (ISP); Level of Evidence (LOE); Long Head of Biceps (LHB); Long Head of Biceps Tendon (LHBT); Magnetic Resonance Arthrography (MRA); Magnetic Resonance Imaging (MRI); Partial Articular-sided Supraspinatus Tendon Avulsion (PASTA); Partial Thickness Tear (PTT); Pectoralis Major (PM); Rotator Cuff (RC); Short Head of Biceps (SHB); Shoulder (Sh); Subscapularis (SSC); Supraspinatus (SSP); Three Headed (3H).

References

1. Ayoubi R, Darwish M, Nassour N, Aouad D, Maalouly J, Kanj V, et al. A rare case of an anatomical variation of bilateral long head of the biceps brachii tendons identified within the substance of bifid subscapularis tendons. Asia Pac J Sports Med Arthrosc Rehabil Technol. 2021;23:18-21.

2. Banerjee S, Patel VR. Anomalous biceps origin from the rotator cuff. Indian J Orthop. 2015;49(1):105-8.

3. Carroll MA, Lebron EM, Jensen TE, Cooperman TJ. Chondroepitrochlearis and a supernumerary head of the biceps brachii. Anat Sci Int. 2019;94(4):330-4.

4. Cutler HS, Tao MA, O'Brien SJ, Taylor SA. Trifurcate Origin of Long Head of Biceps Brachii: A Case Report and Literature Review. J Orthop Case Rep. 2018;8(4):70-3.

5. El Abiad JM, Faddoul DG, Baydoun H. Case report: Broad insertion of a large subscapularis tendon in association with congenital absence of the long head of the biceps tendon. Skeletal Radiol. 2019;48(1):159-62.

6. Enad JG. Bifurcate origin of the long head of the biceps tendon. Arthroscopy. 2004;20(10):1081-3.

7. Enix D, Scali F, Sudkamp K, Keating R. Supernumerary Head of the Biceps Brachii Muscle: An Anatomic Variant With Clinical Implications. J Chiropr Med. 2021;20(1):37-42.

8. Franco JC, Knapp TP, Mandelbaum BR. Congenital absence of the long head of the biceps tendon. A case report. J Bone Joint Surg Am. 2005;87(7):1584-6.

9. Fraser PR, Howard LW, Rosales AA, Guttmann GD. Bilateral symmetrical supernumerary heads of biceps brachii with rare pectoralis major insertion. Surg Radiol Anat. 2015;37(3):299-302.

10. Ghalayini SR, Board TN, Srinivasan MS. Anatomic variations in the long head of biceps: contribution to shoulder dysfunction. Arthroscopy. 2007;23(9):1012-8.

11. Gheno R, Zoner CS, Buck FM, Nico MA, Haghighi P, Trudell DJ, et al. Accessory head of biceps brachii muscle: anatomy, histology, and MRI in cadavers. AJR Am J Roentgenol. 2010;194(1):W80-3.

12. Gillardin P, Vanhoenacker FM, Wauters T, De Backer AI. Congenital absence of long head of the biceps tendon. JBR-BTR. 2013;96(5):320.

13. Glueck DA, Mair SD, Johnson DL. Shoulder instability with absence of the long head of the biceps tendon. Arthroscopy. 2003;19(7):787-9.

14. Jeong JY, Park SM, Park YE, Yoo JC. Morphological classification of anatomical variants of the intra-articular portion of the long head of the biceps brachii tendon and analysis of the incidence and the relationship with shoulder disease for each subtype. J Orthop Surg (Hong Kong). 2017;25(3):2309499017742207.

15. Kim KC, Rhee KJ, Shin HD, Kim YM. Biceps long head tendon revisited: a case report of split tendon arising from single origin. Arch Orthop Trauma Surg. 2008;128(5):495-8.

16. Koplas MC, Winalski CS, Ulmer WH, Jr., Recht M. Bilateral congenital absence of the long head of the biceps tendon. Skeletal Radiol. 2009;38(7):715-9.

17. Kosugi K, Shibata S, Yamashita H. Supernumerary head of biceps brachii and branching pattern of the musculocutaneus nerve in Japanese. Surg Radiol Anat. 1992;14(2):175-85.

18. Kumar CD, Rakesh J, Tungish B, Singh DM. Congenital absence of the long head of biceps tendon & its clinical implications: a systematic review of the literature. Muscles Ligaments Tendons J. 2017;7(3):562-9.

19. Mariani PP, Bellelli A, Botticella C. Arthroscopic absence of the long head of the biceps tendon. Arthroscopy. 1997;13(4):499-501.

20. Nasr AY, Hussein AM. Morphology and clinical implication of the extra-head of biceps brachii muscle. Folia Morphol (Warsz). 2013;72(4):349-56.

21. Pandey V, van Laarhoven SN, Arora G, Rao S. Bifurcated intraarticular long head of biceps tendon. Indian J Orthop. 2014;48(4):432-4.

22. Rao AG, Kim TK, Chronopoulos E, McFarland EG. Anatomical Variants in the Anterosuperior Aspect of the Glenoid Labrum. Journal of Bone & Joint Surgery, American Volume. 2003;85(4):653.

23. Refior HJ, Sowa D. Long tendon of the biceps brachii: sites of predilection for degenerative lesions. J Shoulder Elbow Surg. 1995;4(6):436-40.

24. Sabzevari S, Khalilipour Roudi M, Kalawadia J, Lin A. Chronic Long Head Biceps Tendinitis Secondary to Anomalous Origins in Young Patients: A Case Series. Arch Bone Jt Surg. 2019;7(6):493-7.

25. Sayeed SA, Shah JP, Collins MS, Dahm DL. Absence of the long head of the biceps tendon associated with glenoid dysplasia and posterior labral tear. Clin Anat. 2008;21(7):728-32.

26. Schoenleber SJ, Spinner RJ. An unusual variant of the biceps brachii. Clin Anat. 2006;19(8):702-3.

27. Smith EL, Matzkin EG, Kim DH, Harpstrite JK, Kan DM. Congenital absence of the long head of the biceps brachii tendon as a VATER association. Am J Orthop (Belle Mead NJ). 2002;31(8):452-4.

28. Traverso A, Piasecki K, Gallusser N, Farron A. Agenesis of the long head of the biceps brachii tendon: ignored variations of the anatomy and the next tendon to disappear? BMJ Case Rep. 2020;13(5).

29. Vangsness CT, Jorgenson SS, Watson T, Johnson DL. The origin of the long head of the biceps from the scapula and glenoid labrum. An anatomical study of 100 shoulders. The Journal of Bone and Joint Surgery British volume. 1994;76-B(6):951-4.

30. Vijayabhaskar P, Baral P, Vaishya R, Shrestha RN. Supernumerary head of Biceps brachii: a rare occurrence in the Nepalese population. Kathmandu Univ Med J (KUMJ). 2008;6(2):225-7.

31. Wade R, Shah SS, B SS, Shah KA, Raj A. A Rare Anatomical Variation in the Origin of the Tendon of Long Head of Biceps in a 30-Year-Old Male: A Case Report and Review of Literature. J Orthop Case Rep. 2020;9(6):94-7.

32. Williams MM, Snyder SJ, Buford D, Jr. The Buford complex--the "cord-like" middle glenohumeral ligament and absent anterosuperior labrum complex: a normal anatomic capsulolabral variant. Arthroscopy. 1994;10(3):241-7.

33. Winston BA, Robinson K, Crawford D. "Monocept": A Brief Report of Congenital Absence of the Long Head of the Biceps Tendon and Literature Review. Case Rep Orthop. 2017;2017:1090245.

34. Wittstein J, Lassiter T, Jr., Taylor D. Aberrant origin of the long head of the biceps: a case series. J Shoulder Elbow Surg. 2012;21(3):356-60.

35. Yershov D, Hudak R. Unusual Variation of the Biceps Brachii with Possible Median Nerve Entrapment. Prague Med Rep. 2015;116(2):167-72.

36. Zhang AL, Gates CH, Link TM, Ma CB. Abnormal origins of the long head of the biceps tendon can lead to rotator cuff pathology: a report of two cases. Skeletal Radiol. 2014;43(11):1621-6.
