# Supplementary material for: The long head of biceps at the shoulder: a scoping review
Source: BMC Musculoskelet Disord. 2023 Mar 28;24:232. doi: 10.1186/s12891-023-06346-5 (PMC10044783; doi:10.1186/s12891-023-06346-5)
Supplement: Supplementary file 5 — Supplementary Material 5 [file 12891_2023_6346_MOESM5_ESM.docx]

| Authors | LOE | No | Population | Results |
| --- | --- | --- | --- | --- |
| Gleason et al. (2006) | IV | 14 | Cadaver + MRI | No identifiable THL fibres covering the intertubercular groove composed of biceps sling formed from the fibres of the SSC tendon, with contributions from the SSP tendon and the coracohumeral ligament. |
| MacDonald et al. (2007) | V | 85 | Cadaver | No THL was identified. In most cases (86%), tendinous fibres from SSC overlaid the LHBT as it emerged from the capsule of the GHJ. |
| Singh et al. (2015) | V | 18 | Cadaver | No separate THL was detected. Tissues covering the bicipital groove formed by tendinous fibres of the SSC or both the SSC and SSP or fibrous expansion of posterior lamina of PM muscle. |

# Additional file 5: Supplementary Table 3_BMC.docx; Transverse Humeral Ligament

List of Abbreviations: Glenohumeral Joint (GHJ); Level of Evidence (LOE); Long Head of Biceps Tendon (LHBT); Magnetic Resonance Imaging (MRI); P-value (p); Pectoralis Major (PM); Subscapularis (SSC); Supraspinatus (SSP); Transverse Humeral Ligament (THL).

References

1. Gleason PD, Beall DP, Sanders TG, Bond JL, Ly JQ, Holland LL, et al. The transverse humeral ligament: a separate anatomical structure or a continuation of the osseous attachment of the rotator cuff? Am J Sports Med. 2006;34(1):72-7.

2. MacDonald K, Bridger J, Cash C, Parkin I. Transverse humeral ligament: does it exist? Clin Anat. 2007;20(6):663-7.

3. Singh R, Singla M, Tubbs RS. Macro/micro observational studies of fibres maintaining the biceps brachii tendon in the bicipital groove: application to surgery, pathology and kinesiology. Folia Morphol (Warsz). 2015;74(4):439-46.
