# Supplementary material for: The long head of biceps at the shoulder: a scoping review
Source: BMC Musculoskelet Disord. 2023 Mar 28;24:232. doi: 10.1186/s12891-023-06346-5 (PMC10044783; doi:10.1186/s12891-023-06346-5)
Supplement: Supplementary file 6 — Supplementary Material 6 [file 12891_2023_6346_MOESM6_ESM.docx]

# Additional file 6: Supplementary Table 4_BMC.docx; EMG methodology and results findings for LHB

| Author | LOE | No | Participants | Functional task | EMG type | Results | Implications |
| --- | --- | --- | --- | --- | --- | --- | --- |
| Ahamed et al. (2014) | III | 16 | Asymptomatic cricket players | Fast Bowling (FB) and Slow Bowling (SB) | sEMG: (BB). | BB muscle activity: FB > SB.  Maximum BB activity: Ball release > follow-through during both FB and SB. | Supports BB activity in overarm bowling (FB > SB). |
| Baek et al. (2021) | III | 21 | RC tendinopathy vs. unaffected Sh | Predictable and unpredictable ball (200g and 500g) drop/catch. | sEMG (BB, ISP, UT, AD) | Significantly delayed anticipatory muscle activation for ISP, UT, AD and BB between RC tendinopathy Sh vs. unaffected Sh during a predictable and unpredictable ball-drop trial, except for 500g predictable ball drop. | Supports timing delay in BB and RC during a ball drop task in patients with RC tendinopathy. |
| Basmajian and Latif (1957) | III | 20 | Normal young adults | Standing Sh Flex and Abd. | fwEMG: (LHB, SHB, Br, Bc). | LHB muscle activity: Sh Flex in both Sup and Semi-Pro positions of the forearm.  Maximum LHB activity: Isometric Sh Abd when forearm was Sup and Sh ER. | Supports LHB as active Sh Flex and Abd |
| Borms et al. (2017) | IV | 30 | Healthy asymptomatic volunteers | Eleven biceps targeted rehabilitation exercises. | sEMG:  (BB, TB, UT, MT, LT, SA). | Top seven exercises for BB activity from least active to most active (%MVIC):   1. Pull-up in Pro with cord (24.06%) - moderate activity 2. Air punch in Sh Flex 90º (27.58%) - moderate activity 3. Dumbbell Sh Flex in ER and forearm Sup (35.56%) - moderate activity 4. Pull-up in Sup with cord (41.96%) - moderate activity 5. Incline dumbbell biceps curl (43.99%) - moderate activity 6. Underarm throwing Sh Flex in 90º with soft weight ball (56.96%) - high activity 7. Reverse punch with elastic tubing (67.37%) - high activity | Supports high BB activity during unsupported elbow Flex and Sup, in 90 degrees Sh Flex OR during high-velocity explosive exercises such as underarm bowling. |
| Brown et al. (1993) | IV | 15 | Healthy subjects | Rapid Sup/Pro in a) elbow Flex with and without 5kg load and b) Sh IR/ER. | sEMG: (LHB, SHB). | The LHB to SHB activity ratio significantly decreased (p<0.05) as the elbow position varied from 70° to 120°. SHB is more active in elbow Flex at 120° and LHB more active in elbow E at 70°. When the Sh position was changed from IR to ER, the intensity of the LHB in comparison to its MVC increased significantly (p<0.05) in 75% of subjects and the ratio of LHB to SHB activity increased significantly (p<0.05) in 63 % of subjects. | Supports functional differentiation of the LHB and SHB at the Sh and elbow. |
| P. N. Chalmers et al. (2014) | III | 13 | Normal healthy volunteers | Sh Abd and Flex (0° 45°, 90°)  +/- elbow brace to minimise LHB activity at the elbow +/- 1kg weight. | sEMG: (LHB, SHB, MD, ISP, Br). | Significantly increased LHB muscle activity in both Sh Abd and Flex vs. neutral position, regardless of elbow immobilisation or load (p<0.049 in all cases). Biceps loading increased LHB muscle activity in all planes of motion except for the neutral position, regardless of elbow immobilisation. A significant increase in LHB muscle activity was demonstrated in 45° of Abd (p=0.028) and 90° of Flex (p=0.033) regardless of elbow immobilisation. | Supports LHB is active during both Sh Flex and Abd. |
| Cools et al. (2014) | IV | 32 | Healthy asymptomatic volunteers | Sixteen common Sh exercises, including three biceps, targeted rehabilitation exercises. | sEMG:  (UT, MT, LT, SA, AD, PD, BB, TB). | Top five exercises for BB activity from least active to most active (%MVIC):   1. Pulley forearm Sup (15.4%) - low activity 2. Dumbbell uppercut (19.3%) - low activity 3. Dumbbell full can (29.0%) - moderate activity 4. Pulley elbow Flex in forearm Sup (34.6%) - moderate activity 5. Dumbbell Sh Flex in ER and forearm Sup (35.9%) - moderate activity | Supports moderate BB activity during Sh Flex in elbow Ext and Sup OR during resisted elbow Flex in Sup. |
| David et al. (2000) | IV | 15 | Asymptomatic healthy patients | Isokinetic Sh IR/ER with the arm positioned at 45° of Abd in the scapular plane (scaption). | sEMG: (SSP, ISP, SSC, LHB, AD, MD, PD, PM).  fwEMG: (SSP, SSC). | The RC (0.092±0.038s) and LHB (0.215±0.045s) were active before the initiation of movement in all testing conditions (p<0.05). The RC (0.092 ± 0.038s) and LHB (0.215 ± 0.045s) were active prior to onset of activity in prime movers (Deltoid and PM) in all testing conditions (P < 0.05). | Supports the pre-setting role of the LHB and RC during Sh rotation at 45° scaption. |
| Furlani (1976) | IV | 30 | Young adult healthy volunteers | Sh Flex/E, Abd/Add, IR/ER +/- elbow E/FLEX +/- load. | fwEMG: (LHB, SHB). | LHB maximum activity: Sh Flex with elbow E with no load (n=29) > with load (n=30) > Abd (n=3).  LHB activity from most active to least active:   - Sh Flex, elbow E, no resistance (n=13 - strong activity; n=9 - moderate activity; n= 5 - very strong activity and n= 2 - slight activity). - Sh Flex, elbow E, against resistance (n=30 active – no individual grades given). - Sh Abd, elbow E, against resistance (n=3 - Moderate activity). | Supports LHB activity during Sh Flex > Abd greatest in elbow E. |
| Glousman et al. (1988) | III | 15 | Throwing athletes with anterior Sh instability vs healthy controls | Baseball pitching:   - Wind-up - Early cocking - Late cocking - Acceleration - Follow-through | fwEMG:(BB, MD, SSP, ISP, PM, SSC, SA). | Peak BB activity occurred in the late cocking phase for both unstable and normal Sh:   - Normal Sh - Wind-up = 8% (Slight), early cocking = 17% (Slight), late cocking = 26% (Moderate), *acceleration = 12% (Slight) and follow-through =13% (Slight). - Unstable Sh - Wind-up = 8% (Slight), early cocking = 21% (Moderate), late cocking = 35% (Moderate), *acceleration = 32% (Moderate) and follow-through = 18% (Slight). - A significant difference in BB activity during the *acceleration phase between normal and unstable Sh (p<0.05). Increased activity was observed in unstable Sh vs healthy Sh. | Supports increased BB activity during pitching.  Hyperactivity of BB during pitching in subjects with anterior Sh instability.  Potential for BB strengthening in rehab of Sh instability. |
| Hawkes, Alizadehkhaiyat, Fisher, et al. (2012) | IV | 12 | Healthy volunteers  . | Modified FIT-HaNSA (a shelf-lifting task with 1kg weight) | sEMG: (AD, MD, PD, PM, UT, SA, LD, TM, Br, BB).  fwEMG:(SSP, ISP, SSC). | No significant inter-muscular coordination correlations between the activity of the elbow flex (BB and Br) and the RC (PCC = -0.12, p = 0.241) during an Sh elevation task.  *PCC = Pearson Correlation Coefficient | BB - Greater activity in Phase 1 > Phase 2 of Sh elevation task.  No significant inter-muscular coordination correlation between elbow flex and RC during an Sh elevation task. |
| Hawkes, Alizadehkhaiyat, Kemp, et al. (2012) | III | 11 | Subjects with MRCT vs. healthy controls | Modified FIT-HaNSA (a shelf-lifting task with 1kg weight). | sEMG: (AD, MD, PD, PM, UT, SA, LD, TM, Br and BB).  fwEMG: (SSP, ISP, SSC). | EMG activity was significantly higher for the BB - Br (p < 0.001), UT-SA (p = 0.025), muscle groups and for the LD (p = 0.010), and TM (p = 0.007) muscles in patients with MRCT vs controls during a Sh elevation task. | Supports increased BB activity and compensation in patients with MRCT during an Sh elevation task.  Potential for inclusion of BB strengthening in rehab of MRCT. |
| Hawkes et al. (2014) | II | 11 | Subjects with MRCT vs. healthy controls | Submaximal grip task. | sEMG:(AD, MD, PD, PM, UT, SA, LD, TM, Br and BB).  fwEMG: (SSP, ISP and SSC). | No significant fatigue of BB in patients with MRCT (p = 0.120) compared to healthy controls (p = 0.727). No significant group comparisons for BB (p = 0.342) during a gripping task. | Supports no differences in fatigue of BB activity in patients with MRCT vs controls during a gripping task. |
| Jobe et al. (1984) | IV | 4 | Healthy professional baseball pitchers. | Fastball baseball pitching:   - Wind-up - Cocking - Acceleration - Follow-through | fwEMG: (BB, TB, PM, LD, SA, Bc). | BB % MMT (activity) in healthy Sh:   - Wind-up = % not reported - Cocking = 36% (modest activity) - Acceleration = 25% (very low activity) - Follow-through = % not reported (Peak activity) | Supports the role of BB in pitching, with the highest activity occurring during the cocking and follow-through phase. |
| Kido et al. (1998) | III | 37 | Subjects with FTT RC tear vs. controls. | Sh elevation in the scapular plane (scaption) to 120° with and without a 1kg weight. | sEMG: (BB). | RC tear group:   - 14 of 40 (35%) Sh showed a %MVIC of the BB greater than 10% without load, which increased after load application (p = 0.006). Significant increase in %MVC of the biceps as elevation angle increased in both groups (p < 0.0001).   Differences between the RC tear and healthy control were statistically significant at 30deg (p = 0.05), 60deg (p = 0.04), and 90deg (p = 0.01) of elevation with loading.  Sh with increased activity of the BB tended to show less strength in Abd and ER, although not statistically significant (p = 0.06 in Abd, p = 0.08 in ER). | Supports increased BB activity and compensation in patients with FTT RC tear during an Sh elevation task. |
| Kim et al. (2001) | III | 38 | Symptomatic unstable Sh vs. contralateral control. | Static Sh Abd at 0°, 45°, 90°, and 120° with progressive ER to anterior apprehension + *elbow brace to minimise LHB at the elbow. | fwEMG: (LHB and SSP). | LHB - Stable Sh:   - No significant difference in LHB activity in Abd positions.   LHB - Unstable Sh:   - Higher EMG activity of the LHB during ER of the Sh. - Significantly greater LHB activity in unstable Sh vs. contralateral stable Sh in Abd positions (P=0.00). - RMS voltage of the LHB was maximal at 90° and 120° of ER in the unstable Sh (P≤0.05). | Supports increased LHB activity in unstable Sh. Suggests active compensatory  role of LHB in anterior Sh instability. |
| Levy et al. (2001) | IV | 10 | Nondominant healthy Sh  . | Sh Flex, Abd, ER/IR at 90° Abd and E under variable speed and load + *elbow brace to minimise LHB at the elbow. | sEMG: (Br, AD)  fwEMG: (SSP, ISP, LHB, Bc). | No significant LHB activity occurred during variable speeds or loads with the elbow immobilised in a brace for all active Sh movements. | Minimal LHB activity during Sh ROM with the elbow immobilised in a brace. |
| Lisowski et al. (2014) | III | 15 | Healthy individuals, experimental vs control group  . | Active Sh Flex, Scaption, ER/IR at 90° Abd before and after an active elbow flexor fatigue protocol. | sEMG: (AD, MD, PD, BB, ISP). | No significant change in BB muscle activity pre and post-BB fatigue protocol during Sh elevation/rotation tasks vs. control group. | There was no significant change in BB muscle activity during Sh elevation/rotation tasks before and after a fatigue protocol. |
| Moon et al. (2013) | IV | 13 | Healthy subjects | Biceps curl at various degrees of Sh Flex (30°, 45°, 60°, 75° and 90°). | sEMG: (LHB, FCR). | The most significant BB muscle activity (115.65) and force (88.8N) occurred at an Sh Flex of 75°. BB showed substantial differences between a) 60° and 90° (p<0.05), b) 75° and 30° and 90° (p<0.01). | Supports an Sh angle of 75° for max BB activity and strength production. |
| Nejat et al. (2012) | III | 4 | Normal subjects | Sh Abd with and without 1-kg load | sEMG: (SHB, LHB). | In standing (Sh abduction) without load (0-kg) and with load (1kg), the dominant activity of the BB was in the LHB vs. SHB. | Supports LHB activity during Sh Abd |
| Oliveira et al. (2009) | III | 22 | Healthy subjects | 3 Biceps Curl variations:   - DBC (Sh Flex = 0°) - IDC (Sh E = 50°) - DPC (Sh Flex = 50°) | sEMG: (LHB). | All exercises demonstrated a significant level of LHB activation (concentrically > eccentrically), at least 50% of maximum RMS. The IDC and DBC resulted in similar patterns of BB activation for the whole elbow ROM. DPC elicited high muscle activation only for a short range of elbow joint angle. The concentric activity was higher and lower for DPC at phases 1 and 3, respectively, when compared to LHB activity during IDC and DBC, suggesting Sh Flex angle affects BB activation. | IDC and  DBC generate the most significant LHB activity throughout FROM of the elbow.  Potential for IDC and DBC strengthening of the conservative rehab protocols. |
| Rojas et al. (2009) | IV | 7 | Baseball pitchers | Windmill pitch vs Overhand pitch | sEMG (BB). | Maximum BB muscle activation during the overhand throw (19% ± 11% MMT) was significantly lower than during the windmill pitch (38% ± 16% MMT; P = .02). Windmill pitch - maximum BB activity occurred during phase 5 (9 o’clock) of the windmill pitch to ball release. Overhand throw - maximum BB activity occurred during phase 3 (arm cocking) of the overhand throw. | Supports the active role of BB during throwing underarm>overarm. |
| Sakurai et al. (1998) | IV | 11 | Asymptomatic Sh | Isometric Sh Flex and Abd in IR/ER positions with the elbow at 0° and 90° Flex + *elbow brace to minimise LHB at the elbow | sEMG: (LHB, SHB, AD). | Significantly increased LHB activity in arm elevation:   - Sh Flex 45° vs Sh Flex 135° in ER and elbow E (p = 0.0018) - Sh Abd 45° vs Sh Abd 135° in ER and elbow E (p = 0.025) - Sh Abd 45° vs Sh Abd 135° in IR and elbow E (p = 0.010) - Comparisons of LHB and SHB muscular activities demonstrated significantly higher activity in the LHB in IR than in the SHB (p < 0.05). | Supports most significant LHB activity during Flex and Abd of the Sh joint in ER.  Potential for IDC and DBC strengthening of the conservative rehab protocols. |
| Sasaki et al. (2019) | III | 16 | Healthy adults (30 Sh) | Isometric Sh ER (sitting and supine), under different loads (0kg, 0.5kg, 1kg, and 2 kg) | sEMG:  (ISP, UT, PD, BB). | A significant difference in BB muscle activity for all loads between 0 kg and 2 kg in the sitting position compared to supine (p < 0.01):   - BB muscle activity (%MVC) in the sitting position was significantly higher than that in the supine position (P<0.001) - BB muscle activity (%MVC) differed significantly with the amount of load applied (P=0.003) - For a 2 kg load, BB muscle activity (%MVC) was significantly higher than that for the 0 kg load in the sitting position (P<0.01) | Supports the more significant activity of BB in sitting with a load.  BB strengthening and progressive loading programs. |
| Swaringen et al. (2006) | IV | 13 | Healthy subjects | Orthopaedic special tests (OST) – ACT-supinated, ACT-pronated, BTT, ASST, PPT-supinated, PTT-pronated, BLT II | sEMG: (LHB, SHB). | All tests reported statistically significant differences in the normalised EMG signal of the LHB (P<0.0001). OST - Mean EMG (SEM) highest to lowest BB activity: ACT-s = 1.80 (0.26); BTT = 1.70 (0.19); ASST = 1.45 (0.22); ACT-p = 1.42 (0.26), PPT-s = 0.71 (0.08); BLT = 0.69 (0.11); PPT-p = 0.64 (0.33). No statistically significant differences in BB EMG activity were seen between forearm Sup (s) and forearm pronation (p) positions for the ACT and the PPT. | Supports LHB as an Sh flex.  Supports the use of the ACTs and BTT to generate max LHBT force on SLAP lesions |
| E. J. D. Veen et al. (2021) | III | 12 | Twelve patients with RC tear vs. health controls. | FIT-HaNSA functional reaching tasks | sEMG: (AD, MD, PD, PM, UT, LD, BB).  fwEMG (SSP, ISP, SSC). | Significant hyperactivity of the BB (p = 0.04) and PD (p = 0.03) muscles during reaching tasks in patients with RC tear vs. controls. Decreased activity of the BB (p = 0.03) and PD (p = 0.03) muscles during the downward phase in patients with RC tear vs. controls. Muscle activation patterns of BB and PD in the RC tear group were different to the control group with hyperactivity and early recruitment. | Supports hyperactivity, early recruitment and potential compensation of the BB and PD in patients with symptomatic RC tear during reaching ADLs. |
| Yamaguchi et al. (1997) | IV | 30 | Subjects with RC tear vs. health controls. | Sh ROM in scapular plane + *elbow brace to minimise LHB at the elbow. | sEMG:(BB, Br)  fwEMG:(SSP) | No statistically significant difference in BB activity in control subjects (1.7%-3.1%) vs. subjects with RC tears (1.6%-4.4%) for all Sh ROMs. | No significant differences in BB activity during Sh ROM. |

List of Abbreviations: Abduction (Abd); Active Compression Test (ACT); Anterior Deltoid (AD); Adduction (Add); Activities of Daily Living (ADL); Anterior Superior SLAP Test (ASST); Biceps Brachii (BB); Brachialis (Bc); Biceps Load Test (BLT); Brachioradialis (Br); Biceps Tension Test (BTT); Dumbbell Biceps Curls (DBC); Dumbbell Preacher Curls (DPC); Extension (E); External Rotation (ER); Fast Bowling (FB); Fine Wire Electromyography (fwEMG); Flexion (Flex); Force (N); Flexor Carpi Radialis (FCR); Functional Impairment Test-Hand and Neck/Shoulder/Arm (FIT-HaNSA); Incline Dumbbell Curls (IDC); Infraspinatus (ISP); Internal Rotation (IR); Latissimus Dorsi (LD); Level of Evidence (LOE); Long Head of Biceps (LHB); Lower Trapezius (LT); Manual Muscle Test (MMT); Massive Rotator Cuff Tears (MRCT); Maximal Voluntary Contraction (MVC); Middle Deltoid (MD); Middle Trapezius (MT); P-value (p); Pearson Correlation Coefficient (PCC); Posterior Deltoid (PD); Pectoralis Major (PM); Pain Provocation Test (PPT); Pronation (Pro); Maximum Voluntary Isometric Contraction (MVIC); Range of Motion (ROM); Rotator Cuff (RC); Root Mean Squared (RMS); Serratus Anterior (SA); Short Head of Biceps (SHB); Shoulder (Sh). Slow Bowling (SB); Surface Electromyography (sEMG); Standard Error of the Mean (SEM); Subscapularis (SSC); Supination (Sup); Supraspinatus (SSP); Triceps Brachii (TB); Teres Major (TM); Upper Trapezius (UT).

References

1. Ahamed NU, Sundaraj K, Ahmad B, Rahman M, Ali MA, Islam MA. Surface electromyographic analysis of the biceps brachii muscle of cricket bowlers during bowling. Australas Phys Eng Sci Med. 2014;37(1):83-95.

2. Baek S, Ki SY, Chung SW, Lee SJ, Cho YC, Oh KS. Delayed Anticipatory Muscle Activation in Rotator Cuff Tendinopathy. Orthop J Sports Med. 2021;9(7):23259671211019360.

3. Basmajian JV, Latif A. Integrated actions and functions of the chief flexors of the elbow: a detailed electromyographic analysis. J Bone Joint Surg Am. 1957;39-A(5):1106-18.

4. Borms D, Ackerman I, Smets P, Van den Berge G, Cools AM. Biceps Disorder Rehabilitation for the Athlete: A Continuum of Moderate- to High-Load Exercises. Am J Sports Med. 2017;45(3):642-50.

5. Brown JM, Solomon C, Paton M. Further evidence of functional differentiation within biceps brachii. Electromyogr Clin Neurophysiol. 1993;33(5):301-9.

6. Chalmers PN, Cip J, Trombley R, Cole BJ, Wimmer MA, Romeo AA, et al. Glenohumeral Function of the Long Head of the Biceps Muscle: An Electromyographic Analysis. Orthop J Sports Med. 2014;2(2):2325967114523902.

7. Cools AM, Borms D, Cottens S, Himpe M, Meersdom S, Cagnie B. Rehabilitation Exercises for Athletes With Biceps Disorders and SLAP Lesions: A Continuum of Exercises With Increasing Loads on the Biceps. Am J Sports Med. 2014;42(6):1315-22.

8. David G, Magarey ME, Jones MA, Dvir Z, Turker KS, Sharpe M. EMG and strength correlates of selected shoulder muscles during rotations of the glenohumeral joint. Clin Biomech (Bristol, Avon). 2000;15(2):95-102.

9. Furlani J. Electromyographic study of the m. biceps brachii in movements at the glenohumeral joint. Acta Anat (Basel). 1976;96(2):270-84.

10. Glousman R, Jobe F, Tibone J, Moynes D, Antonelli D, Perry J. Dynamic electromyographic analysis of the throwing shoulder with glenohumeral instability. J Bone Joint Surg Am. 1988;70(2):220-6.

11. Hawkes DH, Alizadehkhaiyat O, Fisher AC, Kemp GJ, Roebuck MM, Frostick SP. Normal shoulder muscular activation and co-ordination during a shoulder elevation task based on activities of daily living: an electromyographic study. J Orthop Res. 2012;30(1):53-60.

12. Hawkes DH, Alizadehkhaiyat O, Kemp GJ, Fisher AC, Roebuck MM, Frostick SP. Shoulder muscle activation and coordination in patients with a massive rotator cuff tear: an electromyographic study. J Orthop Res. 2012;30(7):1140-6.

13. Hawkes DH, Alizadehkhaiyat O, Kemp GJ, Fisher AC, Roebuck MM, Frostick SP. Electromyographic assessment of muscle fatigue in massive rotator cuff tear. J Electromyogr Kinesiol. 2015;25(1):93-9.

14. Jobe FW, Moynes DR, Tibone JE, Perry J. An EMG analysis of the shoulder in pitching. A second report. Am J Sports Med. 1984;12(3):218-20.

15. Kido T, Itoi E, Konno N, Sano A, Urayama M, Sato K. Electromyographic activities of the biceps during arm elevation in shoulders with rotator cuff tears. Acta Orthop Scand. 1998;69(6):575-9.

16. Kim SH, Ha KI, Kim HS, Kim SW. Electromyographic activity of the biceps brachii muscle in shoulders with anterior instability. Arthroscopy. 2001;17(8):864-8.

17. Levy AS, Kelly BT, Lintner SA, Osbahr DC, Speer KP. Function of the long head of the biceps at the shoulder: electromyographic analysis. J Shoulder Elbow Surg. 2001;10(3):250-5.

18. Lisowski JK, Oyama S, Hibberd EE, Myers JB, Prentice WE, Creighton RA. Biceps Brachii Does Not Play an Active Role in Humeral Movement. Athletic Training & Sports Health Care: The Journal for the Practicing Clinician. 2014;6(4):179-88.

19. Moon J, Shin I, Kang M, Kim Y, Lee K, Park J, et al. The Effect of Shoulder Flexion Angles on the Recruitment of Upper-extremity Muscles during Isometric Contraction. J Phys Ther Sci. 2013;25(10):1299-301.

20. Nejat N. Investigation on Muscle Compartments in the Biceps Brachii. Journal of Medical and Biological Engineering. 2012;32(4):251-7.

21. Oliveira LF, Matta TT, Alves DS, Garcia MA, Vieira TM. Effect of the shoulder position on the biceps brachii emg in different dumbbell curls. J Sports Sci Med. 2009;8(1):24-9.

22. Rojas IL, Provencher MT, Bhatia S, Foucher KC, Bach BR, Jr., Romeo AA, et al. Biceps activity during windmill softball pitching: injury implications and comparison with overhand throwing. Am J Sports Med. 2009;37(3):558-65.

23. Sakurai G, Ozaki J, Tomita Y, Nishimoto K, Tamai S. Electromyographic analysis of shoulder joint function of the biceps brachii muscle during isometric contraction. Clin Orthop Relat Res. 1998(354):123-31.

24. Sasaki S, Kenmoku T, Otera A, Miyajima G, Nagura N, Nakawaki M, et al. Electromyographic analysis of infraspinatus and scapular muscles during external shoulder rotation with different weight loads and positions. J Orthop Sci. 2019;24(1):75-80.

25. Swaringen JC, Mell AG, Langenderfer J, LaScalza S, Hughes RE, Kuhn JE. Electromyographic analysis of physical examination tests for type II superior labrum anterior-posterior lesions. J Shoulder Elbow Surg. 2006;15(5):576-9.

26. Veen EJD, Koorevaar CT, Verdonschot KHM, Sluijter TE, de Groot T, van der Hoeven JH, et al. Compensatory Movement Patterns Are Based on Abnormal Activity of the Biceps Brachii and Posterior Deltoid Muscles in Patients with Symptomatic Rotator Cuff Tears. Clin Orthop Relat Res. 2021;479(2):378-88.

27. Yamaguchi K, Riew KD, Galatz LM, Syme JA, Neviaser RJ. Biceps activity during shoulder motion: an electromyographic analysis. Clin Orthop Relat Res. 1997;336(336):122-9.
