# Supplementary material for: The long head of biceps at the shoulder: a scoping review
Source: BMC Musculoskelet Disord. 2023 Mar 28;24:232. doi: 10.1186/s12891-023-06346-5 (PMC10044783; doi:10.1186/s12891-023-06346-5)
Supplement: Supplementary file 7 — Supplementary Material 7 [file 12891_2023_6346_MOESM7_ESM.docx]

# Additional file 7: Supplementary Table 5_BMC.docx; Glenohumeral joint stability in vivo

| Author | LOE | No | Participants/intervention | Outcomes measure | Results | Implications |
| --- | --- | --- | --- | --- | --- | --- |
| Giphart et al. (2012) | II | 5 | Chronic biceps tenosynovitis / Arthroscopic TD vs. contralateral healthy Sh | Biplane fluoroscopy analysis of GHJ position with sEMG of BB muscle activity. | The mean difference in GHJ translation between TD and healthy Sh was always less than 1.0 mm. TD Sh were more anteriorly (centred) during abd (0.7 mm; p < 0.01) and during the eccentric phase of the simulated late cocking motion (0.9 mm; p < 0.02).  sEMG BB activity:   - Sh Abd - Nil significant difference in BB activation between TD and healthy contralateral Sh. - Simulated Late Cocking - Nil significant difference in activation between the TD and healthy Sh. - Simulated lifting - Nil significant difference in EMG activation between the TD and the healthy Sh. | Minimal role of BB in GHJ stability! |
| Glueck et al. (2003) | IV | 1 | Multidirectional instability with /  Arthroscopy with thermal capsulorraphy | Post-operative pain and instability. | Sh instability symptoms improved post arthroscopy with thermal capsulorraphy. LHB ABS may lead to Sh instability. | LHB’s role in GHJ stability! |
| Kido et al. (2000) | III | 18 | Patients with symptomatic RC tear vs controls | Radiological assessment of acromiohumeral interval distance during elevation. | HOH was significantly higher without BB contraction (p = 0.0011), vs. lower with BB contraction (p = 0.0012) vs controls. EMG showed significant increases in the BB muscle activity (% MVC) at all Sh angles of elevation (p < 0.0143). | BB active depressor of the HOH in patients with RC tear! |
| Landin et al. (2008) | IV | 30 | Volunteers with asymptomatic Sh /  Electrical stimulation of BB in variable degrees of Sh scaption and elbow Flex | Dynamometer - Sh joint angle and isometric torque (Nm) | Maximum BB moment occurred in the early phase of elevation from 0° to 30° of elevation and decreased significantly (about 65%) as the Sh elevation progressed to 60°, 90° and 120°. | BB is most active in the early phase of Sh elevation and reduces as elevation increases in normal Sh. |
| Walch et al. (2005) | III | 291 | Patients with irreparable FTT RC tear /  Arthroscopic LHB TT | Radiological assessment of acromiohumeral interval distance, pre-and post-TT. | Significantly (p<0.0001) reduced acromiohumeral interval distance post TT (5.3 +-2.9) compared to pre-operatively (6.6+- 2.7) in patients with concomitant RC tears. Significant progression of GHJ arthritis (p<0.001) post TT. | LHB’s role in HOH depression |
| J. J. P. Warner and P. J. McMahon (1995) | III | 7 | Symptomatic patients:  a) LHB ABS  b) intact LHB  c) pre and post TD | Radiological comparison of acromiohumeral distance in various degrees of Sh scaption. | A significant increase in superior GHJ translation of HOH was demonstrated (p<0.01) in Sh with an absent LHBT compared to those with an intact LHBT. Mean HOH translation:   - 45° scaption - Absent LHB (2.1 ± 1.6 mm) vs. Intact LHB (-0.1 ± 0.4mm) - 90° scaption - Absent LHB (2.4 ± 0.8) vs. Intact LHB (0.1 ± 0.4 mm) - 120 scaption - Absent LHB (2.9 ± 1.6) vs. Intact LHB (0.6 ± 0.8)   No significant difference in GHJ translation of HOH pre and post-biceps TD. | LHB role in HOH depressor during Sh elevation! |

List of Abbreviations: Abduction (Abd); Absence of LHB (ABS); Biceps Brachii (BB); Electromyography (EMG); Flexion (Flex); Full Thickness Tear (FTT); Glenohumeral Joint (GHJ); Head of Humerus (HOH); Level of Evidence (LOE); Long Head of Biceps (LHB); Maximal Voluntary Contraction (MVC); Newton-metres (Nm); P-value (p); Rotator Cuff (RC); Shoulder (Sh); Surface Electromyography (sEMG); Tenodesis (TD); Tenotomy (TT).

References

1. Giphart JE, Elser F, Dewing CB, Torry MR, Millett PJ. The long head of the biceps tendon has minimal effect on in vivo glenohumeral kinematics: a biplane fluoroscopy study. Am J Sports Med. 2012;40(1):202-12.

2. Glueck DA, Mair SD, Johnson DL. Shoulder instability with absence of the long head of the biceps tendon. Arthroscopy. 2003;19(7):787-9.

3. Kido T, Itoi E, Konno N, Sano A, Urayama M, Sato K. The depressor function of biceps on the head of the humerus in shoulders with tears of the rotator cuff. J Bone Joint Surg Br. 2000;82(3):416-9.

4. Landin D, Myers J, Thompson M, Castle R, Porter J. The role of the biceps brachii in shoulder elevation. J Electromyogr Kinesiol. 2008;18(2):270-5.

5. Walch G, Edwards TB, Boulahia A, Nove-Josserand L, Neyton L, Szabo I. Arthroscopic tenotomy of the long head of the biceps in the treatment of rotator cuff tears: Clinical and radiographic results of 307 cases. 2005. p. 238-46.

6. Warner JJ, McMahon PJ. The role of the long head of the biceps brachii in superior stability of the glenohumeral joint. J Bone Joint Surg Am. 1995;77(3):366-72.
