# Supplementary material for: The long head of biceps at the shoulder: a scoping review
Source: BMC Musculoskelet Disord. 2023 Mar 28;24:232. doi: 10.1186/s12891-023-06346-5 (PMC10044783; doi:10.1186/s12891-023-06346-5)
Supplement: Supplementary file 8 — Supplementary Material 8 [file 12891_2023_6346_MOESM8_ESM.docx]

# Additional file 8: Supplementary Table 6_BMC.docx; Glenohumeral joint arthrokinematics in vitro

| Author | LOE | No | Participants/Intervention | LHB load (rationale) | Results | Implications |
| --- | --- | --- | --- | --- | --- | --- |
| Alexander et al. (2013) | V | 10 | Cadaver Sh / Simulation LHB contraction force (20N) on passive HOH position during the application of an external GHJ translation force (30N - anterior, posterior, superior, and inferior) tested in neutral rotation with 0°, 30°, 60° and 90° of GHJ Abd in the scapular plane. | 20N (?) | LHBT loading significantly contributed to passive stability of the GHJ with reduced GHJ translations observed at 30° GHJ Abd in all directions; the most significant reduction in translation occurred anteriorly by 42.6% or 15.9mm (p<0.0005) and inferiorly by 73.3% or 17.9mm (p< 0.0005). | Role of LHB as HOH depressor and passive anterior GHJ stabiliser.  LHB surgery may impact GHJ stability. |
| Halder et al. (2001) | V | 10 | Cadaver Sh / Simulated loading of SSP, ISP, TMi, TM, LD, SSC, PM and LHB (proportion to their cross-sectional area) on HOH depression during the application of externally applied superior translation force (20N) in 0°, 30°, 60" and 90" of GHJ Abd.  *Muscle loading was based on the hypothesis that maximum muscle force is proportional to its cross-sectional area. | ? (PCSA) | LHB showed a considerable depression effect (average: 4.0 +/- 2.1 mm) and was equivalent as an effective HOH depressor to the ISP and SSC at 30° (6.0 +/- 1.9 mm) and 60° (4.3 +/- 1.8 mm) of GHJ Abd and less effective than both ISP and SSC in higher ranges (90°) of GHJ Abd (1.7 +/- 1.0 mm). | Role of LHB as HOH depressor.  A potential role for including LHB strengthening for Sh instability and impingement. |
| Hanypsiak et al. (2012) | V | 8 | Cadaver Sh / Simulated BB loading (10, 20, and 40N) on GHJ ROM and HOH position during a simulated 1) late-cocking throwing position in 90° degrees of Abd in the coronal plane 90° and maximum ER. 2) deceleration position in the scapular plane, neutral rotation, and maximum IR. | 10, 20 and 40N (PCSA) | LHB loading in the late-cocking phase reduced maximal Sh ER and moved the HOH anteriorly. Unloading LHB moves the HOH posteriorly during the late cocking phase. LHB loading in the deceleration phase resulted in a significant decrease in maximal Sh IR, and the HOH moved posteriorly, inferiorly, and medially.  SHB loading in the late-cocking phase significantly moved the HOH superiorly and significantly decreased maximum ER and peak GHJ contact pressure. | Role of LHB as GHJ compressor and limiter of ER rotation in positions of GHJ stability.  Potential for internal Sh impingement in throwing Sh following biceps TD for SLAP lesions. |
| Itoi et al. (1993) | V | 13 | Cadaver Sh / Replacement of LHB and SHB tendon attachment to a spring-loaded device to apply 0 kg, 1.5 kg, or 3 kg loads on the LHB or the SHB with the arm in Abd and 60°, 90° or 120° ER before and after applying a 1.5 kg anterior translation force to the HOH, with the capsule intact, vented or sectioned to simulate a Bankart lesion. | 1.5 and 3 kg (PCSA?) | With no LHB loading, maximum anterior HOH displacement occurred under a 1.5 kg anterior translation force at 60° ER, followed by intermediate displacement at 90° and the smallest displacement at 120° (p<0.0001). With LHB loading, anterior HOH displacement was significantly decreased with than without LHB loading at 60° ER (p<0.0001) and at 90° (p=0.0011). After the creation of a Bankart lesion, anterior HOH displacement was significantly decreased by LHB loading at 60° ER (p<0.0001), 90° (p<0.0001), and 120° (p<0.0001).  * Similar trends were observed for the SHB. | Role of LHB as anterior GHJ stabiliser.  A potential role for including LHB strengthening for anterior Sh instability. |
| Itoi et al. (1994) | V | 13 | Cadaver Sh / Sequential loading of the RC (SSP = 2kg, ISP = 4.8kg, SSC = 1.5kg) and BB (LHB = 0.75kg and SHB = 0.75kg) with the arm in Abd and 60°, 90° or 120° ER before and after applying a 1.5 kg anterior translation force to the HOH, with the capsule intact, vented, or sectioned to simulate a Bankart lesion.  *Loading of the RC and BB calculated from the proportional physiological cross-sectional areas of the muscles | BB = 1.5kg (LHB = 0.75kg, SHB = 0.75kg)/  (PCSA) | With the capsule intact, anterior HOH displacement in 90° ER, with the SSC loaded, was significantly larger than with the other muscles (p = 0.0009). With the capsule vented, anterior HOH displacement in 90° ER with the BB loaded was significantly smaller than that with the SSC loaded (p = 0.0052). After creating a Bankart lesion, anterior HOH displacement in 90° ER, with the BB loaded, was significantly less than with any RC muscles loaded (p = 0.0132).  * In Sh with an intact capsule, the contribution by the RC and BB muscles to HOH stability (mean displacement) was most significant from the ISP, BB, SSP and SSC compared to baseline. | Role of LHB as anterior GHJ stabilizer.  A potential role for including LHB strengthening for anterior Sh instability. |
| Kumar et al. (1989) | V | 15 | Cadaver Sh / Simulated BB (LHB and SHB) loading (3kg) on GHJ HOH position, measured by a reduction in acromiohumeral distance under Sh radiographs and tested in a neutral hanging position. | 3kg (?) | Statistically significant reduction in acromiohumeral distance and upward migration of the HOH on tensioning of the SHB by 21.2% (p<0.001). No substantial change in acromiohumeral distance was observed on tensioning the LHB alone (p>0.1). The tension of both heads of BB caused minimal superior migration of the HOH, with a 5.1% decrease in acromiohumeral distance observed. When the LHB was divided, a significant upward migration of the HOH was observed on tensioning both heads, with a 15.5% decrease in acromiohumeral distance (p<0.001). | Role of LHB as HOH depressor.  Potential implications for LHB surgery and resultant GHJ instability and dysfunction. |
| McGarry et al. (2016) | V | 8 | Cadaver Sh / Simulated BB loading (10, 20 and 40N) on GHJ ROM and HOH position, tested in 60° abduction in the scapula and coronal plane. | 10, 20, 40N (%PCSA) | LHBT loading significantly reduced GHJ ROM and HOH position.  Rotational ROM:   - Loading LHBT significantly decreased IR in both the scapular (17.9%; P=0.001) and coronal planes (5.7%; P =0.04) and ER in the scapular plane (2.6%; P=0.02) compared with the BB unloaded.   HOH position:   - Loading LHB shifted HOH posteriorly in neutral rotation in scapular plan (p=0.02) - Loading LHB shifted HOH anteriorly in maximal ER in both coronal (p=0.2) and scapular planes (p=0.009) - Loading LHB shifted HOH inferiorly in maximal IR p<0.001) and neutral rotation (p=0.03) in a scapular plane compared to unloaded. - With the LHB unloaded, there was a significant superior shift with SHB loading in both planes, with the most considerable difference seen at maximum IR in the scapular plane (P = 0.006) and coronal plane (P = 0.01) | LHB role as HOH depressor and GHJ stabiliser.  Potential Sh impingement following TD in throwers. |
| Pagnani et al. (1995) | V | 7 | Cadaver Sh / Simulated LHBT contraction force (55N) during the application of externally applied GHJ translation force (50N) in anterior, posterior, superior, and inferior directions, under a 20N GHJ compressive load, tested in three positions of IR-ER in 0°, 45°, 90° of elevation (scaption) without and with a 1) simulated anterosuperior lesion and 2) a complete superior labrum lesion. | 55N (PCSA) | Effect of Anterosuperior lesion:   - No significant effect on Anteroposterior (AP) and Superoinferior (SI) translation with or without LHB loading.   Effect of complete superior labral lesion:   - Significant AP (p = 0.04) and SI (p < 0.0001) translations of the HOH at 45 and 90° of elevation. Significant AP (p = 0.04) and SI (p = 0.006) translations at 0° of elevation.   Anteroposterior (AP) translation compared to vented condition:   - 4.0mm increase in AP translation at 90° elevation and IR (p = 0.004). - 6.0mm increased AP translation at 45° elevation and neutral rotation (p<0.0001). - 6.3mm increase in AP translation at 45° of elevation and IR (p<0.0001). - 3.7mm increase in AP translation in 0° elevation and neutral rotation (p = 0.04).   Superoinferior (SI) translation compared to vented condition:   - 1.9mm increase in neutral (p=0.05) - 2.5 mm increase in IR (p=0.01) - 2.5 mm increase in ER (p=0.01) - 3.8 mm increase in 0° elevation and neutral rotation (p = 0001).   Effect of the simulated superior labral lesion with LHB loading:   - Anteroposterior (AP) translation compared to the vented condition - 3.6 mm increase in posterior translation at 90° of elevation and ER (p = 0.009). - 6.8mm increase in AP translation at 45° of elevation, neutral rotation (p < 0.0001). - 9.0mm increase in AP translation at 0° (p = 0.004)   Superoinferior (SI) translation compared to vented condition:   - 5.3 mm increase in neutral rotation (p < 0.0001) - 4.5 mm increase in IR (p < 0.0001) - 3.5 mm increase in ER (p = 0.0004) - 5.4 mm increase in 0° (p = 0.003) | Role of LHB as GHJ stabiliser.  A strong relationship between SLAP lesions and Sh instability. |
| Pagnani et al. (1996) | V | 13 | Cadaver Sh / Simulated LHB contraction force on HOH position, during the application of an external GHJ translation force (50N - anterior, posterior, superior and inferior), under a 22N GHJ compressive load. Tested in several GHJ elevation and rotation positions. | 55N (PCSA) | Application of a force to the LHB resulted in statistically significant decreases in HOH translation (p<0.0001) at 45° and 90° elevation (p<0.0001) and lower ranges of GHJ elevation - 0° (p=0.0006). In 45° elevation and neutral rotation, application of an LHBT force reduced anterior GHJ translation by 10.4mm (p=0.001), inferior by 5.3mm (p=0.01) and superior by 1.2mm (p=0.004). At 0° elevation and neutral rotation, applying an LHBT force significantly reduced anterior GHJ translation by 9.2mm (p=0.02) and deceased inferior translation by 5.7mm (p=0.01) compared with the vented condition. | Role of LHB as HOH depressor and GHJ stabiliser.  A potential role for including LHB strengthening for Sh instability and impingement |
| Patzer et al. (2011) | V | 21 | Cadaver Sh / Simulated LHBT contraction force (preload) during the application of externally applied GHJ translation force (50N) in anterior, posterior, anterosuperior, and anteroinferior directions, under a 20N GHJ compressive load, tested in 0°, 30°, 60° of Abd without and with the anteroinferior capsule sectioned to simulate a Bankart lesion.  *Preload based on % of maximal LHB force of 55N. LHB passive preload = 5N (10% of 55N). LHB active preload = 25N (50% of 55N) | 5N (%PCSA) | SLAP lesions lead to a significant increase in anterior and anteroinferior translational Sh instability*:*   - Increased anterior 18.8% under 5N LHB passive preload - Increased anterior 53.8% under 25N LHB active preload (p < 0.01) - Increased anteroinferior under 5N passive (7.5%); 25N active (45.9%) preload (p < 0.01) - Increased anterosuperior under 5N passive (26.6%); 25N active (30.9%) preload (p < 0.01). - A significantly larger increase (p < 0.05) in translation was observed under active (25N) vs passive (5N) LHB tension. The maximum increase in translation occurred at 60° in a dominant anterior direction.   After a SLAP lesion:   - Mean stabilising effect of LHB was significantly less in anterior (14.4%, p < 0.01) and anteroinferior direction (4.5%, p < 0.05) and almost similar in anterosuperior direction (118.1%, p > 0.05).   Intact Sh:   - Maximum stabilising function of LHB was observed for anterosuperior (125.5%), followed by anterior (48.1%) and anteroinferior (41.8%) translation at 60°, followed by 30° and 0° of Abd.   SLAP lesions with and without LHB TT:   - GHJ translation was significantly higher in SLAP lesions without LHB TT than after isolated LHB TT. - Significant increase in anterior and anteroinferior translation under 5N LHB passive preload than isolated LHB TT with an intact SLAP complex (p <0.05). - A significantly higher increase in anterosuperior translation after LHB TT than an isolated SLAP lesion under 25N LHB active preload (p < 0.01).   Comparisons of intact and defect SLAP complex*:*   - LHB decreases instability significantly more with an intact than with a defect SLAP complex (p < 0.01), substantially more under active (25N) than under passive (5N) LHB preload (p < 0.01). | Role of LHB as GHJ stabiliser. SLAP lesions lead to increased anterior and anteroinferior GHJ instability.  A potential role for LHB TT is to reduce GHJ translation in the presence of a SLAP lesion. |
| Rodosky et al. (1994) | V | 7 | Cadaver Sh / Dynamic simulated loading of the RC and LHB (0%, 25%, 50%, 75%, 100% extrapolated maximum force value) during GHJ Abd and ER (late cocking position of pitching) without and with the superior labral lesion.  *100% force values extrapolated from EMG prediction during the late cocking phase of throwing. | ? (PCSA + %MVC) | Normal Sh:   - LHB loading – As muscle force increased to 100% predicted value, torsional rigidity significantly increased to 24.50 Nm/radian, representing a 32% increase (p = 0.0106), compared to the unloaded torsional rigidity force of 18.59 N-m/radian.   Sh with superior labral lesions:   - Peak torque during LHB loading – For each biceps force level, peak torque was found to be significantly less (p < 0.01 in the Sh with superior labral lesions as compared with the normal Sh for each biceps muscle force level. - Mean peak torque in the normal Sh group was 12% higher than in the Sh with a superior labral lesion. Torsional rigidity during LHB loading – For each biceps force level, the torsional rigidity was significantly greater (p < 0.01). Mean torsional rigidity in the normal Sh was 19% higher at zero biceps muscle force. | Role of LHB as GHJ stabiliser.  A potential role for including LHB strengthening for Sh instability. |
| Su et al. (2010) | V | 10 | Cadaver Sh / Simulated LHBT loading on HOH position during applying externally applied GHJ translation forces (10, 20, 30, 40, and 50N) in both intact and sequentially larger RC tears. | 55N (PCSA) | Significant decrease in both anterosuperior and superior GHJ translation in intact specimens and for all sizes of RC tears during loading of the LHBT (P<0.04 for all values). | LHB role as HOH depressor and anterior GHJ stabiliser.  A potential role for including LHB strengthening for RC-related Sh pain.  Potential LHB overload in RC-related Sh pain.  Potential biomechanical consequences for Sh dysfunction following TT and TD. |
| Youm et al. (2009) | V | 6 | Cadaver Sh / Simulated LHBT loading (0, 11 and 22N) on 1) GHJ rotation, 2) GHJ translation, and 3) Path of GHJ articulation (PGA) as the HOH rotates from maximal IR (follow through) to 30°, 60°, 90°, and maximal ER (Late cocking) under a 22N GHJ compressive force. Tested in 90° humerothoracic abduction.  *Loading of the LHB calculated from a predicted 55N of maximum moment x 20 % of maximal manual muscle activity (55 x 20% = 11N and doubled = 22N. | 11 and 22N (PCSA + %MVC) | GHJ rotation ROM:   - 22N biceps load - Significant decreased GHJ IR (13.33°, p = 0.007) and ER (5.0°, p = 0.0003) ROM compared with the unloaded group.   GHJ translation:   - 22N biceps load - Significant decreased anterior (1.6 mm, p = 0.0028), posterior (7.0 mm, p = 0.014), superior (1.2 mm, p = 0.02) and inferior (9.6 mm, p = 0.001) translations compared with the unloaded group.   Path of GHJ articulation (PGA)*:*   - 22N biceps load – In maximum IR, a significant shift in Humeral Rotation Centre (HRC) occurred posteriorly (p = 0.034) compared with the unloaded HRC. - 11N and 22N at 30° ER significantly shifted HRC posteriorly (p = 0.047 and p = 0.003), respectively, compared with the unloaded HRC. - 11N and 22N at 60° ER significantly shifted HRC posteriorly (p = 0.027 and p = 0.013), respectively, compared with the unloaded HRC. No significant shifts in HRC occurred at 90° ER. - 22N biceps load – In maximum ER, a significant shift in HRC occurred anteriorly (p=0.027) and superiorly (p=0.0058). | Role of LHB as GHJ stabiliser.  A potential role for including LHB strengthening for Sh instability. |

List of Abbreviations: Abduction (Abd); Active Joint Position Sense (AJPS); Biceps Brachii (BB); Electromyography (EMG); External Rotation (ER); Force (N); Glenohumeral Joint (GHJ); Head of Humerus (HOH); Humeral Rotation Centre (HRC); Infraspinatus (ISP); Internal Rotation (IR); Latissimus Dorsi (LD); Level of Evidence (LOE); Long Head of Biceps (LHB); Long Head of Biceps Tendon (LHBT); Maximal Voluntary Contraction (MVC); Newtons-metres (Nm); P-value (p); Passive Joint Position Sense (PJPS); Pectoralis Major (PM); Path of GHJ Articulation (PGA); Proportional to Cross Sectional Area (PCSA); Range of Motion (ROM); Rotator Cuff (RC); Short Head of Biceps (SHB); Shoulder (Sh); Subscapularis (SSC); Superior Labrum Anterior Posterior (SLAP); Supraspinatus (SSP); Tenodesis (TD); Tenotomy (TT); Teres Minor (TMi); Teres Major (TM).

References

1. Alexander S, Southgate DF, Bull AM, Wallace AL. The role of negative intraarticular pressure and the long head of biceps tendon on passive stability of the glenohumeral joint. J Shoulder Elbow Surg. 2013;22(1):94-101.

2. Halder AM, Zhao KD, Odriscoll SW, Morrey BF, An KN. Dynamic contributions to superior shoulder stability. J Orthop Res. 2001;19(2):206-12.

3. Hanypsiak B, Nguyen M, McGarry M, Morgan C, Gupta R, Lee TQ. The Role of the Biceps Brachii in Overhead Throwing: A Biomechanical Study (SS-06). Arthroscopy: The Journal of Arthroscopic & Related Surgery. 2012;28(6):e4.

4. Itoi E, Kuechle DK, Newman SR, Morrey BF, An KN. Stabilising function of the biceps in stable and unstable shoulders. J Bone Joint Surg Br. 1993;75(4):546-50.

5. Itoi E, Newman SR, Kuechle DK, Morrey BF, An KN. Dynamic anterior stabilisers of the shoulder with the arm in abduction. J Bone Joint Surg Br. 1994;76(5):834-6.

6. Kumar VP, Satku K, Balasubramaniam P. The role of the long head of biceps brachii in the stabilization of the head of the humerus. Clin Orthop Relat Res. 1989(244):172-5.

7. McGarry MH, Nguyen ML, Quigley RJ, Hanypsiak B, Gupta R, Lee TQ. The effect of long and short head biceps loading on glenohumeral joint rotational range of motion and humeral head position. Knee Surg Sports Traumatol Arthrosc. 2016;24(6):1979-87.

8. Pagnani MJ, Deng XH, Warren RF, Torzilli PA, Altchek DW. Effect of lesions of the superior portion of the glenoid labrum on glenohumeral translation. J Bone Joint Surg Am. 1995;77(7):1003-10.

9. Pagnani MJ, Deng XH, Warren RF, Torzilli PA, O'Brien SJ. Role of the long head of the biceps brachii in glenohumeral stability: a biomechanical study in cadavera. J Shoulder Elbow Surg. 1996;5(4):255-62.

10. Patzer T, Habermeyer P, Hurschler C, Bobrowitsch E, Paletta JR, Fuchs-Winkelmann S, et al. Increased glenohumeral translation and biceps load after SLAP lesions with potential influence on glenohumeral chondral lesions: a biomechanical study on human cadavers. Knee Surg Sports Traumatol Arthrosc. 2011;19(10):1780-7.

11. Rodosky MW, Harner CD, Fu FH. The role of the long head of the biceps muscle and superior glenoid labrum in anterior stability of the shoulder. Am J Sports Med. 1994;22(1):121-30.

12. Su WR, Budoff JE, Luo ZP. The effect of posterosuperior rotator cuff tears and biceps loading on glenohumeral translation. Arthroscopy. 2010;26(5):578-86.

13. Youm T, ElAttrache NS, Tibone JE, McGarry MH, Lee TQ. The effect of the long head of the biceps on glenohumeral kinematics. J Shoulder Elbow Surg. 2009;18(1):122-9.
