# Supplementary material for: The long head of biceps at the shoulder: a scoping review
Source: BMC Musculoskelet Disord. 2023 Mar 28;24:232. doi: 10.1186/s12891-023-06346-5 (PMC10044783; doi:10.1186/s12891-023-06346-5)
Supplement: Supplementary file 9 — Supplementary Material 9 [file 12891_2023_6346_MOESM9_ESM.docx]

# Additional file 9: Supplementary Table 7_BMC.docx; Shoulder proprioception

| Author | LOE | No | Participants | Intervention | LHB load (rationale) | Results | Implications |
| --- | --- | --- | --- | --- | --- | --- | --- |
| Ager et al. (2020) | I | 11 | Systematic review  447 (600 Sh - 232 pain vs. 388 pain-free). | Sh proprioception and Sh pain:   - (AJPS) Active joint position sense - (PJPS) Passive joint position sense - Kinesthesia - Sense of force | NA | Sh pain:   - AJPS *-* conflicting evidence in painful vs. pain-free Sh. - PJPS *-* inconclusive evidence in painful vs. pain-free Sh. The trend for unaltered PJPS in painful Sh - Kinesthesia *-* moderate evidence in painful vs. pain-free Sh. - Sense of force *-* insufficient evidence in painful vs. pain-free Sh. | Sh pain may decrease the sense of Kinesthesia and potentially a sense of force. No apparent effect on PJPS and conflicting evidence for AJPS. |
| Fyhr et al. (2015) | II | 17 | Systematic review | Sh proprioception and pathology:   - AJPS - PJPS - Movement sense (TTDPM) | NA | Post-traumatic GHJ instability:   - Moderate evidence that, overall, AJPS (p=0.06) and PJPS (p=0.19) did not differ significantly from the control groups - Limited evidence for deficits for AJPS (p<0.05) and moderate evidence for deficits for PJPS (p<0.001) compared to the contralateral uninjured Sh - Moderate evidence exists for deficits in motion sense, reflected by increased TTDPM, for the involved - Sh for patients compared to controls (p<0.001) and compared to the contralateral uninvolved Sh (p< 0.001).   Chronic RC pain:   - Limited evidence that AJPS is decreased compared to healthy controls | Movement sense and PJPS > AJPS are likely impaired after post-traumatic Sh instability. |

List of Abbreviations: Active Joint Position Sense (AJPS); P-value (p); Passive Joint Position Sense (PJPS); Level of Evidence (LOE); Shoulder (Sh); Threshold to Detection of Passive Motion (TTDPM).

References

1. Ager AL, Borms D, Deschepper L, Dhooghe R, Dijkhuis J, Roy J-S, et al. Proprioception: How is it affected by shoulder pain? A systematic review. Journal of Hand Therapy. 2020;33(4):507-16.

2. Fyhr C, Gustavsson L, Wassinger C, Sole G. The effects of shoulder injury on kinaesthesia: a systematic review and meta-analysis. Man Ther. 2015;20(1):28-37.
