# Supplementary material for: The long head of biceps at the shoulder: a scoping review
Source: BMC Musculoskelet Disord. 2023 Mar 28;24:232. doi: 10.1186/s12891-023-06346-5 (PMC10044783; doi:10.1186/s12891-023-06346-5)
Supplement: Supplementary file 10 — Supplementary Material 10 [file 12891_2023_6346_MOESM10_ESM.docx]

# Additional file 10: Supplementary Table 8_BMC.docx; Pathology of the LHB

| Author | LOE | No | Participants (sh) | Diagnostic Intervention | Results | Implications |
| --- | --- | --- | --- | --- | --- | --- |
| D. P. Beall et al. (2003) | III | 111 | Sh pain | MRI + diagnostic arthroscopy + open Sh surgery | Patients with LHBT tears were significantly more likely to have SSC tears (p<0.0001) and SSP tears (p<0.008) than those patients without LHBT tears.  Prevalence:   - LHBT tear + SSP RC tear (96.2%) - LHBT + ISP RC tear (34.6%) - LHBT + SSC RC tear (47.1%)   Positive predictive value (PPV):   - LHBT tear + SSP RC tear (95.8%) - LHBT tear + ISP RC tear (80%) - LHBT tear + SSC RC tear (61%) | LHB tendinopathy + SSC tear  LHB tendinopathy + SSP tear |
| Braun et al. (2011) | II | 229 | Sh pain:  RC disease (121)  Instability (50)  GHJ OA (43)  Other (15) | Arthroscopic Sh surgery | LHB pathology:   - Absent LHBT = 9.2% - Biceps pulley tear + increased age (p<0.001) - Biceps pulleys tear + SLAP (p=0.003) - Biceps pulley tear + LHBT instability (p<0.001) - Biceps pulley tears and RC lesions (p<0.001) - Biceps pulley tears + SSP (p<0.001) - Biceps pulley tears + ISP (p=0.004) - Biceps pulley tears + SSC = (p≤0.001) | Biceps pulley lesion + age  Biceps pulley lesion + SLAP  Biceps pulley lesion + LHBT instability  Biceps pulley lesion + RC tears |
| Candela et al. (2021) | III | 202 | RC-related Sh pain | Arthroscopic Sh surgery | LHB pathology:   - Absent LHBT = 10.9% - LHBT absent + massive RC tears (p<0.001). - LHBT inflammation + severity of RC tear (p<0.001) - LHBT flattening + severity of RC tear (p<0.001) - LHBT loss of integrity (delamination) + severity of RC tear (p<0.001) | ABS + RC tears  LHB tendinopathy + RC tears |
| Chae et al. (2020) | IV | 43 | RC-related Sh pain (CISTS and RC tear)  * Concealed intra-tendinous SSC tears (CIST) | Imaging (MRI), physical examination + arthroscopic Sh surgery | LHBT subluxation (p=0.0006), axial imaging and a medial biceps tendon lesion (sentinel sign) under arthroscopy (p=0.0003) were the most independent risk factor for predicting a CIST. | LHBT instability + SSC tear |
| C.-H. Chen et al. (2005) | III | 120 | RC-related Sh pain (n=122) | Clinical exam, imaging and arthroscopic +/- open Sh surgery | LHBT pathology:   - LHB tendinitis in 41% of patients undergoing surgery - LHBT subluxation in 8% of patients undergoing surgery - LHBT dislocation in 10% of patients undergoing surgery - LHBT partial tear in 12% of patients undergoing surgery - LHBT complete rupture in 5% of patients undergoing surgery   LHBT lesion (n=93) + RC tear:   - LHBT + SSP (43%) - LHBT + SSP + ISP (25%) - LHBT + SSC (5%) - LHBT + SSP + SSC (16%) - LHBT + SSP + ISP + SSC (11%) - LHBT + RC tear > 5 cm2 (94%) - LHBT + Large/massive RC tear (92%) - LHBT + (RC tear + SSC tear) (97%) - LHBT + SSC tear (97%)   LHBT instability + SSC tear:   - LHBT dislocation (n=12) + SSC tear (83%) - LHB subluxation (n=10) + SSC tear (80%) | LHB tendinopathy + RC tears  LHB instability + SSC tear |
| Chen et al. (2012) | III | 176 | RC-related Sh pain | Arthroscopic/open Sh surgery | Incidence of LHBT pathology in RC tears (SSP, ISP SSC):   - Patients with chronic RC tears > 3 months were more likely to have concomitant LHBT pathology compared with those < 3 months (97% vs 64%, p<0.001). - Patients with large RC tears > 5 cm2 were more likely to have combined LHBT pathology than those with a small (< 5 cm2) RC tear (93% vs. 76%, p=0.004). - Patients with single tendon RC tears were less likely to have LHB pathology than those with multiple tears (71% vs. 94%, p<0.001).   Incidence of LHBT instability and SSC tendon tears:   - Significant correlation between LHBT pathology and SSC tendons tears compared to intact SSC tendons (97% vs. 75%, p<0.001). - LHBT instability (subluxation or dislocation) + SSC tear (48%). - LHBT instability (subluxation or dislocation) + intact SSC (9%). | LHB tendinopathy + RC tears  LHB instability + SSC tear |
| Deutch et al. (2005) | III | 11 | LHBT rupture vs contralateral side | PROMS of subjective pain and strength  Isometric elbow/forearm strength and endurance  MRI – a) LHBT position/pathology in biceps groove, b) BB muscle volume/atrophy | Subjective pain and strength:   - Seven of 11 (64%) patients reported pain, and 4 (36%) patient-reported strength loss following LHBT rupture.   Elbow/forearm strength and endurance:   - Mean loss of 27% elbow strength (Flex = 29%, pronation = 28%, Sup = 23%). Mean loss of 20% forearm endurance (Sup = 20% and Pro = 19%). Mean loss of 28% screwdriver endurance (Sup = 31%, Pro = 24%).   MRI evaluation:   - Displaced LHB muscle demonstrated no change in total muscle volume, size, or signs of degeneration. | Patients with LHBT rupture and displacement report pain > weakness and suffer from permanent elbow/forearm strength and endurance loss |
| Dirkx and Pouliart (2020) | I | 336 | RC-related Sh pain | Imaging (DUS, CTA, MRI or MRA) vs arthroscopic + open Sh surgery | LHBT instability on imaging was significantly correlated with SSC tendon tears on all imaging modalities and surgery, except for MRI:   - DUS (*p=0.01) - MRI (p=0.61) - MRA (*p=0.01) - CTA (*p=0.01) - Surgery (*p=0.01) | LHBT instability + SSC tear |
| Habermeyer et al. (2004) | IV | 89 | Biceps pulley lesion | Arthroscopic Sh surgery | 89.9% of patients with a biceps pulley lesion demonstrated LHB pathology (synovitis, subluxation, dislocation, and partial or complete tearing). 43.8% of patients with a biceps pulley lesion showed Anterosuperior Impingement (ASI). ASI was significantly more prevalent in patients with SSC tears (P<0.0001).   - Pulley lesion + SSP = 24% - Pulley lesion + SSC = 25 % - Pulley lesion + SSP and SSC = 22% | Biceps pulley lesion + ASI |
| Hanusch et al. (2016) | III | 175 | Ultrasound of the LHBT sheath | Arthroscopic Sh surgery | A high significant correlation (p<0.001; *ρ=0.354) between the fluid in the LHB sheath and RC tears on arthroscopy. Weak significant correlation between fluid in the LHB sheath and both a) LHB tendon disease (p=0.012; *ρ=0.203) and b) GHJ disease (p=0.020; *ρ=0.187) under arthroscopy. Increased likelihood of RC tear (odds ratio, 2.641; 95% CI, 1.229-5.674) and biceps tendon disease (odds ratio, 2.698; 95% CI, 1.216-5.987) with LHBT sheath effusion.  *ρ = Spearman coefficient | LHBT sheath effusion + RC tears |
| Hawi et al. (2017) | III | 382 | RC disease or isolated pulley lesions | Arthroscopic RC or interval surgery | Biceps pulley lesions in 90.3% (345) of RC tears (SSP + SSC)  SLAP lesions in 25–62% of bicep pulley lesions. | Biceps pulley lesion + RC tears |
| Ilahi et al. (2002) | III | 107 | Sh pain (108): RC tears, instability, SLAP, labral tear, biceps tendon rupture, arthritis, calcific tendinitis, ACJ pathology, adhesive capsulitis. | Arthroscopic Sh surgery | Anatomical variation of the anterosuperior glenoid labrum present in 27 Sh:   - Sublabral foramen (hole) 18.5% - Buford complex (6.5%) - The incidence of major SLAP lesions was significantly higher in the 27 Sh with anterosuperior glenoid labrum variations (P<0.005) | Anterosuperior labrum variants + SLAP |
| Ilahi et al. (2008) | IV | 321 | Sh pain (n=334): Anterosuperior glenoid labrum variants and associated Sh pathology. | Arthroscopic Sh surgery | 35.3% of Sh had an anterosuperior glenoid labrum variant. Of the 86 Sh with labrum variants, 48.8% had a significant/advanced SLAP lesion compared with Sh with no anterosuperior labrum variant (23.6%, P<.001). | Anterosuperior labrum variants + SLAP |
| Kim et al. (2020) | II | 191 | RC-related Sh pain | Imaging (MRI) + arthroscopic Sh surgery | The prevalence of an LHBT tear (p=0.006 and p=0.011) and malposition (p<0 .001) of the LHBT on MRI were significantly greater in patients with intermediate to high-grade SCC tears (≥ 50% of the cross-sectional area) under arthroscopy. | LHBT tear + SSC tear  LHBT malposition + SSC tear |
| Lafosse et al. (2007) | IV | 200 | RC-related Sh pain:  1-tendon RC tear = 93  2-tendon RC tears = 80  3-tendon RC tears = 27 | Arthroscopic RC surgery | Increased prevalence of LHBT lesions in 85% of patients with LHBT instability compared to without 30% (p<0.0001). RC tear size is strongly associated with the grade of LHB lesion, becoming more significant with increasing RC tear size.  1-tendon RC tear:   - 17% of SSP tears had a partial LHB lesion (>50%) - 35% of SSC tears had a partial LHB lesion (>50%)   2-tendon RC tear:   - 46% of RC tear (SSC and SSP) had a partial LHB lesion (>50%) - 47% of RC tear (ISP and SSP) had a partial LHB lesion (>50%)   3-tendon RC tear:   - 78% of RC tears (SSC, SSP and ISP) had a partial LHB lesion (>50%)   LHBT lesions in 55% of patients with RC tears:   - 3-tendon RC tears (SSC, SSP and ISP) = 78% - 2-tendon RC tears (SSC and SSP) = 46% - 2-tendon RC tears (ISP and SSP) = 47%   LHBT instability:   - LHBT instability (subluxation/dislocation) in 45% (89/200) of patients with RC tears - LHBT instability in 100% of patients with SSP RC tears (FTT = 70%, PTT = 30%) - LHBT anterior instability in 97% of patients with SSC RC tears - LHBT anterior instability (dislocation > subluxation) significantly more frequent in SSC tears > 1/3 (p=0.026) | LHBT instability + LHBT lesion  LHB tendinopathy + RC tears  LHBT instability + RC tears |
| Lakemeier et al. (2010) | III | 116 | Sh pain | Arthroscopic LHB TT  Histopathological study of LHBT tissue samples | Higher VEGF expression in LHBT samples in patients with RC tear (p < 0.05). Higher VEGF expression in LHBT samples was observed in patients with articular-sided RC tears compared to bursal-sided partial thickness RC tears (p< 0.05).  *Vascular Endothelial Growth Factor (VEGF) = neo angiogenesis in LHBT tissue samples.  Higher vessel density and vessel size in LHBT samples with cuff arthropathy (p < 0.05). | LHB tendinopathy + RC tears |
| Malavolta et al. (2016) | III | 93 | RC-related Sh pain | Imaging (MRI), arthroscopic Sh surgery | LHBT instability (p<0.001) on MRI and age (p=0.002) were predictive factors for the presence of SSC tears under arthroscopy. | LHBT instability + SSC tear |
| Mazzocca et al. (2013) | IV | 32 | Sh pain: Bicep instability, tendinosis, and degenerative GHJ disease (OA). | LHBTs of  patients undergoing TD  *Histopathological study of 1) pathological and 2) normal LHBT tissue samples | A significant degree of degeneration of the proximal (intra-articular) regions of the LHBT when compared with the distal biceps in all pathologic conditions (instability, p=0.001; tendinosis, p=0.005; degenerative GHJ disease, p=0.008). | Proximal LHBT degeneration in biceps instability, tendinosis and GHJ OA |
| Mehta et al. (2020) | III | 354 | Asymptomatic and symptomatic RC tear | DUS of RC and LHBT pathology | A higher incidence of LHBT pathology was observed in Sh with SSC tears (71%) compared to those with an intact SSC (12%) (p<0.01):   - LHBT dislocation/subluxation + SSC tear = 74% - LHBT absent + SSC tear = 20% - LHBT partial tear + 6% | LHBT instability + SSC tear |
| Murthi et al. (2000) | II | 200 | Sh pain:  Impingement syndrome/RC pathology. | Arthroscopic subacromial decompression + 1) TD or 2) tenosynovectomy  *Histopathological study of LHBT tissue samples | LHB tendinopathy + RC tear in 91% of 80 Sh undergoing TD for loss of structural integrity. LHB tendinopathy + partial thickness RC tear 84%. LHB tendinopathy + full thickness RC tear 89%. | LHB tendinopathy + RC disease |
| Neviaser et al. (1982) | IV | 89 | Sh pain (Painful arc syndrome). | Sh arthroplasty | Tenosynovitis of the LHBT in 100% painful arc syndrome (RC disease) + LHB tendinopathy | LHB tendinopathy + RC disease |
| Özer et al. (2020) | III | 3129 | Painful Sh:  Buford complex SLAP lesions and instability. | Arthroscopic Sh surgery | Buford complex in 83 (2.65%) of Sh. Significant correlation between Buford complex (81.9%) and SLAP lesions (P<0.001) vs those without (33.1%).  Higher incidence of Buford complex observed in posterior Sh instability (1.2% vs. 0.9%, p = 0.789). vs anterior instability (10.8% vs. 19.3%, p=0.052).  The incidence of Buford complex in patients with and without labral pathologies was 4.6% and 0.3%, respectively (P<0.001). | Buford complex + SLAP  Buford complex + posterior instability |
| Petersson (1986) | V | 77 | Cadaver Sh (n=153) | Sh dissection | Medial subluxation of the LHBT in 6.5% (5) subjects were found with full-thickness SSP tears (100%). Medial dislocation of the LHBT typically occurred (1) over the SSC associated with rupture of the biceps sling or (2) more commonly under the SSC tendon sliding medial to the lesser tuberosity and often associated with a partial SSC tear. | LHBT instability + SSP tear  LHBT instability + SSC tear |
| Redondo-Alonso et al. (2014) | IV | 599 | LHBT lesions + chronic SSP tendinopathy | NA | Epidemiological relationship between chronic SSP and LHB tendinopathy.  The percentage of associated lesions of LHBT and SSP tendinopathy varied between 78.5% and 22%, with a significant prevalence in the studies with smaller sample sizes. | LHBT + SSP tendinopathy |
| Schmalzl et al. (2019) | III | 22 | Patients undergoing  RC repair, LHB TD, or Sh arthroplasty. | Open or arthroscopic Sh surgery  *Histopathological study of LHBT tissue samples | Inflamed LHB (tendinitis), presented with histological signs of collagen disorganisation, infiltration by inflammatory cells, neovascularisation, extensive neuronal innervation) and a significantly increased inflammatory marker gene expression. | LHB tendinitis and pro-inflammatory mediators, neovascularisation, and gene expression. |
| Shah et al. (2016) | III | 66 | Sh pain + SSC tendon tears | Sh Arthroscopy | Shallow bicipital groove + LHBT pathology (p=0.001).  Shallow bicipital groove + SSC tendon pathology (p=0.01). | Biceps groove morphology + LHB and SSC tendinopathy |
| Shi et al. (2015) | IV | 94 | Sh pain | Imaging (MRI) vs arthroscopy (prospective) | LHBT subluxation on MRI was directly correlated with the severity of the SSC tendon tear (P < .001) under arthroscopy. | LHBT instability + SSC tear |
| Toshiaki et al. (2005) | V | 14 | Cadaveric Sh with and without and RC tears | LHBT dissection | Significant increase in LHBT cross-sectional area at the entrance of the biceps groove in the presence of RC tear compared to without (p=0.009). No substantial change in LHB (p=0.95) or SHB (p=0.3) cross-sectional area in the presence of RC tear compared to without. | Structural LHBT adaption in RC tears |
| Ulucakoy et al. (2021) | III | 200 | Sh pain:   - SSP rupture - SSC rupture - SLAP lesion | MRI + arthroscopic Sh surgery | Bicipital groove morphology + LHBT instability 34.5%. LHBT instability + SSP tendon rupture 72.5%, vs without 56.5% (p=0.027). LHBT instability + SLAP lesions 59.4%, vs without 45% (p=0.053). LHBT instability + SSC tendon rupture 52.2%, vs without 4.6% (p<0.001). | Biceps groove morphology + LHBT instability  LHBT instability + SSP tear  LHBT instability + SSC tear  LHBT instability + SLAP |
| Urita et al. (2016) | II | 55 | RC-related Sh pain | Arthroscopic RC surgery | Bicipital groove morphology (↓ width p=0.038, ↑ depth p=0.01 and increasing size of medial wall spur p < 0.0001 in the bicipital groove) were significantly associated with LHBT lesion SSC tear (p=0.004) was significantly associated with LHBT lesion. Pt’s with an SSC tendon tear was 6.29 times more likely to have a severe grade of LHBT lesion compared with those without (p=0.013). Pts with a medial spur in the bicipital groove are 10.9 times more likely to have a severe LHBT lesion than those without a medial spur (p =0.003). | Biceps groove morphology + LHBT lesion.  LHBT tear + SSC tear. |
| Uzel et al. (2011) | V | 1 | Cadaver Sh | Sh dissection | Bilateral spontaneous TD of the LHB with massive RC tear (SSP and ISP) and superior migration of the HOH associated with neo-articulation of the GHJ in a 103 yr. Cadaver. | LHB + RC tear (SSP + ISP) tendinopathy + age |
| Vestermark et al. (2018) | III | 27 | Acute proximal LHBT rupture (prospective) | MRI – presence or absence of RC tear | Observed RC disease on MRI in 93% of patients with acute LHBT rupture:   - SSP FTT (52%) - SSP PTT (48%) | Acute LHBT rupture + SSP tear |
| Walch et al. (1998) | IV | 71 | RC-related Sh pain (RC tear) + confirmed subluxation or dislocation of LHBT (retrospective) | Arthroscopic Sh surgery | LHBT pathology:   - LHBT instability (subluxation + dislocation) and isolated SSC RC tears (14%) - LHBT instability (subluxation + dislocation) and SSC + SSP RC tears (37%) - LHBT instability (subluxation + dislocation) and SSC, SSP and ISP RC tears (50%) - LHBT subluxation + 2 RC tear (SSC + SSP) = 68% - LHBT dislocation + 3 RC tear (SSC, SSP and ISP) = 70% | LHBT instability + RC tears |
| Yoon et al. (2018) | I | 432 | RC related Sh pain +/- SSC FTT | Imaging (MRA) vs arthroscopic RC surgery | Medial subluxation/dislocation of the LHBT on MRA (n=46) was highly predictive of a concurrent SSC full-thickness tear on arthroscopic examination (n=45).   - Accuracy (87%) - Sensitivity (45%) - Specificity (99%) - PPV (98%) - NPV (86%) | LHBT instability + SSC tear |
| Zabrzyński et al. (2018) | III | 28 | Sh pain + LHB tendinopathy | Arthroscopic Sh surgery  *Histopathological study of LHBT tissue samples | No relationship between the extent of neo-vascularisation of the LHBT and Sh pain. | Nil association between LHBT  neovascularization + Sh pain |

List of Abbreviations: Acromioclavicular Joint (ACJ); Anterosuperior impingement (ASI); Biceps Brachii (BB); Computed Tomographic Arthrography (CTA); Diagnostic Ultrasound (DUS); Flexion (Flex); Full Thickness Tear (FTT); Glenohumeral Joint (GHJ); Head of Humerus (HOH); Infraspinatus (ISP); Level of Evidence (LOE); Long Head of Biceps (LHB); Long Head of Biceps Tendon (LHBT); Magnetic Resonance Arthrography (MRA); Magnetic Resonance Imaging (MRI); Negative Predictive Value (NPV); Osteoarthritis (OA); Spearman’s Correlation Coefficient (*ρ); P-value (p); Patients (Pts); Patient Reported Outcome Measures (PROMS); Partial Thickness Tear (PTT); Positive Predictive Value (PPV); Pronation (Pro); Rotator Cuff (RC); Short Head of Biceps (SHB); Shoulder (Sh); Superior Labrum Anterior Posterior (SLAP); Subscapularis (SSC); Supination (Sup); Supraspinatus (SSP); Tenodesis (TD); Vascular Endothelial Growth Factor (VEGF).

References

1. Beall DP, Williamson EE, Ly JQ, Adkins MC, Emery RL, Jones TP, et al. Association of biceps tendon tears with rotator cuff abnormalities: degree of correlation with tears of the anterior and superior portions of the rotator cuff. AJR Am J Roentgenol. 2003;180(3):633-9.

2. Braun S, Horan MP, Elser F, Millett PJ. Lesions of the biceps pulley. Am J Sports Med. 2011;39(4):790-5.

3. Candela V, Preziosi Standoli J, Carbone S, Rionero M, Gumina S. Shoulder Long Head Biceps Tendon Pathology Is Associated With Increasing Rotator Cuff Tear Size. Arthrosc Sports Med Rehabil. 2021;3(5):e1517-e23.

4. Chae SH, Jung TW, Lee SH, Kim MJ, Park SM, Jung JY, et al. Hidden Long Head of the Biceps Tendon Instability and Concealed Intratendinous Subscapularis Tears. Orthop J Sports Med. 2020;8(1):2325967119898123.

5. Chen C-H, Hsu K-Y, Chen W-J, Shih C-H. Incidence and Severity of Biceps Long-Head Tendon Lesion in Patients with Complete Rotator Cuff Tears. Journal of Trauma and Acute Care Surgery. 2005;58(6):1189-93.

6. Chen CH, Chen CH, Chang CH, Su CI, Wang KC, Wang IC, et al. Classification and analysis of pathology of the long head of the biceps tendon in complete rotator cuff tears. Chang Gung Med J. 2012;35(3):263-70.

7. Deutch SR, Gelineck J, Johannsen HV, Sneppen O. Permanent disabilities in the displaced muscle from rupture of the long head tendon of the biceps. Scand J Med Sci Sports. 2005;15(3):159-62.

8. Dirkx G, Pouliart N. How reliable are imaging protocols in the diagnosis of subscapularis tears? Acta Orthop Belg. 2020;86(4):706-10.

9. Habermeyer P, Magosch P, Pritsch M, Scheibel MT, Lichtenberg S. Anterosuperior impingement of the shoulder as a result of pulley lesions: a prospective arthroscopic study. J Shoulder Elbow Surg. 2004;13(1):5-12.

10. Hanusch BC, Makaram N, Utrillas-Compaired A, Lawson-Smith MJ, Rangan A. Biceps sheath fluid on shoulder ultrasound as a predictor of rotator cuff tear: analysis of a consecutive cohort. J Shoulder Elbow Surg. 2016;25(10):1661-7.

11. Hawi N, Liodakis E, Garving C, Habermeyer P, Tauber M. Pulley lesions in rotator cuff tears: prevalence, etiology, and concomitant pathologies. Arch Orthop Trauma Surg. 2017;137(8):1097-105.

12. Ilahi OA, Labbe MR, Cosculluela P. Variants of the anterosuperior glenoid labrum and associated pathology. Arthroscopy. 2002;18(8):882-6.

13. Ilahi OA, Cosculluela PE, Ho DM. Classification of anterosuperior glenoid labrum variants and their association with shoulder pathology. Orthopedics. 2008;31(3):226.

14. Kim BR, Lee J, Ahn JM, Kang Y, Lee E, Lee JW, et al. Predicting the clinically significant subscapularis tendon tear: malposition and tear of the long head of the biceps tendon on shoulder magnetic resonance imaging. Acta Radiol. 2021;62(12):1648-56.

15. Lafosse L, Reiland Y, Baier GP, Toussaint B, Jost B. Anterior and posterior instability of the long head of the biceps tendon in rotator cuff tears: a new classification based on arthroscopic observations. Arthroscopy. 2007;23(1):73-80.

16. Lakemeier S, Reichelt JJ, Timmesfeld N, Fuchs-Winkelmann S, Paletta JR, Schofer MD. The relevance of long head biceps degeneration in the presence of rotator cuff tears. BMC Musculoskelet Disord. 2010;11:191.

17. Malavolta EA, Assuncao JH, Guglielmetti CL, de Souza FF, Gracitelli ME, Bordalo-Rodrigues M, et al. Accuracy of preoperative MRI in the diagnosis of subscapularis tears. Arch Orthop Trauma Surg. 2016;136(10):1425-30.

18. Mazzocca AD, McCarthy MB, Ledgard FA, Chowaniec DM, McKinnon WJ, Jr., Delaronde S, et al. Histomorphologic changes of the long head of the biceps tendon in common shoulder pathologies. Arthroscopy. 2013;29(6):972-81.

19. Mehta SK, Teefey SA, Middleton W, Steger-May K, Sefko JA, Keener JD. Prevalence and risk factors for development of subscapularis and biceps pathology in shoulders with degenerative rotator cuff disease: a prospective cohort evaluation. J Shoulder Elbow Surg. 2020;29(3):451-8.

20. Murthi AM, Vosburgh CL, Neviaser TJ. The incidence of pathologic changes of the long head of the biceps tendon. J Shoulder Elbow Surg. 2000;9(5):382-5.

21. Neviaser TJ, Neviaser RJ, Neviaser JS, Neviaser JS. The four-in-one arthroplasty for the painful arc syndrome. Clin Orthop Relat Res. 1982;163(163):107-12.

22. Ozer M, Kaptan AY, Ataoglu MB, Cetinkaya M, Ayanoglu T, Ince B, et al. The Buford complex: prevalence and relationship with labral pathologies. J Shoulder Elbow Surg. 2021;30(6):1356-61.

23. Petersson CJ. Spontaneous medial dislocation of the tendon of the long biceps brachii. An anatomic study of prevalence and pathomechanics. Clin Orthop Relat Res. 1986(211):224-7.

24. Redondo-Alonso L, Chamorro-Moriana G, Jimenez-Rejano JJ, Lopez-Tarrida P, Ridao-Fernandez C. Relationship between chronic pathologies of the supraspinatus tendon and the long head of the biceps tendon: systematic review. BMC Musculoskelet Disord. 2014;15:377.

25. Schmalzl J, Plumhoff P, Gilbert F, Gohlke F, Konrads C, Brunner U, et al. The inflamed biceps tendon as a pain generator in the shoulder: A histological and biomolecular analysis. J Orthop Surg (Hong Kong). 2019;27(1):2309499018820349.

26. Shah SH, Small KM, Sinz NJ, Higgins LD. Morphology of the Lesser Tuberosity and Intertubercular Groove in Patients With Arthroscopically Confirmed Subscapularis and Biceps Tendon Pathology. Arthroscopy. 2016;32(6):968-75.

27. Shi LL, Mullen MG, Freehill MT, Lin A, Warner JJ, Higgins LD. Accuracy of long head of the biceps subluxation as a predictor for subscapularis tears. Arthroscopy. 2015;31(4):615-9.

28. Toshiaki A, Itoi E, Minagawa H, Yamamoto N, Tuoheti Y, Seki N, et al. Cross-sectional area of the tendon and the muscle of the biceps brachii in shoulders with rotator cuff tears: a study of 14 cadaveric shoulders. Acta Orthop. 2005;76(4):509-12.

29. Ulucakoy C, Kaptan AY, Yapar A, Orhan O, Ozer M, Kanatli U. The effect of bicipital groove morphology on the stability of the biceps long head tendon. Arch Orthop Trauma Surg. 2021;141(8):1325-30.

30. Urita A, Funakoshi T, Amano T, Matsui Y, Kawamura D, Kameda Y, et al. Predictive factors of long head of the biceps tendon disorders-the bicipital groove morphology and subscapularis tendon tear. J Shoulder Elbow Surg. 2016;25(3):384-9.

31. Uzel AP, Bertino R, Boileau P. Bilateral spontaneous tenodesis of the long head of the biceps at the bicipital groove with massive rotator cuff tear on a 103-year-old female cadaver: the natural evolution of the human shoulder? Musculoskelet Surg. 2011;95 Suppl 1(S1):S79-82.

32. Vestermark GL, Van Doren BA, Connor PM, Fleischli JE, Piasecki DP, Hamid N. The prevalence of rotator cuff pathology in the setting of acute proximal biceps tendon rupture. J Shoulder Elbow Surg. 2018;27(7):1258-62.

33. Walch G, Nove-Josserand L, Boileau P, Levigne C. Subluxations and dislocations of the tendon of the long head of the biceps. J Shoulder Elbow Surg. 1998;7(2):100-8.

34. Yoon JS, Kim SJ, Choi YR, Lee W, Kim SH, Chun YM. Medial Subluxation or Dislocation of the Biceps on Magnetic Resonance Arthrography Is Reliably Correlated with Concurrent Subscapularis Full-Thickness Tears Confirmed Arthroscopically. Biomed Res Int. 2018;2018:2674061.

35. Zabrzynski J, Paczesny L, Lapaj L, Grzanka D, Szukalski J. Process of neovascularisation compared with pain intensity in tendinopathy of the long head of the biceps brachii tendon associated with concomitant shoulder disorders, after arthroscopic treatment. Microscopic evaluation supported by immunohistochemical. Folia Morphol (Warsz). 2018;77(2):378-85.
