# Supplementary material for: The long head of biceps at the shoulder: a scoping review
Source: BMC Musculoskelet Disord. 2023 Mar 28;24:232. doi: 10.1186/s12891-023-06346-5 (PMC10044783; doi:10.1186/s12891-023-06346-5)
Supplement: Supplementary file 11 — Supplementary Material 11 [file 12891_2023_6346_MOESM11_ESM.docx]

# Additional file 11: Supplementary Table 9_BMC.docx; Biceps Brachii activity with rotator cuff pathology and shoulder instability

| Author | LOE | Participants & pathology | Functional task | EMG | Results |
| --- | --- | --- | --- | --- | --- |
| Baek et al. (2021) | III | 21 Subjects with RC tendinopathy vs unaffected Sh. | Predictable and unpredictable ball (200g and 500g) drop/catch. | sEMG (BB, ISP, UT, AD). | Significantly delayed anticipatory muscle activation for ISP, UT, AD and BB between RC tendinopathy Sh vs. unaffected Sh during a predictable and unpredictable ball-drop trial, except for 500g predictable ball drop. |
| Glousman et al. (1988) | III | Fifteen throwing athletes with anterior Sh instability vs healthy controls. | Baseball pitching. | fwEMG:(BB, MD, SSP, ISP, PM, SSC, SA). | Hyperactivity of BB during pitching in subjects with anterior Sh instability. |
| Hawkes, Alizadehkhaiyat, Kemp, et al. (2012) | III | Eleven subjects with MRCT vs. healthy controls. | Modified FIT-HaNSA (a shelf-lifting task with 1kg weight). | sEMG: (AD, MD, PD, PM, UT, SA, LD, TM, Br and BB).  fwEMG: (SSP, ISP, SSC). | Hyperactivity of BB during Sh elevation task in subjects with MRCT. |
| Hawkes et al. (2014) | II | Eleven subjects with MRCT vs. healthy controls. | Submaximal grip task. | sEMG:(AD, MD, PD, PM, UT, SA, LD, TM, Br and BB).  fwEMG: (SSP, ISP and SSC). | No significant differences in BB activity or fatigue during a gripping task in patients with MRCT. |
| Kido et al. (1998) | III | Thirty-seven subjects with FTT RC tear vs. controls. | Sh elevation in scaption to 120° with and without a 1kg weight. | sEMG BB. | Hyperactivity of BB during Sh elevation task in subjects with FTT RC. |
| Kim et al. (2001) | III | Thirty-eight subjects with anterior Sh instability vs. contralateral Sh as control. | Static sh Abd (0°, 45°, 90°, and 120°) with ER + elbow brace. | fwEMG: (LHB and SSP). | Hyperactivity of LHB during simulated cocking phase of throwing (Sh Abd and ER) in subjects with anterior Sh instability. |
| E. J. D. Veen et al. (2021) | III | Twelve subjects with RC tear vs. healthy controls. | FIT-HaNSA (Function reaching tasks). | sEMG: (AD, MD, PD, PM, UT, LD, BB).  fwEMG (SSP, ISP, SSC). | Hyperactivity + pre-setting activity of BB during reaching ADLs in patients with RC tears. |
| Yamaguchi et al. (1997) | IV | Thirty subjects with RC tear (n=14) vs. healthy controls. | Active Sh ROM in scaption + elbow immobilisation brace. | sEMG: (BB, Br).  fwEMG: (SSP). | No significant differences in BB activity in patients with RC tear during Sh ROM with elbow immobilised in a brace. |

*List of Abbreviations: Abduction (Abd); Anterior Deltoid (AD); Biceps brachii (BB); Brachioradialis (Br); External Rotation (ER); Fine Wire Electromyography (fwEMG); Full Thickness Tear (FTT); Functional Impairment Test-Hand and Neck/Shoulder/Arm (FIT-HaNSA); Infraspinatus (ISP); Latissimus Dorsi (LD); Level of Evidence (LOE); Long Head of Biceps (LHB); Massive Rotator Cuff Tear (MRCT); Middle Deltoid (MD); Posterior Deltoid (PD); Pectoralis Major (PM); Range of Motion (ROM); Rotator cuff (RC); Serratus Anterior (SA); Shoulder (Sh); Surface Electromyography (sEMG); Subscapularis (SSC); Supraspinatus (SSP); Teres Major (TM); Upper trapezius (UT).*

References

1. Baek S, Ki SY, Chung SW, Lee SJ, Cho YC, Oh KS. Delayed Anticipatory Muscle Activation in Rotator Cuff Tendinopathy. Orthop J Sports Med. 2021;9(7):23259671211019360.

2. Glousman R, Jobe F, Tibone J, Moynes D, Antonelli D, Perry J. Dynamic electromyographic analysis of the throwing shoulder with glenohumeral instability. J Bone Joint Surg Am. 1988;70(2):220-6.

3. Hawkes DH, Alizadehkhaiyat O, Kemp GJ, Fisher AC, Roebuck MM, Frostick SP. Shoulder muscle activation and coordination in patients with a massive rotator cuff tear: an electromyographic study. J Orthop Res. 2012;30(7):1140-6.

4. Hawkes DH, Alizadehkhaiyat O, Kemp GJ, Fisher AC, Roebuck MM, Frostick SP. Electromyographic assessment of muscle fatigue in massive rotator cuff tear. J Electromyogr Kinesiol. 2015;25(1):93-9.

5. Kido T, Itoi E, Konno N, Sano A, Urayama M, Sato K. Electromyographic activities of the biceps during arm elevation in shoulders with rotator cuff tears. Acta Orthop Scand. 1998;69(6):575-9.

6. Kim SH, Ha KI, Kim HS, Kim SW. Electromyographic activity of the biceps brachii muscle in shoulders with anterior instability. Arthroscopy. 2001;17(8):864-8.

7. Veen EJD, Koorevaar CT, Verdonschot KHM, Sluijter TE, de Groot T, van der Hoeven JH, et al. Compensatory Movement Patterns Are Based on Abnormal Activity of the Biceps Brachii and Posterior Deltoid Muscles in Patients with Symptomatic Rotator Cuff Tears. Clin Orthop Relat Res. 2021;479(2):378-88.

8. Yamaguchi K, Riew KD, Galatz LM, Syme JA, Neviaser RJ. Biceps activity during shoulder motion: an electromyographic analysis. Clin Orthop Relat Res. 1997;336(336):122-9.
