# Supplementary material for: The long head of biceps at the shoulder: a scoping review
Source: BMC Musculoskelet Disord. 2023 Mar 28;24:232. doi: 10.1186/s12891-023-06346-5 (PMC10044783; doi:10.1186/s12891-023-06346-5)
Supplement: Supplementary file 12 — Supplementary Material 12 [file 12891_2023_6346_MOESM12_ESM.docx]

# Additional file 12: Supplementary Table 10_BMC.docx; Miscellaneous assessment findings for the LHB

| Author | LOE | No | Participants (Sh) | Outcomes | Reference standard | Results | Implications |
| --- | --- | --- | --- | --- | --- | --- | --- |
| Beer et al. (2021) | II | 116 | Sh pain (RC  tears, Bankart lesions, and SLAP lesions. | Preoperative Body Mass Index (BMI), Orthopaedic special tests (OST) and MRI findings. | Sh arthroscopy. | Overall specificity and sensitivity of SSP RC tears OST (p<0.001):   - Diagnostic sensitivity of Jobe/Empty can test was higher in overweight (83%) and obese patients (81%) compared with patients with a healthy BMI (78%). - Diagnostic specificity of Jobe/Empty can test was lower in obese patients (56%) compared with overweight (71%) and patients with a healthy BMI (73%). - Statistical significance of Jobe/Empty can in obese group (p = 0.058).   Overall specificity and sensitivity of Sh instability OST (p< 0.001):   - Diagnostic specificity of apprehension and relocation tests was higher in overweight (apprehension =100%; relocation tests = 100%) and obese (apprehension = 95%; relocation tests = 95%) patients compared with patient with a healthy BMI. - Diagnostic sensitivity of apprehension and relocation tests was higher in the overweight patients (apprehension =83% and relocation test = 75%) compared with patients with a healthy BMI.   MRI:   - Diagnostic interpretation and accuracy for detecting Sh pathology reduced as BMI increased (p<0.001). | BMI has a significant counterintuitive effect on the sensitivity and specificity of OST for RC pathology and Sh instability. |
| Eubank et al. (2021) | V | NA | Clinical decision-making tool for Sh pain in primary care (assessment,  diagnosis, management, and treatment of Sh pain). | Literature review (clinical  care pathways, algorithms, clinical practice guidelines, or  consensus guidelines)  Expert group consensus (medical, allied health and patient advocate). | NA | Clinical decision-making tool for Sh pain in primary care developed. Including:   - Clinical examination algorithm - Diagnostic imaging recommendations - Care pathways | Use of standardised clinical decision-making tool for Sh pain in primary care. |
| Kerschbaum et al. (2016) | II | 57 | Clinical signs of LHB  pathology on examination (Speed’s and O’Brien’s test). | Mean Long Head of Biceps (LHB) Score and mean  Constant score (CS). | Diagnostic arthroscopy + Group 1) TD (26), Group 2) TT (17) and Group 3) no further surgery (14). | Preoperatively, the total Long Head of Biceps Score and the total Constant Score showed no significant differences between the groups and were unable to distinguish between patients with arthroscopically proven LHB pathologies requiring TD (group 1) or TT (group 2) and patients with LHB symptoms and an absence of pathology not requiring surgery (group 3). | The LHB score and CS are unable to detect LHB pathologies preoperatively. |
| Lewis et al. (2016) | V | 11 | Unilateral Sh pain. | Response to Shoulder  Symptom Modification Procedure (SSMP):  1) no change  2) worse  3) partial improvement  4) complete improvement | Thirty-seven clinician’s video analyses of patients performing SSMP. | Inter-rater reliability (Krippendorff’s α):   - Moderate to high inter-rater reliability for the whole cohort of clinicians (α=0.762 - 0.928). - Moderate to substantial inter-rater reliability for clinicians participating in long and short training (α=0.733 to 1.000). | SSMP showed a reasonable degree of intertester reliability. |
| Meakins et al. (2018) | II | 26 | Sh pain. | Shoulder Symptom Modification Procedure  (SSMP). | Numeric Pain Rating  Scale (NPRS) + Shoulder Pain and Disability Index (SPADI). | Inter-rater reliability (Kappa κ):   - Moderate Inter-rater reliability (κ=0.47)   Correlations to Sh pain (Spearman’s r):   - Fair to poor association of within-session changes in Sh pain in the short term (r=0.24 - 0.01). - Poor association of within-session changes in Sh pain in the mid-term (r=−0.03). - Substantial to a moderate association of between-session changes in Sh pain ranging from (r= 0.74 – 0.47) in the short term but slight in the mid-term (r=0.22). | Insufficient evidence to recommend  SSMP is a reliable or validated tool with moderate inter-rater reliability and poor association with Sh pain. |
| Riley et al. (2020) | III | 113 | Sh pain. | Symptom modification IGR test cluster (impingement GIRD apprehension-relocation).  *GIRD - glenohumeral internal rotation deficit. | SPADI + response to individual component tests (Apprehension, Apprehension/relocation, GIRD) + IGR test cluster by two blinded testers. | Relevant inter-rater reliability (≥75%):   - Negative specific agreement percentages for GIRD and IGR test clusters were 81.7% and 90.4%. - Total agreement percentages for GIRD and IGR test clusters were 80.9% and 87.8%. - Positive specific agreement percentages for all component tests and IGR test cluster were <75%.   Statistically significant correlation (p-value) and meaningfulness (Phi ≥0.50 = moderate correlation):   - Statistically significant correlation (p ≥ 0.50) between GIRD and the IGR test cluster for both tester A (Phi= 0.71, p < .01) and Tester B (Phi=0.82, p < .01). | A negative IGR test might be a reliable assessment tool to exclude patients with Sh pain related to GIRD. |

*List of Abbreviations: Body Mass Index (BMI); Glenohumeral Internal Rotation Deficit (GIRD); Impingement GIRD Apprehension-Relocation (IGR); ); Kappa Coefficient; (K); Krippendorff’s Alpha Coefficient (α); Level of Evidence (LOE); Magnetic Resonance Imaging (MRI); Numeric Pain Rating Scale (NPRS); Orthopaedic Special Tests (OST); P-value (p); Phi Correlation (Phi); Rotator Cuff (RC); Shoulder (Sh); Shoulder Symptom Modification Procedure (SSMP); Spearman’s Rank Correlation Coefficient (r); Shoulder Pain and Disability Index (SPADI); Superior Labrum Anterior Posterior (SLAP); Supraspinatus (SSP); Tenodesis (TD); Tenotomy (TT).*

References

1. Beer Y, Gilat R, Ner EB, Shohat N, Atoun E, Lindner D, et al. Impact of Body Mass Index on the Accuracy of Physical Examination and MRI of the Shoulder. Orthop J Sports Med. 2021;9(2):2325967120985643.

2. Eubank BHF, Lackey SW, Slomp M, Werle JR, Kuntze C, Sheps DM. Consensus for a primary care clinical decision-making tool for assessing, diagnosing, and managing shoulder pain in Alberta, Canada. BMC Fam Pract. 2021;22(1):201.

3. Kerschbaum M, Arndt L, Bartsch M, Chen J, Gerhardt C, Scheibel M. Using the LHB score for assessment of LHB pathologies and LHB surgery: a prospective study. Arch Orthop Trauma Surg. 2016;136(4):469-75.

4. Lewis JS, McCreesh K, Barratt E, Hegedus EJ, Sim J. Inter-rater reliability of the Shoulder Symptom Modification Procedure in people with shoulder pain. BMJ Open Sport Exerc Med. 2016;2(1):e000181.

5. Meakins A, May S, Littlewood C. Reliability of the Shoulder Symptom Modification Procedure and association of within-session and between-session changes with functional outcomes. BMJ Open Sport Exerc Med. 2018;4(1):e000342.

6. Riley SP, Grimes JK, Apeldoorn AT, de Vet R. Agreement and reliability of a symptom modification test cluster for patients with subacromial pain syndrome. Physiother Res Int. 2020;25(3):e1842.
