# Supplementary material for: The long head of biceps at the shoulder: a scoping review
Source: BMC Musculoskelet Disord. 2023 Mar 28;24:232. doi: 10.1186/s12891-023-06346-5 (PMC10044783; doi:10.1186/s12891-023-06346-5)
Supplement: Supplementary file 13 — Supplementary Material 13 [file 12891_2023_6346_MOESM13_ESM.docx]

# Additional file 13: Supplementary Table 11_BMC.docx; Special tests for LHB tendinopathy (exclusive of SLAP/labrum pathology)

| OST | Author | LOE | No | Reference standard | Sens | Spec | NPV | PPV | LR+ | LR- |
| --- | --- | --- | --- | --- | --- | --- | --- | --- | --- | --- |
| Bicipital groove tenderness for LHB tendinopathy | Gill et al. (2007b) | II | 847 | Arthroscopic surgery. | 53% | 54% | 6% | 95% | 1.13 | NA |
|  | Rosas et al. (2017) | II | NA | Arthroscopy or Arthrotomy. | 57% | 72% | 57% | 72% | 2.07 | 0.59 |
| Obrien’s test for LHB tendinopathy | Arrigoni et al. (2014) | II | 109 | Arthroscopy | 66% | 46% | 81% | 28% | NA | NA |
|  | Ben Kibler et al. (2009) | II | 325 | Sh surgery | 38% | 61% | 31% | 67% | 0.96 | 1.02 |
| Speed’s test for LHB tendinopathy | Arrigoni et al. (2014) | II | 109 | Arthroscopy | 93% | 27% | 82% | 54% | NA | NA |
|  | Bélanger et al. (2019) | II | NA | Surgery (open or arthroscopy), HRUS, MRI or MRA. | 65% | 61% | NA | NA | 1.67 | 0.57 |
|  | Bennett (1998) | II | 45(46) | Arthroscopic surgery | 90% | 13.8% | 23% | 83% | NA | NA |
|  | Biederwolf (2013) | I | NA | Surgery observation + Ultrasonography. | 32%-90% | 14%-75% | NA | NA | 1.0-1.28 | 0.71-0.91 |
|  | Cardoso et al. (2019) | I | 65 | Arthroscopic surgery | Strict interpretation = 61%  Loose interpretation = 83% | Strict interpretation = 71%  Loose interpretation = 33% | Strict interpretation = 78%  Loose interpretation = 68% | Strict interpretation = 52%  Loose interpretation = 53% | Strict interpretation = 2.09  Loose interpretation = 1.24 | Strict interpretation = 0.55  Loose interpretation = 0.51 |
|  | Gill et al. (2007b) | II | 847 | Arthroscopic surgery | 50% | 67% | 8% | 96% | 1.51 | NA |
|  | Ben Kibler et al. (2009) | II | 325 | Sh surgery | 54% | 81% | 56% | 79% | 2.77 | 0.58 |
|  | Li et al. (2020) | III | 143 | Arthroscopy | Bicep’s injury = 49%  Pulley lesion = 44% | Bicep’s injury = 67%  Pulley lesion = 58% | Bicep’s injury = 67%  Pulley lesion = 49% | Bicep’s injury = 49%  Pulley lesion = 52% | Bicep’s injury = 1.48  Pulley lesion = 1.05 | Bicep’s injury = 0.76  Pulley lesion = 0.97 |
|  | Rosas et al. (2017) | II | NA | Arthroscopy or Arthrotomy | 54% | 81% | 56% | 79% | 2.77 | 0.58 |
| Yergason’s test for LHB tendinopathy | Bélanger et al. (2019) | II | NA | Surgery (open or arthroscopy), HRUS, MRI or MRA. | 41% | 84% | NA | NA | 2.56 | 0.70 |
|  | Biederwolf (2013) | I | NA | Surgery observation + Ultrasonography. | 43%-74% | 58%-79% | NA | NA | 1.76-2.05 | 0.45-0.72 |
|  | Cardoso et al. (2019) | I | 65 | Arthroscopic surgery | Strict interpretation = 37%  Loose interpretation = 46% | Strict interpretation = 83%  Loose interpretation = 54% | Strict interpretation = 79%  Loose interpretation = 63% | Strict interpretation = 43%  Loose interpretation = 37% | Strict interpretation = 2.20  Loose interpretation = 1.01 | Strict interpretation = 0.76  Loose interpretation = 0.99 |
|  | Ben Kibler et al. (2009) | II | 325 | Sh surgery | 41% | 79% | 48% | 74% | 1.94 | 0.74 |
|  | Li et al. (2020) | III | 143 | Arthroscopy | Bicep’s injury = 45%  Pulley lesion = 39% | Bicep’s injury = 62%  Pulley lesion = 55% | Bicep’s injury = 62%  Pulley lesion = 45% | Bicep’s injury = 45%  Pulley lesion = 49% | Bicep’s injury = 1.18  Pulley lesion = 0.86 | Bicep’s injury = 0.89  Pulley lesion = 1.11 |
|  | Rosas et al. (2017) | II | NA | Arthroscopy or Arthrotomy | 41% | 79% | 48% | 74% | 1.94 | 0.74 |
| Belly Press test for LHB tendinopathy | Gill et al. (2007b) | II | 847 | Arthroscopic surgery | 17% | 92% | 24% | 88% | 2.01 | NA |
|  | Ben Kibler et al. (2009) | II | 325 | Sh surgery | 31% | 85% | 50% | 72% | 2.10 | 0.81 |
|  | Rosas et al. (2017) | II | NA | Arthroscopy or Arthrotomy | 31% | 85% | 50% | 72% | 2.10 | 0.81 |
| Bear Hug test for LHB tendinopathy | Ben Kibler et al. (2009) | II | 325 | Sh surgery | 79% | 60% | 47% | 86% | 1.95 | 0.36 |
|  | Rosas et al. (2017) | II | NA | Arthroscopy or Arthrotomy | 79% | 60% | 47% | 86% | 1.95 | 0.36 |
| Uppercut test for LHB tendinopathy | Ben Kibler et al. (2009) | II | 325 | Sh surgery | 73% | 78% | 63% | 85% | 3.38 | 0.34 |
|  | Cardoso et al. (2019) | I | 65 | Arthroscopic surgery | Strict interpretation = 61%  Loose interpretation = 90% | Strict interpretation = 63%  Loose interpretation = 38% | Strict interpretation = 74%  Loose interpretation = 71% | Strict interpretation = 48%  Loose interpretation = 69% | Strict interpretation = 1.63  Loose interpretation = 1.44 | Strict interpretation = 0.62  Loose interpretation = 0.26 |
|  | Rosas et al. (2017) | II | NA | Arthroscopy or Arthrotomy | 73% | 78% | 63% | 85% | 3.38 | 0.34 |
| Backward Traction (BT) test for LHB tendinopathy  Biceps Resisted Flexion test for LHB tendinopathy | Li et al. (2020) | III | 143 | Arthroscopy | Bicep’s injury = 74%  Pulley lesion = 81% | Bicep’s injury = 60%  Pulley lesion = 61%  *Addition of irBT + erBT demonstrated higher specificity 79% and 73%, for the diagnosis of specific anteromedial and posterolateral pulley lesions, respectively | Bicep’s injury = 72%  Pulley lesion = 66% | Bicep’s injury = 62%  Pulley lesion = 78% | Bicep’s injury = 1.85  Pulley lesion = 2.07 | Bicep’s injury = 0.43  Pulley lesion = 0.31 |
|  | Arrigoni et al. (2014) | II | 109 | Arthroscopy | 60% | 88% | 95% | 39% | NA | NA |
| Gilcrest palm-up test for LHB tendinopathy | Biederwolf (2013) | I | NA | Surgery observation + Ultrasonography | 63%-74% | 35%-58% | NA | NA | 0.97-1.76 | 0.45-1.06 |
| Modified Biceps resisted Flex test for LHB tendinopathy. | Cardoso et al. (2019) | I | 65 | Arthroscopic surgery | Strict interpretation = 34%  Loose interpretation = 49% | Strict interpretation = 75%  Loose interpretation = 71% | Strict interpretation = 70%  Loose interpretation = 74% | Strict interpretation = 40%  Loose interpretation = 45% | Strict interpretation = 1.36  Loose interpretation = 1.67 | Strict interpretation = 0.88  Loose interpretation = 0.72 |

List of Abbreviations: Backward Traction (BT); Externally Rotated Backward Traction (erBT); Internally Rotated Backward Traction (irBT); Flexion (Flex); High-Resolution Ultrasound (HRUS); Level of Evidence (LOE); Long Head of Biceps (LHB); Magnetic Resonance Arthrography (MRA); Magnetic Resonance Imaging (MRI); Negative Likelihood Ratio (LR-); Negative Predictive Value (NPV); Not Applicable (NA); Positive Likelihood Ratio (LR+); Positive Predictive Value (PPV); Sensitivity (Sens); Shoulder (Sh); Specificity (Spec).

References

1. Gill HS, El Rassi G, Bahk MS, Castillo RC, McFarland EG. Physical examination for partial tears of the biceps tendon. American Journal of Sports Medicine. 2007;35(8):1334-40.

2. Rosas S, Krill MK, Amoo-Achampong K, Kwon K, Nwachukwu BU, McCormick F. A practical, evidence-based, comprehensive (PEC) physical examination for diagnosing pathology of the long head of the biceps. J Shoulder Elbow Surg. 2017;26(8):1484-92.

3. Arrigoni P, Ragone V, D'Ambrosi R, Denard P, Randelli F, Banfi G, et al. Improving the accuracy of the preoperative diagnosis of long head of the biceps pathology: the biceps resisted flexion test. Joints. 2014;2(2):54-8.

4. Ben Kibler W, Sciascia AD, Hester P, Dome D, Jacobs C. Clinical utility of traditional and new tests in the diagnosis of biceps tendon injuries and superior labrum anterior and posterior lesions in the shoulder. Am J Sports Med. 2009;37(9):1840-7.

5. Belanger V, Dupuis F, Leblond J, Roy JS. Accuracy of examination of the long head of the biceps tendon in the clinical setting: A systematic review. J Rehabil Med. 2019;51(7):479-91.

6. Bennett WF. Specificity of the Speed's test: arthroscopic technique for evaluating the biceps tendon at the level of the bicipital groove. Arthroscopy. 1998;14(8):789-96.

7. Biederwolf NE. A proposed evidence-based shoulder special testing examination algorithm: clinical utility based on a systematic review of the literature. Int J Sports Phys Ther. 2013;8(4):427-40.

8. Cardoso A, Amaro P, Barbosa L, Coelho AM, Alonso R, Pires L. Diagnostic accuracy of clinical tests directed to the long head of biceps tendon in a surgical population: a combination of old and new tests. J Shoulder Elbow Surg. 2019;28(12):2272-8.

9. Li D, Wang W, Liu Y, Ma X, Huang S, Qu Z. The backward traction test: a new and effective test for diagnosis of biceps and pulley lesions. J Shoulder Elbow Surg. 2020;29(2):e37-e44.
