# Supplementary material for: The long head of biceps at the shoulder: a scoping review
Source: BMC Musculoskelet Disord. 2023 Mar 28;24:232. doi: 10.1186/s12891-023-06346-5 (PMC10044783; doi:10.1186/s12891-023-06346-5)
Supplement: Supplementary file 14 — Supplementary Material 14 [file 12891_2023_6346_MOESM14_ESM.docx]

# Additional file 14: Supplementary Table 12_BMC.docx; Test clusters for LHB tendinopathy (exclusive of SLAP/labrum pathology)

| Author | LOE | No | Reference standard | Results |
| --- | --- | --- | --- | --- |
| Ben Kibler et al. (2009) | II | 325 | Sh surgery | A combination of the Uppercut and Speed’s tests was clinically more diagnostic at detecting biceps lesions (p = 0.021, R2 = 0.400) than either test in isolation (R = regression analysis). |
| Cardoso et al. (2019) | I | 65 | Arthroscopic surgery | Combination of Upper cut test (high Sens = 0.90; and low LR– = 0.26) be used as a screening tool to rule-out LHB tendinopathy and when the test is positive the Speed’s (high Spec = 0.71 and high LR+ = 2.09) and Yergason’s tests (high Spec = 0.83 and high LR+ = 2.20) should be used as confirmatory tests. |
| Rosas et al. (2017) | II | NA | Arthroscopy or Arthrotomy | A combination of the Uppercut test with LHB tenderness to palpation within the bicipital groove demonstrated the highest diagnostic accuracy of OST for LHB pathology with a reported Sens of 88% when performed in parallel and a Spec of 94% when performed in series. A combination of the uppercut test with DUS demonstrated the highest Sens of 97% when executed in parallel. A combination of DUS with either Speed’s, Yergason’s or the Uppercut test in series showed the highest Spec (100%) of all test combinations. |

List of Abbreviations: Diagnostic Ultrasound (DUS); Level of Evidence (LOE); Long Head of Biceps (LHB); Negative Likelihood Ratio (LR-); Orthopaedic Special Tests (OST); P-value (P); Positive Likelihood Ratio (LR+); Regression Analysis (R); Sensitivity (Sens); Shoulder (Sh); Specificity (Spec).

References

1. Ben Kibler W, Sciascia AD, Hester P, Dome D, Jacobs C. Clinical utility of traditional and new tests in the diagnosis of biceps tendon injuries and superior labrum anterior and posterior lesions in the shoulder. Am J Sports Med. 2009;37(9):1840-7.

2. Cardoso A, Amaro P, Barbosa L, Coelho AM, Alonso R, Pires L. Diagnostic accuracy of clinical tests directed to the long head of biceps tendon in a surgical population: a combination of old and new tests. J Shoulder Elbow Surg. 2019;28(12):2272-8.

3. Rosas S, Krill MK, Amoo-Achampong K, Kwon K, Nwachukwu BU, McCormick F. A practical, evidence-based, comprehensive (PEC) physical examination for diagnosing pathology of the long head of the biceps. J Shoulder Elbow Surg. 2017;26(8):1484-92.
