# Supplementary material for: The long head of biceps at the shoulder: a scoping review
Source: BMC Musculoskelet Disord. 2023 Mar 28;24:232. doi: 10.1186/s12891-023-06346-5 (PMC10044783; doi:10.1186/s12891-023-06346-5)
Supplement: Supplementary file 15 — Supplementary Material 15 [file 12891_2023_6346_MOESM15_ESM.docx]

# Additional file 15: Supplementary Table 13_BMC.docx; MRI and MRA for the diagnosis of LHB pathology

| Citation | LOE | No | Intervention | Reference standard | LHBT pathology | Accuracy | Sens | Spec | PPV | NPV | LR+ | LR- |
| --- | --- | --- | --- | --- | --- | --- | --- | --- | --- | --- | --- | --- |
| Banerjee et al. (2016) | IV | 55 | 1.5 & 3 Tesla MRI | Arthroscopy | Lesions of the  biceps tendon | 76.8%, 76.8%, 78.6% | 66.7%, 58.8%, 66.7% | 78.7%, 84.6%,  81.8% | - | Pooled 8.3 % | - | - |
| Baptista et al. (2019) | IV | 98 (100) | 1.5-Tesla MRI | Arthroscopy | Overall tear | 72%, 73% | 71%, 73% | 73%, 73% | 68%, 69% | 75%, 77% | - | - |
|  |  |  |  |  | FTT | 88%, 70% | 67%, 56% | 96%, 75% | 86%, 45% | 88%, 82% | - | - |
|  |  |  |  |  | Dislocation | 68%, 70% | 31%, 30% | 100%,100% | 100%,100% | 63%, 66% | - | - |
|  |  |  |  |  | Subluxation | 61%, 66% | 49%, 39% | 71%, 86% | 57%, 67% | 63%, 66% | - | - |
|  |  |  |  |  | Overall LHBT displacement | 64%, 70% | 58%,51% | 70%, 86% | 66%, 77% | 63%, 66% | - | - |
| De Maeseneer et al. (2011) | II | 36 | 3-Tesla MRA | Arthroscopy | PTT | - | 25%, 50% | 96%, 93% | - | - | - | - |
| Douglas P. Beall et al. (2003) | III | 111 | 1.5-Tesla MRI | Arthroscopy or open surgery | Biceps tear | 79% | 52% | 86% | NA | NA | - | - |
| Dubrow et al. (2014) | I | 66 | Non-contrast MRI | Arthroscopy | PTT | NA | 27.7% | 84.2% | 81.2% | 32.0% | - | - |
|  |  |  |  |  | Complete tear | NA | 56.3% | 98.0% | 90.0% | 87.5% | - | - |
| Genovese et al. (2013) | I | 42 | 1.5-Tesla MRA | Arthroscopy | Biceps lesions (n=3) | 100% | 100% | 100% | 100% | 100% | - | - |
| Groarke et al. (2021) | I | 200 | 3-Tesla MRI | Arthroscopy | LHBT  appearance | - | 41%–79% | 82%–97% | 52%–91% | 74%–93% | - | - |
|  |  |  |  |  | LHBT position | - | 57%–100% | 88%–99% | 51%–96% | 91%–100% | - | - |
|  |  |  | 3-Tesla MRA | Arthroscopy | LHBT  appearance | - | 36%-77% | 97%–100% | 79%–100% | 85%–96% | - | - |
|  |  |  |  |  | LHBT position | - | 71%–100% | 97%–100% | 71%–100% | 97%–100% | - | - |
| Kang et al. (2017) | II | 101 | 1.5 & 3 Tesla MRA | Arthroscopy | Subluxation of the LHBT  (Agreement 74% Kappa =0.44) | 72.9% (70/96)/ 84.4% (81/96) | 82.6% (19/23)/ 73.9% (17/23) | 69.9% (51/73)/  87.7% (64/73) | 46.3% (19/41)/ 65.4% (17/26) | 92.7% (51/55)/ 91.4% (64/70) | - | - |
| Kim et al. (2019) | II | 554 | 3-Tesla MRI | Arthroscopy | at least two abnormal signs.’ | 89.2% | 77.9% | 93.7% | 76.3% | 93.1% | - | - |
| Lee et al. (2016) | I | 80 | 3-Tesla MRI | Arthroscopy | PTT | 73.8%, 76.3% | 77.1%, 80% | 71.1%, 73.3% | 67.5%,70% | 67.5, 82.5% | - | - |
|  |  |  |  |  | Complete tear | 98.8%, 100% | 80%,100% | 100%, 100% | 100%, 100% | 98.7%, 100% | - | - |
| Loock et al. (2019) | I | 66 | 1.5-Tesla MRA | Arthroscopy | Static instability (neutral) | Highest values amongst x 3 assessors | 62% (95% C.I. 35–85) | 77% (95% C.I. 63–88) | 48% (95% C.I.26–70) | 86% (95% C.I. 72–95) | - | - |
|  |  |  |  |  | Dynamic instability (external rotation) |  | 50% (95% C.I. 29–71) | 62% (95% C.I. 46–77), | 44% (95% C.I. 25–65) | 68% (95% C.I. 50–82) | - | - |
|  |  |  |  |  | Tendinopathy |  | 49% (95% C.I. 36–62) | 100% (95% C.I. 3–100). | 100% (95% C.I. 89–100) | 3% (95% C.I. 0–16) | - | - |
|  |  |  |  |  | SLAP | 71.4%, 71.4%, 66.1% | 45%, 35%, 55% | 86.1%, 91.7%, 72.2% | - | Pooled 73.2 % | - | - |
|  |  |  |  |  | Complete tear | - | 0%, 0% | 94%, 94% | - | - | - | - |
| Malavolta et al. (2015) | III | 90 | 1.5-Tesla MRI | Arthroscopy | Bicep’s tear | - | 67% | 98% | 67% | 98% | 28 | 0.34 |
|  |  |  |  |  | Bicep’s instability | - | 53% | 72% | 30% | 87% | 1.88 | 0.65 |
| Mohtadi et al. (2004) | II | 58 | 1.5-Tesla MRI | Arthroscopy | Inflammation | - | 9.1% | 96.8% | 66.7% | 60.0% | - | - |
|  |  |  |  |  | PTT | - | 50% | 69.8% | 27.8% | 85.7% | - | - |
|  |  |  |  |  | Complete rupture | - | 0% | 94% | 0% | 94% | - | - |
| Nascimento and Claudio (2017) | II | 965 | 1.5-Tesla MRI | Arthroscopy | Lesions | 71% | 22% | 98% | 84% | 70% | 9.8 | 0.8 |
| Nourissat et al. (2014) | III | 38 | 1.5-Tesla MRI | Arthroscopy | Tendinopathy | - | 42.9% | 75% | 50% | 69.2% | - | - |
| Razmjou et al. (2016) | III | 183 (130 study vs 53 control) | 1.5-Tesla MRI | Arthroscopy | PTT | 57% | 27% | 86% | - | - | 2.03 | 0.84 |
|  |  |  |  |  | FTT | 76% | 54% | 98% | - | - | 25.85 | 0.47 |
|  |  |  |  |  | Subluxation or Dislocation | 92% | 100% | 83% | - | - | 6.0 | - |
| Christoph Schaeffeler et al. (2012) | II | 80 | 1.5 & 3 Tesla - MRA | Arthroscopy | Biceps Pulley Lesions | 94%, 94% and 85% | 89%, 86%, and 82% | 96%, 98%, and 87% | 64%, 77% and 31% | 99%, 99% and 98% | - | - |
|  |  |  |  |  | LHB Tendinopathy  (Oblique sagittal) | 85%, 91% and 78% | 93%,82% and 64% | 81%,96% and 85% |  |  |  |  |
|  |  |  |  |  | LHB Tendinopathy (transverse) | 84%, 80% and 71% | 75%, 43% and 21% | 89%,100% and 98% |  |  |  |  |
|  |  |  |  |  | LHB Subluxation | 78%, 81% and 85% | 36%, 50% and 64% | 100%, 98% and 96% | - | - | - | - |
| Tadros et al. (2015) | II | 199 | 1.5-Tesla MRI  (n=132) | Arthroscopy | Tendinosis | 64%, 61% | 18%, 36% | 79% 69% | 22% 28% | 74% 76% | - | - |
|  |  |  |  |  | PTT | 77%, 74% | 72%, 69% | 78%, 76% | 58%, 55% | 87%, 86% | - | - |
|  |  |  |  |  | All tears | 78%, 74% | 83%, 75% | 75%, 73% | 69%, 66% | 87%, 82% | - | - |
|  |  |  | 1.5-Tesla MRA (n=67) | Arthroscopy | Tendinosis | 81%, 70% | 38%, 15% | 91%, 83% | 50%, 18% | 86%, 80% | - | - |
|  |  |  |  |  | PTT | 88%, 79% | 70%, 60% | 91%, 82% | 58%, 38% | 95%, 92% | - | - |
|  |  |  |  |  | All tears | 88%, 79% | 73%, 64% | 91%, 82% | 62%, 41% | 94%, 92% | - | - |
|  |  |  |  |  | Degeneration | - | 20%, 20% | 97%, 84% | - | - | - | - |
|  |  |  |  |  | Dislocation | - | 66%, 0% | 94%, 100% | - | - | - | - |
|  |  |  |  |  | Pooled data | - | 27% | 94% | - | - | - | - |

List of Abbreviations: Diagnostic Ultrasound (DUS); Full Thickness Tear (FTT); Level of Evidence (LOE); Long Head of Biceps (LHB); Long Head of Biceps Tendon (LHBT); Magnetic Resonance Arthrography (MRA); Magnetic Resonance Imaging (MRI); Negative Likelihood Ratio (LR-); Negative Predictive Value (NPV); Partial Thickness Tear (PTT); Positive Likelihood Ratio (LR+); Positive Predictive Value (PPV); Sensitivity (Sens); Specificity (Spec); Superior Labrum Anterior Posterior (SLAP).

References

1. Banerjee M, Muller-Hubenthal J, Grimme S, Balke M, Bouillon B, Lefering R, et al. Moderate value of non-contrast magnetic resonance imaging after non-dislocating shoulder trauma. Knee Surg Sports Traumatol Arthrosc. 2016;24(6):1888-95.

2. Baptista E, Malavolta EA, Gracitelli MEC, Alvarenga D, Bordalo-Rodrigues M, Ferreira Neto AA, et al. Diagnostic accuracy of MRI for detection of tears and instability of proximal long head of biceps tendon: an evaluation of 100 shoulders compared with arthroscopy. Skeletal Radiol. 2019;48(11):1723-33.

3. De Maeseneer M, Boulet C, Pouliart N, Kichouh M, Buls N, Verhelle F, et al. Assessment of the long head of the biceps tendon of the shoulder with 3T magnetic resonance arthrography and CT arthrography. Eur J Radiol. 2012;81(5):934-9.

4. Beall DP, Williamson EE, Ly JQ, Adkins MC, Emery RL, Jones TP, et al. Association of biceps tendon tears with rotator cuff abnormalities: Degree of correlation with tears of the anterior and superior portions of the rotator cuff. American journal of roentgenology (1976). 2003;180(3):633-9.

5. Dubrow SA, Streit JJ, Shishani Y, Robbin MR, Gobezie R. Diagnostic accuracy in detecting tears in the proximal biceps tendon using standard nonenhancing shoulder MRI. Open Access J Sports Med. 2014;5:81-7.

6. Genovese E, Spano E, Castagna A, Leonardi A, Angeretti MG, Callegari L, et al. MR-arthrography in superior instability of the shoulder: correlation with arthroscopy. Radiol Med. 2013;118(6):1022-33.

7. Groarke P, Jagernauth S, Peters SE, Manzanero S, O'Connell P, Cowderoy G, et al. Correlation of magnetic resonance and arthroscopy in the diagnosis of shoulder injury. ANZ J Surg. 2021;91(10):2145-52.

8. Kang Y, Lee JW, Ahn JM, Lee E, Kang HS. Instability of the long head of the biceps tendon in patients with rotator cuff tear: evaluation on magnetic resonance arthrography of the shoulder with arthroscopic correlation. Skeletal Radiol. 2017;46(10):1335-42.

9. Kim JY, Rhee SM, Rhee YG. Accuracy of MRI in diagnosing intra-articular pathology of the long head of the biceps tendon: results with a large cohort of patients. BMC Musculoskelet Disord. 2019;20(1):270.

10. Lee RW, Choi SJ, Lee MH, Ahn JH, Shin DR, Kang CH, et al. Diagnostic accuracy of 3T conventional shoulder MRI in the detection of the long head of the biceps tendon tears associated with rotator cuff tendon tears. Skeletal Radiol. 2016;45(12):1705-15.

11. Loock E, Michelet A, D'Utruy A, Molinazzi P, Hannink G, Bertiaux S, et al. Magnetic resonance arthrography is insufficiently accurate to diagnose biceps lesions prior to rotator cuff repair. Knee Surg Sports Traumatol Arthrosc. 2019;27(12):3970-8.

12. Malavolta EA, Assuncao JH, Guglielmetti CL, de Souza FF, Gracitelli ME, Ferreira Neto AA. Accuracy of preoperative MRI in the diagnosis of disorders of the long head of the biceps tendon. Eur J Radiol. 2015;84(11):2250-4.

13. Mohtadi NG, Vellet AD, Clark ML, Hollinshead RM, Sasyniuk TM, Fick GH, et al. A prospective, double-blind comparison of magnetic resonance imaging and arthroscopy in the evaluation of patients presenting with shoulder pain. J Shoulder Elbow Surg. 2004;13(3):258-65.

14. Nascimento AT, Claudio GK. Magnetic resonance imaging without contrast as a diagnostic method for partial injury of the long head of the biceps tendon. Rev Bras Ortop. 2017;52(1):40-5.

15. Nourissat G, Tribot-Laspiere Q, Aim F, Radier C. Contribution of MRI and CT arthrography to the diagnosis of intra-articular tendinopathy of the long head of the biceps. Orthop Traumatol Surg Res. 2014;100(8 Suppl):S391-4.

16. Razmjou H, Fournier-Gosselin S, Christakis M, Pennings A, ElMaraghy A, Holtby R. Accuracy of magnetic resonance imaging in detecting biceps pathology in patients with rotator cuff disorders: comparison with arthroscopy. J Shoulder Elbow Surg. 2016;25(1):38-44.

17. Schaeffeler C, Waldt S, Holzapfel K, Kirchhoff C, Jungmann PM, Wolf P, et al. Lesions of the biceps pulley: diagnostic accuracy of MR arthrography of the shoulder and evaluation of previously described and new diagnostic signs. Radiology. 2012;264(2):504-13.

18. Tadros AS, Huang BK, Wymore L, Hoenecke H, Fronek J, Chang EY. Long head of the biceps brachii tendon: unenhanced MRI versus direct MR arthrography. Skeletal Radiol. 2015;44(9):1263-72.
