# Supplementary material for: The long head of biceps at the shoulder: a scoping review
Source: BMC Musculoskelet Disord. 2023 Mar 28;24:232. doi: 10.1186/s12891-023-06346-5 (PMC10044783; doi:10.1186/s12891-023-06346-5)
Supplement: Supplementary file 16 — Supplementary Material 16 [file 12891_2023_6346_MOESM16_ESM.docx]

# Additional file 16: Supplementary Table 14_BMC.docx; Ultrasound and injection for the diagnosis of LHB pathology

| Author | LOL | No (Sh) | Intervention | Reference standard | LHBT pathology | Accuracy | Sens | Spec | PPV | NPV | LR+ | LR- |
| --- | --- | --- | --- | --- | --- | --- | --- | --- | --- | --- | --- | --- |
| Ardic et al. (2006) | IV | 58 (59) | DUS | MRI | All biceps pathology | 100% | 100% | 100% | 100% | 100% | - | - |
| Armstrong et al. (2006) | II | 71 | DUS | Arthroscopy | FTT | - | 91% | 100% | 64% | 100% | - | - |
|  |  |  |  |  | PTT | - | 50% | 100% | 100% | 71% | - | - |
|  |  |  |  |  | Subluxation | - | 100% | 96% | 66% | 100% | - | - |
| Bélanger et al. (2019) | II | NA | High-resolution ultrasound (HRUS) | Arthroscopy or open surgery | Dislocation | - | 76% | 98% | - | - | 38% | 0.24% |
|  |  |  |  |  | Complete rupture | - | 71% | 98% | - | - | 35.5% | 0.30% |
| Fischer et al. (2015) | I | 45 | High-resolution ultrasound (HRU) | MRI | All biceps pathology | 86.7% | 95% | 80% | - | - | - | - |
|  |  |  |  |  | Dislocation | - | 100% | 97% | - | - | - | - |
|  |  |  |  |  | Complete rupture | - | 100% | 87% | - | - | - | - |
|  |  |  |  |  | Tenovaginitis | - | 58% | 63% | - | - | - | - |
| Huang and Wang (2013) | II | 336 | DUS | Nil reference | Tendinitis | - | Transverse view = 68%  Longitudinal view = 81% | Transverse view = 90%  Longitudinal view = 73% | - | - | - | - |
| Huguet et al. (2017) | III | 129 | MRI v DUS | Arthroscopy | MRI – LHBT pathology | - | 52% | 96% | 90% | 73% | - | - |
|  |  |  |  |  | DUS - Proximal LHBT pathology | - | 59% | 88% | 77% | 77% | - | - |
|  |  |  |  |  | DUS - Distal LHBT pathology (biceps groove) | - | 29% | 96% | 83% | 67% | - | - |
| Read and Perko (1998) | I | 42 | DUS | Arthroscopy or open surgery | Tendinitis | 95% | 80% | 100% | 100% | 94% | - | - |
|  |  |  |  |  | Dislocation | 100% | 100% | 100% | 100% | 100% |  |  |
|  |  |  |  |  | Rupture | 75% | 75% | 100% | 100% | 97% |  |  |
| Rosas et al. (2017) | II | NA | Diagnostic injection | Arthroscopy or arthrotomy | Biceps sheath | - | 66% | 87.5% | 60% | 90% | 5.33 | 0.38 |
| Skendzel et al. (2011) | I | 66 | DUS | Arthroscopy | FTT | 88% | 88% | 98% | 88% | 98% | - | - |
|  |  |  |  |  | PTT | 97% | 27% | 100% | 100% | 88% | - | - |
| Teefey et al. (2000) | IV | 98 (100) | DUS | Arthroscopy | Dislocation | - | 83.3% | 100% | 100% | 98.9% | - | - |
|  |  |  |  |  | Rupture | - | 63.6% | 98.9% | 87.5% | 95.7% | - | - |

List of Abbreviations: Diagnostic Ultrasound (DUS); Full Thickness Tear (FTT); High-Resolution Ultrasound (HRUS); Level of Evidence (LOE); Long Head of Biceps (LHB); Long Head of Biceps Tendon (LHBT); Magnetic Resonance Arthrography (MRA); Magnetic Resonance Imaging (MRI); Negative Likelihood Ratio (LR-); Negative Predictive Value (NPV); Partial Thickness Tear (PTT); Positive Likelihood Ratio (LR+); Positive Predictive Value (PPV); Sensitivity (Sens); Specificity (Spec); Superior Labrum Anterior Posterior (SLAP).

References

1. Ardic F, Kahraman Y, Kacar M, Kahraman MC, Findikoglu G, Yorgancioglu ZR. Shoulder impingement syndrome: relationships between clinical, functional, and radiologic findings. Am J Phys Med Rehabil. 2006;85(1):53-60.

2. Armstrong A, Teefey SA, Wu T, Clark AM, Middleton WD, Yamaguchi K, et al. The efficacy of ultrasound in the diagnosis of long head of the biceps tendon pathology. J Shoulder Elbow Surg. 2006;15(1):7-11.

3. Belanger V, Dupuis F, Leblond J, Roy JS. Accuracy of examination of the long head of the biceps tendon in the clinical setting: A systematic review. J Rehabil Med. 2019;51(7):479-91.

4. Fischer CA, Weber MA, Neubecker C, Bruckner T, Tanner M, Zeifang F. Ultrasound vs. MRI in the assessment of rotator cuff structure prior to shoulder arthroplasty. J Orthop. 2015;12(1):23-30.

5. Huang SW, Wang WT. Quantitative diagnostic method for biceps long head tendinitis by using ultrasound. ScientificWorldJournal. 2013;2013:948323.

6. Huguet D, Darrieutort-Laffite C, Le Goff B. Comparison of ultrasound and MRI in the diagnosis of proximal and distal biceps tendon pathology. Annals of the Rheumatic Diseases. 2017;76:731.

7. Read JW, Perko M. Shoulder ultrasound: diagnostic accuracy for impingement syndrome, rotator cuff tear, and biceps tendon pathology. J Shoulder Elbow Surg. 1998;7(3):264-71.

8. Rosas S, Krill MK, Amoo-Achampong K, Kwon K, Nwachukwu BU, McCormick F. A practical, evidence-based, comprehensive (PEC) physical examination for diagnosing pathology of the long head of the biceps. J Shoulder Elbow Surg. 2017;26(8):1484-92.

9. Skendzel JG, Jacobson JA, Carpenter JE, Miller BS. Long head of biceps brachii tendon evaluation: accuracy of preoperative ultrasound. AJR Am J Roentgenol. 2011;197(4):942-8.

10. Teefey SA, Hasan SA, Middleton WD, Patel M, Wright RW, Yamaguchi K. Ultrasonography of the rotator cuff. A comparison of ultrasonographic and arthroscopic findings in one hundred consecutive cases. J Bone Joint Surg Am. 2000;82(4):498-504.
