# Supplementary material for: The long head of biceps at the shoulder: a scoping review
Source: BMC Musculoskelet Disord. 2023 Mar 28;24:232. doi: 10.1186/s12891-023-06346-5 (PMC10044783; doi:10.1186/s12891-023-06346-5)
Supplement: Supplementary file 17 — Supplementary Material 17 [file 12891_2023_6346_MOESM17_ESM.docx]

# Additional file 17: Supplementary Table 15_BMC.docx; Arthroscopy for the diagnosis of LHB pathology

| Author | LOE | No | Participants | Intervention | Results | Implications |
| --- | --- | --- | --- | --- | --- | --- |
| Castagna et al. (2007) | IV | 150 | LHBT instability | Intra-articular Sh arthroscopy  /TD/TT. | LHBT dislocation n=92 (61%) In all 92 patients with a dislocated LHBT, a biceps pulley lesion and chondral print lesion at the level of the humeral head were found. LHBT subluxation n = 58 (39%). In all 58 patients with a subluxed LHBT, a pulley lesion was found in 49 (84%) patients and a chondral print at the level of the humeral head in 52 (89%) patients. In 9 (15%) of patients with equivocal biceps pulley lesions, the presence of a chondral print at the level of the humeral head led to a diagnosis of LHBT instability. | The chondral print sign on the HOH is a useful arthroscopic sign to identify LHB instability. |
| Festa et al. (2014) | V | 10 | Cadaveric Sh specimens | Intra-articular Sh arthroscopy/hook probe. | Use of hook probe during Sh arthroscopy visualised an additional 1.9 cm (mean) of extra-articular LHBT, representing:   - 30.8% (mean) of the LHBT extra-articular segment, 47.7% (mean) within the biceps groove - 76.3% (mean) of the extra-articular portion lying beneath the area from the pulley entrance to the distal edge of the transverse humeral ligament tendon. | Arthroscopic examination of the LHBT incompletely visualises the extra-articular portion of the LHBT. |
| Gilmer et al. (2015) | II | 62 | Open TD | Intra-articular Sh arthroscopy vs open surgical inspection. | Sh arthroscopy visualised only 32% of the LHBT with a statistically significant difference in the observed length of the LHBT during open versus arthroscopic examination (P<0.0001). Sh arthroscopy visualised only 67% of LHBT pathology, underestimating the reported pathology in 56% of patients and overestimating perceived pathology in 11% of patients. | Arthroscopic examination of the  LHB visualises only 32% of the LHBT and may underestimate pathology. |
| Grassbaugh et al. (2017) | III | 55 | Arthroscopy for Sh pain | Diagnostic arthroscopy to detect LHB tendinopathy and Lipstick sign.  *Lipstick sign - arthroscopic finding of an inflamed and  hyperemic LHBT within the bicipital groove. | Diagnostic sensitivity and specificity of arthroscopy for identifying LHB pathology:   - LHB tendinopathy – overall sensitivity and specificity were 49% and 67%, respectively - LHBT erythema – overall sensitivity and specificity were 64% and 32%, respectively   Interobserver reliability:   - The nonweighted κ score ranged from 0.042 to 0.419 (mean, 0.215 ± 0.116) for tendinopathy and from 0.486 to 0.835 (mean, 0.680 ± 0.102) for erythema   Intraobserver reliability:   - The nonweighted κ score ranged from 0.264 to 0.854 (mean, 0.615) for tendinopathy and from 0.641 to 0.951 (mean, 0.783) for erythema | Arthroscopic examination of LHB tendinopathy and erythema (Lipstick sign) demonstrated moderate sensitivity and specificity. |
| Jordan and Saithna (2017) | IV | 593 | 18 cadaver specimen dissection.  575 patients undergoing arthroscopy. | Arthroscopy to detect LHBT pathology. | In cadaver studies using a hook probe, arthroscopy visualised between 34% and 48% of the overall length of the LHBT. In clinical arthroscopy studies, the rate of missed diagnoses varied between 33% and 49% compared to open exploration. | Arthroscopic examination of the LHBT incompletely visualises the extra-articular portion of the LHBT, and the absence of LHBT pathology of arthroscopy cannot exclude an LHBT lesion. |
| Khil et al. (2017) | II | 65 | Sh pain undergoing Sh MRI and  arthroscopic surgery. | MRI finding of LHBT subluxation and detour sign compared with arthroscopy  Detour sign - anteriorly displaced LHBT coursing along the anteromedial cortical margin of the humeral head on axial MRI. | High correlation of detour sign on MRI and LHBT subluxation under arthroscopy (p<0.001). High correlation of detour sign on MRI and LHBT mucoid degeneration under arthroscopy (p<0.001). Significant correlation of detour sign on MRI and SSC tendon tears under arthroscopy (p<0.001). Significant correlation between LHBT subluxation and SSC tendon tears (p<0.001) and SSP tendon tears (p=0.02) under arthroscopy. | The detour sign on MRI may be a useful sign of LHBT subluxation associated with RC pathology. |
| Moon et al. (2015) | IV | 36 | Sh pain undergoing biceps TD with intra-articular biceps tears. | Arthroscopic tenodesis  Morphological and Histological analysis of LHBT tissue samples. | In approximately 80% of the intraarticular LHBT tears evaluated, an occult biceps lesion was observed going beyond the bicipital groove and extending to the distal extra-articular portion. | Proximal intra-articular LHBT tears may be associated with occult bicep lesions distally beyond the biceps groove. |
| Motley et al. (2018) | IV | NA | NA | Intra-articular arthroscopy examination of ramp test (manipulation of LHBT to identify SGHL, coracohumeral ligament, SSC tendon and subluxation and Instability of the LHBT). | The use of the ramp test to identify SGHL integrity, intra-articular subluxation, and instability of the LHB should be routinely included in diagnostic arthroscopy of the Sh. Intra-articular biceps tendon instability occurs due to SGHL tearing, leading to upper SSC RC partial tears or complete tears either acutely or over time. | Using a ramp test under arthroscopy may help identify SGHL integrity, intra-articular subluxation, and instability of the LHBT associated with SSC RC tears. |
| Sahu et al. (2016) | III | 330 | Arthroscopic RC repair surgery. | Video analysis of arthroscopic surgery to detect:  A Sentinel sign is defined as scuffing, abrasion, or a partial tear of the biceps tendon. | 79 of 330 videos showed the presence of SSC rupture. Of the 79 SSC tears observed, a sentinel sign was present in 61 patents. Ten patients with an intact biceps pulley and a positive sentinel sign were observed to have a hidden SSC tendon tear after removal of the anterior part of the biceps sling. | The presence of a sentinel sign of the LHBT under arthroscopy may indicate the presence of an occult SSC tendon tear. |
| Taylor et al. (2015) | IV | 285 | Eight cadaver specimen dissections.  277 patients undergoing arthroscopy. | Diagnostic Sh arthroscopy. | Arthroscopy failed to evaluate the biceps-labral complex fully,  Arthroscopy visualised only 78% of the LHBT relative to the distal margin of the SSC tendon. Arthroscopy visualised only 55% of the LHBT relative to the proximal border of the PM tendon. Arthroscopy failed to identify extra-articular bicipital tunnel lesions that were present and concealed in 47% of chronically symptomatic patients. 45% of patients with junctional lesions also had hidden bicipital tunnel lesions. 18% of patients had an offending lesion in the bicipital tunnel. | Arthroscopy fails too fully  evaluate the intra-articular biceps-labral complex and extra-articular bicipital tunnel lesions associated with pathology. |

List of Abbreviations: Kappa (K); Head of Humerus (HOH); Level of Evidence (LOE); Long Head of Biceps (LHB); Long Head of Biceps Tendon (LHBT); Magnetic Resonance Imaging (MRI); Not Applicable (NA); Pectoralis Major (PM); Rotator Cuff (RC); Shoulder (Sh); Subscapularis (SSC); Supraspinatus (SSP); Superior Glenohumeral Ligament (SGHL); Tenodesis (TD); Tenotomy (TT).

References

1. Castagna A, Mouhsine E, Conti M, Vinci E, Borroni M, Giardella A, et al. Chondral print on humeral head: an indirect sign of long head biceps tendon instability. Knee Surg Sports Traumatol Arthrosc. 2007;15(5):645-8.

2. Festa A, Allert J, Issa K, Tasto JP, Myer JJ. Visualization of the extra-articular portion of the long head of the biceps tendon during intra-articular shoulder arthroscopy. Arthroscopy. 2014;30(11):1413-7.

3. Gilmer BB, DeMers AM, Guerrero D, Reid JB, 3rd, Lubowitz JH, Guttmann D. Arthroscopic versus open comparison of long head of biceps tendon visualization and pathology in patients requiring tenodesis. Arthroscopy. 2015;31(1):29-34.

4. Grassbaugh JA, Bean BR, Greenhouse AR, Yu HH, Arrington ED, Friedman RJ, et al. Refuting the lipstick sign. J Shoulder Elbow Surg. 2017;26(8):1416-22.

5. Jordan RW, Saithna A. Physical examination tests and imaging studies based on arthroscopic assessment of the long head of biceps tendon are invalid. Knee Surg Sports Traumatol Arthrosc. 2017;25(10):3229-36.

6. Khil EK, Cha JG, Yi JS, Kim HJ, Min KD, Yoon YC, et al. Detour sign in the diagnosis of subluxation of the long head of the biceps tendon with arthroscopic correlation. Br J Radiol. 2017;90(1070):20160375.

7. Moon SC, Cho NS, Rhee YG. Analysis of "hidden lesions" of the extra-articular biceps after subpectoral biceps tenodesis: the subpectoral portion as the optimal tenodesis site. Am J Sports Med. 2015;43(1):63-8.

8. Motley GS, Guengerich B, Schuller T, Turbyfill A. The Ramp Test: An Arthroscopic Technique for Confirming Intra-articular Subluxation and Instability of the Long Head of the Biceps Tendon Within the Shoulder. Arthrosc Tech. 2018;7(4):e327-e30.

9. Sahu D, Fullick R, Giannakos A, Lafosse L. Sentinel sign: a sign of biceps tendon which indicates the presence of subscapularis tendon rupture. Knee Surg Sports Traumatol Arthrosc. 2016;24(12):3745-9.

10. Taylor SA, Khair MM, Gulotta LV, Pearle AD, Baret NJ, Newman AM, et al. Diagnostic glenohumeral arthroscopy fails to fully evaluate the biceps-labral complex. Arthroscopy. 2015;31(2):215-24.
