# Supplementary material for: The long head of biceps at the shoulder: a scoping review
Source: BMC Musculoskelet Disord. 2023 Mar 28;24:232. doi: 10.1186/s12891-023-06346-5 (PMC10044783; doi:10.1186/s12891-023-06346-5)
Supplement: Supplementary file 18 — Supplementary Material 18 [file 12891_2023_6346_MOESM18_ESM.docx]

# Additional file 18: Supplementary Table 16_BMC.docx; Nonsurgical management

| Author | LOE | No | Participants/Intervention | Clinical outcomes | Results | Implications |
| --- | --- | --- | --- | --- | --- | --- |
| Borms et al. (2017) | IV | 30 | Healthy volunteers (asymptomatic) | sEMG (%MVIC) of six Sh muscles, including BB, during 11 biceps targeted rehabilitation exercises. | Top seven exercises for BB activity from least active to most active (%MVIC); Pull-up in Pro with cord (24.06%), Air punch in Sh Flex 90° (27.58%), Dumbbell Sh Flex in ER and forearm Sup (35.56%), Pull-up in Sup with cord (41.96%), Incline dumbbell biceps curl (43.99%), Underarm throwing Sh Flex in 90º with soft weight ball (56.96%), Reverse punch with elastic tubing (67.37%). | BB activity is most active in the sagittal plane, during unsupported elbow Flex and  Sup, from an extended position (Sh E) OR during high-velocity explosive exercises such as underarm throwing. |
| Cools et al. (2014) | IV | 32 | Healthy volunteers (asymptomatic) | sEMG (%MVIC) activity of eight Sh muscles, including BB, during 16 common Sh exercises, including three biceps targeted rehabilitation exercises - Low activity (< 20% MVIC), Moderate activity (20 – 50% MVIC), High activity (>50% MVIC). | Top five exercises for BB activity from least active to most active (%MVIC); Pulley forearm Sup (Low - 15.4%), Dumbbell uppercut (Low - 19.3%), Dumbbell full can (Mod - 29.0%), Pulley elbow Flex in forearm Sup (Mod - 34.6%), Dumbbell forward Flex in Sh ER and forearm Sup (Mod - 35.9%). | BB is most active during elbow Flex, forearm Sup and Sh elevation in the sagittal and scapular planes. |
| Eubank et al. (2021) | V | NA | Patients with proximal biceps pathology | Rapid literature review of evidence-based clinical decision-making tools for Sh pain.  Establishment of consensus for the assessment diagnosis and management of patients with Sh pain. | Recommendations in primary care:   - Analgesic/NSAIDS for swelling and pain control. Radiological intervention (X-ray - standard Sh series). Image-guided steroid injection. - Early conservative management and exercise-based therapy for at least 12 weeks before a specialist opinion are sought. - Inappropriate referral and additional diagnostic imaging (MRI/DUS) cost. - Referrals for eligible patients to complementary allied health models of care pathways. - Care is provided under the biopsychosocial framework, including access and advocacy for surgical care when required. | Clinical decision tool and care pathway. |
| McDevitt et al. (2020) | IV | 10 | Patients with LHB tendinopathy: Dry needling (DN) to the LHBT, Eccentric, concentric exercise (ECE) program, LHBT stretching. | GROC; NPRS; Q-DASH  *ECE (In supine - dumbbell concentric elbow Flex and eccentric elbow E performed in full Sh E).  * LHBT stretching (Sh E, elbow E and forearm Pro). | A significant change in clinical outcome scores was observed from initial assessment to discharge; NPRS mean improvement of 3.9 (p<0.001), Q-DASH mean improvement of 19.01% (p<0.02), and patients perceived recovery GROC +5.4 (SD, 1.3). | Patients with chronic LHB tendinopathy pain and disability may respond to DN to the LHBT, loaded ECE exercise and static BB stretching. |

List of Abbreviations: Biceps Brachii (BB); Diagnostic Ultrasound (DUS); Dry Needling(DN); Eccentric Concentric Exercise (ECE); External Rotation (ER); Extension (E); Flexion (Flex); Global Rating of Change (GROC); Level of Evidence (LOE); Long Head of Biceps (LHB); Long Head of Biceps Tendon (LHBT); Magnetic Resonance Imaging (MRI); Maximal Voluntary Isometric Contraction (MVIC); Numeric Pain Rating Scale (NPRS); P-value (p); Pronation (Pro); Quick - Disabilities of the Arm, Shoulder and Hand (Q-DASH); Shoulder (Sh); Supination (Sup); Surface Electromyography (sEMG).

References

1. Borms D, Ackerman I, Smets P, Van den Berge G, Cools AM. Biceps Disorder Rehabilitation for the Athlete: A Continuum of Moderate- to High-Load Exercises. Am J Sports Med. 2017;45(3):642-50.

2. Cools AM, Borms D, Cottens S, Himpe M, Meersdom S, Cagnie B. Rehabilitation Exercises for Athletes With Biceps Disorders and SLAP Lesions: A Continuum of Exercises With Increasing Loads on the Biceps. Am J Sports Med. 2014;42(6):1315-22.

3. Eubank BHF, Lackey SW, Slomp M, Werle JR, Kuntze C, Sheps DM. Consensus for a primary care clinical decision-making tool for assessing, diagnosing, and managing shoulder pain in Alberta, Canada. BMC Fam Pract. 2021;22(1):201.

4. McDevitt AW, Snodgrass SJ, Cleland JA, Leibold MBR, Krause LA, Mintken PE. Treatment of individuals with chronic bicipital tendinopathy using dry needling, eccentric-concentric exercise and stretching; a case series. Physiother Theory Pract. 2020;36(3):397-407.
