# Supplementary material for: The long head of biceps at the shoulder: a scoping review
Source: BMC Musculoskelet Disord. 2023 Mar 28;24:232. doi: 10.1186/s12891-023-06346-5 (PMC10044783; doi:10.1186/s12891-023-06346-5)
Supplement: Supplementary file 19 — Supplementary Material 19 [file 12891_2023_6346_MOESM19_ESM.docx]

# Additional file 19: Supplementary Table 17_BMC.docx; Therapeutic injection

| Author | LOE | No | Participants/Intervention | Clinical outcomes | Results | Implications |
| --- | --- | --- | --- | --- | --- | --- |
| Aly et al. (2015) | II | 814 | 300 cadaveric Sh and  Five hundred fourteen patients: US-guided vs.  Landmark-guided CSI of the Sh inclusive of LHBT sheath. | Biceps sheath injection (US-guided vs landmark guided); Procedure accuracy; Pain relief; Function; Adverse events. | Accuracy (US-guided vs landmark guided) - 86.7% vs 26.7% (p<0.05). Pain - US group had a significantly greater reduction in pain (MD 1.9, 95% CI 1.2 to 2.6, p<0.00001). Function - US guided group had a greater improvement in function (MD 10.9, 95% CI 6.57 to 15.23, p<0.00001). Adverse events - No significant difference between groups. | US-guided CSI demonstrates greater accuracy, pain relief and improved function over landmark injections. |
| Gofeld et al. (2019) | V | 12 | Cadaver specimens: US-guided injected dye into the LHBT sheath at the level of the bicipital groove. | Surgical dissection and gross examination of the injected dye's intra- and extra-articular spread. | US-guided injectate into the LHBT sheath demonstrated the intraarticular spread of injectate into the GHJ and articular glenoid cartilage in 92% (11) of 12 specimens. | Continuity of the LHBT sheath and GHJ capsule.  It has limited diagnostic utility.  Potential for wanted or unwanted deposition of injectate into the intra-articular GHJ. |
| Hashiuchi et al. (2011) | II | 30 | Patients with LHB tenosynovitis and tendinitis: Group 1 -  US-guided contrast injection into LHBT sheath (n=15) vs. Group 2 - unguided contrast injection (n=15). | Accuracy of biceps sheath injection (US-guided vs unguided) under CTA. | A significant difference between ultrasound-guided and unguided injection groups for each location type (P < 0.05). US-guided accuracy; Type 1 = 86.7% (n=13) and Type 2 = 13.3% (n=2). Unguided accuracy; Type 1 = 26.7% (n=4), Type 2 = 40.0% (n=6) and Type 3 = 33.3% (n=5). | US-guided injections demonstrate greater accuracy than unguided, blind injections. |
| Mitra et al. (2011) | III | 6 | Patients with bicipital tendonitis: Fluoroscopically (contrast) guided  CSI into supraglenoid tubercle. | VAS; Positive Speed’s test; Complications. | Five of 6 patients (83%) experienced a reduction in pain (VAS) by 50% or more at follow-up. All patients (100%) had a negative Speed test at a follow-up visit. No complications immediately after the procedure or at follow-were observed. | Patents with bicipital tenonitis may respond to fluoroscopically guided CSI into the supraglenoid tubercle. |
| Petscavage-Thomas and Gustas (2016) | III | 90 | Patients with anterior Sh pain: Group 1 - Fluoroscopy guided CSI into LHBT sheath (n=50) vs. Group 2 - US guided CSI (n=53). | VAS, Complications, Location of injectate, First-pass success rate, Final-pass success rate of CSI. | No statistically significant difference in pain relief or complications between US guided vs fluoroscopy-guided CSI. The US guided showed pre-injection abnormalities of the biceps tendon in 47.5% of cases. The differences between groups were statistically significant for all grades of first-pass success (p<0.05). The first-pass success rate was 90.6% for the US-guided vs 74.0% fluoroscopy guided. 90.6% of US-guided injections are located within the LHBT sheath, 5.6% of US-guided injections inside the LHBT sheath 3.8% outside the LHBT sheath.  Final-pass success was not statistically significant between the groups (p=0.1815). The final pass success rate was 98.2% for the US-guided vs 92.0% for fluoroscopy:   - 74.0% of US-guided injections located within the LHBT sheath - 8% of US-guided injections inside the LHBT sheath - Type 3 - 18% outside the LHBT sheath | US-guided CSI has higher accuracy and initial and final pass success rates with similar pain relief and complication rates compared to fluoroscopy-guided CSI. |
| Yiannakopoulos et al. (2020) | II | 44 | Patients with LHB tendinosis: Group 1 - US guided CSI into biceps groove (n=22) vs. Group 2 - Unguided CSI (n=22) | Procedure Accuracy; Duration; Discomfort; VAS; SANE; Q-DASH. | US-guided vs unguided - Accuracy (100% vs 68%); Duration (64% vs 81%; p < 0.001); Discomfort (22% vs 35.5%; p<0.001). Clinical outcomes (Mean VAS, SANE and Q-DASH scores were similar between groups at four weeks (p < 0.05) and six months (p<0.05) after treatment. Overall mean VAS, SANE, and Q-DASH scores were superior in the US-guided group four weeks and six months (p < 0.05) after follow-up. | The US-guided injections are more accurate, produce less discomfort, are faster, produce improved clinical outcomes and are an effective treatment for LHB tendinosis. |
| Zhang et al. (2011) | I | 98 | Patients with isolated BB tendinitis: Group 1 - unguided CSI (n=45) vs. Group 2 -US guided CSI (n=53). | VAS; CS (Pain, ADLs, Strength, ROM). | VAS score unguided - 7.1 before injection vs. 4.2 at follow-up (p<0.01). VAS score guided - 6.9 before injection vs 2.1 at follow-up (p<0.01). The difference in VAS score (% change) between unguided and guided CSI was significant (p<0.05). The CSI unguided - 31.4 before injection vs. 73.5 at follow-up (p<0.01). The CS guided - 32.5 before injection vs. 85.5 at follow-up (p<0.01). The difference in CS (% change) between unguided and guided CSI was significant (p<0.05). | US-guided CSI is more accurate, produces improved clinical outcomes and is an effective treatment for LHB tendinitis. |

List of Abbreviations: Activities of Daily Living (ADL); Confidence Interval (CI); Constant Score (CS); Computed Tomographic Arthrography (CTA); Corticosteroid Injection (CSI); Glenohumeral Joint (GHJ); Long Head of Biceps (LHB); Long Head of Biceps Tendon (LHBT); P-value (p); Quick - Disabilities of the Arm, Shoulder and Hand (Q-DASH); Range of Motion (ROM); Shoulder (Sh); Single Assessment Numeric Evaluation (SANE); Ultrasound (US); Visual Analog Scale (VAS).

References

1. Aly AR, Rajasekaran S, Ashworth N. Ultrasound-guided shoulder girdle injections are more accurate and more effective than landmark-guided injections: a systematic review and meta-analysis. Br J Sports Med. 2015;49(16):1042-9.

2. Gofeld M, Hurdle MF, Agur A. Biceps Tendon Sheath Injection: An Anatomical Conundrum. Pain Med. 2019;20(1):138-42.

3. Hashiuchi T, Sakurai G, Morimoto M, Komei T, Takakura Y, Tanaka Y. Accuracy of the biceps tendon sheath injection: ultrasound-guided or unguided injection? A randomized controlled trial. J Shoulder Elbow Surg. 2011;20(7):1069-73.

4. Mitra R, Nguyen A, Stevens KJ. Fluoroscopically guided supraglenoid tubercle steroid injections for the management of biceps tendonitis. Pain Pract. 2011;11(4):392-6.

5. Petscavage-Thomas J, Gustas C. Comparison of Ultrasound-Guided to Fluoroscopy-Guided Biceps Tendon Sheath Therapeutic Injection. J Ultrasound Med. 2016;35(10):2217-21.

6. Yiannakopoulos CK, Megaloikonomos PD, Foufa K, Gliatis J. Ultrasound-guided versus palpation-guided corticosteroid injections for tendinosis of the long head of the biceps: A randomized comparative study. Skeletal Radiol. 2020;49(4):585-91.

7. Zhang J, Ebraheim N, Lause GE. Ultrasound-guided injection for the biceps brachii tendinitis: results and experience. Ultrasound Med Biol. 2011;37(5):729-33.
