# Supplementary material for: The long head of biceps at the shoulder: a scoping review
Source: BMC Musculoskelet Disord. 2023 Mar 28;24:232. doi: 10.1186/s12891-023-06346-5 (PMC10044783; doi:10.1186/s12891-023-06346-5)
Supplement: Supplementary file 20 — Supplementary Material 20 [file 12891_2023_6346_MOESM20_ESM.docx]

# Additional file 20: Supplementary Table 18_BMC.docx; Surgical management - LHB pathology

| Author | Study type | LOE | No | Pathology/Intervention/Follow up | Post-operative outcomes | Clinical outcomes | Implications |
| --- | --- | --- | --- | --- | --- | --- | --- |
| Abraham et al. (2016) | Systematic review | IV | 476 | LHBT lesions:   - ATD (n=205) - OTD (n=271)   Mean follow-up time:   - ATD (14-35 months) - OTD (15-90 months) | ASES, DASH, MEPS, ROM, SANE, SST, UCLA, VAS, WORC, WOSI | Among the 271 OTD patients, 98% had a good or excellent outcome, with a poor outcome in 5 patients (2%). Among the 205 patients who underwent ATD, 98% had a good or excellent outcome, with a poor outcome in 5 patients (2%). | PROMS:   - ATD = OTD |
| Aflatooni et al. (2020) | Clinical trial | III | 215 | LHBT pathology:   - ATD (n=111) - ATT (n=104)   Mean follow-up time:   - ATD = 30.7 ± 6.0 months (22–43) - ATT = 38.4 ± 5.8 months (22–57) | Patient satisfaction, Biceps cramping/spasms, Biceps pain, Sh pain, Biceps weakness, ADL limitations, Popeye deformity.  *Results dichotomised by concomitant procedures and patient demographics (age and gender). | Spasms/cramping (TT=20%; TD=8%; p=0.02; OR = 2.87, 95% CI 1.25–6.60). Biceps pain (TT=20%; TD=11%; p=0.09; OR = 1.96, 95% CI 0.91–4.25). Sh pain (TT=36%; TD=19%; p=0.01; OR = 2.37, 95% CI 1.27–4.41). Weakness (TT=17%; TD=11%; p=0.24; OR = 1.73, 95% CI 0.79–3.79). ADL limitations (TT=11%; TD=8%; p=0.64; OR = 1.34, 95% CI 0.53–3.38). Popeye sign (TT=14%; TD=11%; p=0.54; OR = 1.39, 95% CI 0.62–3.13). Similar high satisfaction scores ATD (96%) vs. ATT (91%) - (p=0.08). Increased rate of postoperative complications in ATT (59%) vs. ATD (37%) (p<0.01), which differed in the incidence of Sh pain, biceps spasms and cramping arm pain between procedures (p=0.04). No significant effect of gender on reported satisfaction or complications. The trend for older patients to report higher satisfaction and fewer downsides. Higher likelihood of patients undergoing ATT to experience a negative outcome vs. ATD. | SATISFACTION:   - ATD > ATT (high)   COMPLICATIONS:   - ATT > ATD (cramping arm pain) |
| Ahmed et al. (2021) | Systematic review and meta-analysis | II | 684 | LHBT pathology:   - TD (n=346) - TT (n=338)   Mean follow-up time:   - TT (3-23 months) - TD (3-25 months) | CS, ASES, VAS, Cramping arm pain, Bicipital groove TOP EFSI, FSSI, Popeye deformity, Reoperations, TD failure, RC retear, Adhesive capsulitis. | Both TT and TD showed equivalent levels of CS at 6 and 12 months, with a statistically significant (p<0.001) mean difference (D) but clinically irrelevant postoperative improvement of TD at 24 months. Important MD in ASES scores at 6,12, and 24 months follow-up. No significant difference was detected in postoperative pain (cramping, biceps groove tenderness) and strength (elbow Flex and forearm Sup) between TT and TD at 6, 12 and 24 months. There was a 2.46 times risk ratio for developing a Popeye’s deformity following TT vs. TD at follow-up (p<0.001). | PROMS:   - TD = TT (CS, ASES)   COMPLICATIONS:   - TT > TD (Popeye deformity) |
| Anil et al. (2020) | Network meta-analysis | III | 1804 | LHBT pathology:   - ASTD - (n=522) - AITD (n=160) - OTD (n=310) - ATT (n=812)   Mean follow-up time:   - All types (18-57 months) | ASES, ROM (Sh Flex + ER), Popeye deformity, Bicipital groove pain. | Significantly greater ASES (MD, 4.58; p=0.014) OTD vs. ATT. Significantly greater in CS for ASTD (MD, 2.46; p =0.031) vs. arthroscopic ATT. No significant difference in Sh ROM (ER + Flex) between TD techniques vs arthroscopic TT. Significantly lower incidence of Popeye deformity for ASTD (odds ratio [OR], 0.23; p<0.001) and OTD (OR, 0.25; p = 0.022) vs. ATT. The incidence of bicipital groove pain was increased after AITD (OR, 2.89; p=0.021) vs. ATT. | LHB-related Sh pathology:  PROMS:   - ATD OR OTD > ATT (CS, ASES)   SHOULDER ROM:   - ATD OR OTD = TT   COMPLICATIONS:   - ATT > ATD OR OTD (Popeye deformity) - AITD > ATT (bicipital groove pain) |
| Belay et al. (2019) | Clinical trial | II | 34 | LHB pathology:   - TD (n=14) + RC tear (n=5, 35%) - TT (n=20) + RC tear (n=14, 70%)   *56% with concomitant RC repair.  Follow-up interval:   - All types (3 months and two years) | VAS, ASES, SANE, Popeye deformity, Bicipital groove TOP, Narcotic use reduction. | VAS scores improved (p=0.02). Mean ASES scores improved (p = 0.001). Mean SANE scores improved (p=0.002). No statistically significant differences for BT vs TT were demonstrated for VAS, ASES, or SANE at two years follow-ups. No pain medication use was reported in 75% of TT patients at a 2-week postoperative visit vs. 33% for biceps TD (p=n.s). Popeye deformity in 25% of TT patients vs. 7% in TD patients at 3 months follow up (p=n.s). | PROMS (VAS, ASES, SANE):   - TT = TD (improved)   NARCOTIC USE:   - TT < TD (decreased use)   COMPLICATIONS:   - TT > TD (Popeye deformity) |
| Belk, Kraeutler, et al. (2021) | Systematic review and meta-analysis | I | 468 | LHBT or SLAP pathology:   - TD (n=236) - TT (n=232)   Mean follow-up time:   - All types (23.0 months) | ASES, VAS, CS, ROM, Strength testing (Sh, elbow, forearm), Popeye deformity. | No significant differences in ASES, VAS or CS between groups. No significant differences in Sh and elbow strength and ROM at the latest follow-up (n=1) study demonstrated significantly increased Sup strength in TD (p=0.02) vs. TT. Significantly more biceps were cramping in TD patients at 6-month follow-up compared with TT patients (p=0.043). Increased rate of Popeye deformity in patients undergoing biceps TT (23.3%) compared with TD (6.8%) (p<0.0001). | PROMS:   - TD = TT (ASES, VAS, CS)   COMPLICATIONS:   - TT > TD (Popeye deformity) - TD > TT (biceps cramping) |
| Belk, Thon, et al. (2021) | Systematic review | III | 707 | LHBT or SLAP pathology:   - ATD (n=326) - OTD (n=381)   Follow-up time:   - All types (9.7-50.2 months) | ASES, VAS, SANE, CS, Clinical failure, ROM, Bicipital groove pain, Strength. | No differences between ATD and OTD for PROMS and treatment failure rates. Significantly increased active Sh forward Flex ROM in OTD vs. ATD patients (p=0.049). Significantly more postoperative stiffness in ATD vs. OTD patients (p<0.05). | PROMS:   - ATD = OTD   TREATMENT FAILURE RATES:   - ATD = OTD   SHOULDER AROM:   - OTD > ATD (greater Sh Flex) - ATD > OTD (increased post-operative stiffness) |
| Michael J. Creech et al. (2016) | Systematic review | IV | 1,740 | LHBT pathology:   - TD surgical indications   Mean follow-up time:   - 43 months (12-372 months) | Surgical indications for LHB TD. | Commonly recorded surgical indications for LHB TD:   - partial tearing (51%) - instability (49%) - tenosynovitis (44%) - SLAP lesions (28%) - subjective Sh pain (28%) - positive clinical exam for LHB pain/pathology (26%) | Common indications for TD:   - LHB tearing, instability, and tenosynovitis. |
| Duchman et al. (2016) | Clinical trial | III | 45 | LHB pathology:   - ATD (n=20) - OTD (n=25)   Mean follow-up time:   - All types (3.2 ±   1.1 years) | SF-36 (PCS), SF-36 (MCS), ASES, DASH, SANE, SST, CS, would recommend surgery again, Bicipital groove TOP, Positive Speed’s test, Positive Yergason’s test, Biceps apex difference, Biceps circumference difference, Strength (Elbow Flex, forearm Sup), Strength (Sh Abd), ROM (Sh Flex, Abd, IR, ER), Wound Infection, Reoperation, Loss of proximal fixation. | Active Sh Flex was significantly decreased in the ATD group vs. OTD group (171.3 ± 11.7° vs.177.8 ± 9.3°; p=0.049). No other significant differences in PROMS, Physical examination findings, strength, ROM, and complications between ATD vs. OTD, respectively. SF-36 PCS (p=0.004), SF-36 MCS (p=0.379) and ASES (p<0.001) scores improved significantly from preoperative values. | PROMS:   - ATD = OTD (improved)   COMPLICATIONS:   - ATD = OTD   STRENGTH:   - ATD = OTD   SHOULDER AROM:   - OTD > ATD (greater Sh Flex) |
| Ge et al. (2015) | Systematic review and meta-analysis | I | 622 | LHBT lesions:   - TD (n=361) - TT (n=542)   Follow-up time:   - TD (24.5-62.4 months) - TT (22.2-62.4 months) | CS, VAS, ROM (Sh Flex, ER, IR), Popeye deformity, Arm cramping pain, Patient satisfaction, Surgical time. | Significantly greater CS (p=0.02) in TD vs. TT. Significantly lower incidence of Popeye deformity (p<0.001) in TD vs. TT. Significantly lower incidence of cramp arm pain (p=0.04) in TD vs. TT. Significantly longer surgical times (p<0.001) in TD vs. TT. No significant difference between TT VS. TD in patient satisfaction (p=0.94). | SATISFACTION   - TD = TT   PROMS:   - TD > TT (CS)   COMPLICATIONS   - TT > TD (Popeye deformity) - TT > TD (Cramping arm pain)   SURGICAL TIMES   - TD > TT (increased) |
| Horan et al. (2020) | Clinical trial | III | 41 | Isolated SLAP II lesions (overhead throwing athletes):   - SLAP repair (n=27) - TD (n=14)   Minimum 2-year follow-up | ASES, SANE,  Q-DASH, SF-12 PCS. Return to sports and patient satisfaction, clinical failures requiring revision surgery and complications. | No significant differences in PROMS (p>0.05):   - ASES score - mean 91.6 ± 11.3 vs 88.6 ± 16.9 - SANE score - mean 77.5 ± 28.0 vs. 82.3 ± 24.4 - Q-DASH score - mean 9.2± 12.2 vs 9.4 ± 14.5, - SF-12PCS mean 52.0 ± 6.1 vs 52.6 ± 7.8.   No significant differences in return to sports rate TD vs. SLAP repair (91% vs 91%, p>0.05). | PROMS:   - TD = SLAP repair (ASES, SANE, Q-DASH, SF-12PCS)   RETURN TO SPORTS RATE   - TD = SLAP repair (high) |
| Kooistra et al. (2021) | Systematic review and meta-analysis | III | 2,191 | LHB tendinopathy:   - TD (n=1003) - TT (n=1188)   Follow-up time:   - All types (1-10 years) | CS, ASES, EFSI, FSSI, Popeye deformity, Cramping arm pain, VAS. | No clinically significant differences PROMS:   - CS (mean difference, 0.9 points). - ASES Score (mean difference, 1.1 points). - Sh pain VAS (mean difference, -0.3 points). - Elbow Flex strength loss (mean difference, 0% - Forearm Sup strength (mean difference, 3%). - A Popeye deformity (odds ratio, 0.32) was more commonly seen in patients treated with TT (23%). vs TD (9%). Increased incidence of cramping arm pain following TT. | PROMS:   - TD = TT (CS, ASES, VAS, EFSI, FSSI)   COMPLICATIONS:   - TT > TD (Popeye deformity) - TT>TD (cramping arm pain) |
| Lim et al. (2011) | Clinical study | III | 132 | LHBT injuries:   - ATT (n=132)   Mean follow-up time   - 21 months (12-53) | Popeye deformity, Cramping arm pain, Elbow Flex strength (dynamometer).  Results dichotomised by  Patient demographics and BMI. | Popeye deformity (45%):   - Significantly more frequent in men (76%) vs. women (31%) (p<0.001). - Male sex is significantly associated with the development of Popeye deformity, with an odds ratio of 10.21 vs. female (95% CI, 3.97 to 26.27; p<0.001). - Cramp-like arm pain (8%). - Decreased elbow Flex strength (45%). | COMPLICATIONS:   - Popeye deformity (45%) - Cramp-like arm pain (8%) - decreased elbow Flex strength (45%)   RISK FACTORS   - Male > Female (Popeye deformity) |
| Liu et al. (2021) | Systematic review and meta-analysis | IV | 448 | LHBT Lesions:   - TD (n=227) - TT (n=221)   Follow-up time:   - TD (12-58 months) - TT (12-57 months) | Popeye deformity, Cramping arm pain, CS, VAS, ASES, EFSI, FSSI, SST, UCLA, ROM (Sh Flex + ER), RC retear rate. | Significantly higher incidence of the Popeye sign and cramping pain after TT vs TD. Significantly worse CS, SST scores and forearm Sup strength after TT vs TD. Equivocal VAS scores after TD vs TT. No significant difference in ASES and UCLA scores, EFSI and Sh Flex or ER ROM for TD vs TT. No significant difference in the incidence of RC retears. | PROMS:   - TD > TT (CS, SST FSSI) - TD = TT (ASES, UCLA, EFSI, ROM)   COMPLICATIONS:   - TT > TD (Popeye deformity) - TT > TD (cramping arm pain) |
| MacDonald et al. (2020) | Randomised control trial | I | 114 | LHBT lesions:   - TD (n=57) - TT (n=57)   Follow-up time:   - two2 years | ASES, WORC, Strength (Elbow and supination strength), Operative time, Complications  incidence of revision surgery, MRI (the integrity of TD). | Post-operative ASES (32.3%) and WORC (37.3%) scores improved significantly for both TD and TT groups (p<0.001), with no difference between groups. Relative risk of Popeye deformity, in TT (33%) vs TD group (10%) at 24 months was 3.5 (95% CI, 1.26-9.70; p=0.016). Pain VAS improved from 3 to 24 months postoperatively (p<0.001) with no difference between groups. No difference in cramping pain between groups or improvement over time. No differences between groups in elbow Flex strength or Sup strength. Follow-up MRI at postoperative 12 months showed TD was intact for all patients. | PROMS:   - TD = TT (ASES, WORC, VAS)   STRENGTH   - TD = TT (elbow and forearm strength)   COMPLICATIONS:   - TT > TD (Popeye deformity) - TD = TD (cramping arm pain) |
| Mariani et al. (1988) | Clinical trial | III | 56 | LHBT rupture:   - TD (n=26) - Non-surgical MX (n=30)   *Non-surgical care not defined  Mean follow-up time:   - TD = 13 years (3-22) - Non-surgical = 4.6 years (2-15) | Residual arm pain, reported cosmetic deformity, Subjective elbow weakness, the Patient response to change,  Biomechanical Ax:  Elbow/forearm/grip strength, Elbow, and Sh ROM. | No significant difference in postoperative arm pain (p<0.0001) – low in both groups. A significant difference in the subjective elbow and forearm strength in non-surgical vs TD (p<0.0001). No significant difference in elbow/Sh ROM (p<0.001). There was a significant difference in reported cosmetic deformity in the non-surgical vs TD group (p<0.0001). Patients treated non-surgically returned to work more quickly but often at a lesser capacity (p<0.01). Statistically significant loss of isometric elbow Flex strength (8%; p<0.025) and forearm Sup strength (21%; p<0.005) in non-surgical group vs. TD group (p≤0.05). | COMPLICATIONS:   - Non-surgical = TD (Self-reported cosmetic deformity) - Non-surgical = TD (residual arm pain).   TIME TO RETURN TO WORK:   - Non-surgical < TD but at lesser capacity long-term.   STRENGTH:   - TD > non-surgical (isometric elbow Flex and forearm Sup). - TD > non-surgical (subjective elbow and forearm strength). |
| Mirzayan et al. (2020) | Clinical study | III | 192 | Anterior Sh pain with LHB-related Sh pathology:   - Sh pathology (mixed) - ATT (n=192) + concomitant Sh surgery   Mean ± SD follow-up time:   - All types (10.6 ± 13.6 months) | Cosmetic deformity, Subjective weakness, Continued anterior Sh pain and cramping.  *Results dichotomised by patient’s demographics BMI, smoking status, workers’ compensation status, and intraoperative LHBT morphology. | Overall complication rate (37%), Popeye deformity (14.1%), Subjective weakness (10.4%), Cramping (10.4%), Recalcitrant anterior Sh pain over biceps groove (7.8%).  Decreasing odds of postoperative anterior Sh pain, fewer complications observed in females 3.9 times the odds of a cosmetic Popeye deformity in males. Active worker’s compensation claims have 12.5 times the odds of continued postoperative anterior Sh pain. | Anterior Sh pain with LHB-related Sh pathology.    COMPLICATIONS:   - Popeye deformity - subjective weakness - cramping arm pain - persistent anterior Sh pain   Male gender, younger age, and active workers’ compensation are associated with increased complications following ATT. |
| Pozzetti Daou et al. (2021) | Systematic review and meta-analysis | II | 615 | LHBT lesions:   - TD (n= 309) - TT (n=306)   Median follow-up time:   - All types (2 years) | VAS, CS, Complication, Popeye sign | No significant long-term differences between TT and TD for VAS scores (p=0.36) and CS (p=0.06). No significant difference in major complications between TT and TD (p=0.70). Popeye sign are significantly more frequent in the TT vs. TD (p<0.001). | PROMS:   - TD = TT (VAS pain) - TD = TT (CS)   COMPLICATIONS:   - TT > TD (Popeye deformity) |
| Derek F. P. van Deurzen et al. (2020) | Systematic review and meta-analysis | III | 409 | LHB tendinopathy:   - Suprapectoral TD (n=187) - Subpectoral TD (n=222)   Follow-up time:   - All types (1-2 years) | ASES, VAS, CS, Bicipital groove pain, Popeye deformity. | A significant difference in ASES scores (p=0.01) but clinically irrelevant. No statistically significant differences between subpectoral and suprapectoral TD groups in post-operative scores for anterior Sh pain VAS (p=0.90), CS (p=0.90), bicipital groove pain (p=0.40) and the prevalence of a Popeye deformity (p=0.10). | PROMS:   - Suprapectoral TD = Subpectoral TD (VAS, CS)   COMPLICATIONS:   - Suprapectoral TD = Subpectoral TD (Bicipital groove pain) - Suprapectoral TD = Subpectoral TD (Popeye deformity) |
| Woodmass et al. (2021) | Secondary analysis of Randomised control trial | II | 114 | LBH tendon lesions:   - TD (n=57) - TT (n=57)   Follow-up time:   - All types (24 months) | Popeye deformity, Patient-reported evaluation of appearance, Cosmetic deformity, Cramping arm pain, ASES, WORC, EFSI, MRI (LHBT integrity and retraction).  *Results dichotomised by gender and BMI. | The overall incidence of Popeye deformity at two years was 21%. The odds of a Popeye deformity in the TT group (33%) were 4.3 times greater than in the TD group (9.5%) and were Statistically significant (p=0.018). Significant correlation with increasing satisfaction and increasing age (r=0.640, p=0.025). No association with gender (r = -0.155, p= 0.527) or BMI (r =-0.221, p= 0.057). Male gender trending toward significance (OR 7.33, 95% CI 0.87-61.91, p=0.067) as a predictor of perceived deformity. No significant differences between those with and without a clinically observed Popeye deformity for pain, cramping, ASES, or WORC. | COMPLICATIONS:   - TT > TD (Popeye deformity) - Younger patients were less satisfied with a Popeye deformity. |
| Zhou et al. (2021) | Systematic review and meta-analysis | I | 673 | LHBT lesions:   - TD (n=340) - TT (n=333)   Follow-up time:   - TD (12-25 months) - TT (12-25 months) | CM, ASES, VAS, EFSI, FSSI, Popeye deformity, Cramping arm pain, Operative time.  *Trial sequential analysis (TSA) was conducted to reduce the risk of random  Errors. | There was a significant difference between TT and TD in CS in favour of the TD group (p=0.04), whilst TSA revealed inconclusive results. A significant difference in the incidence of Popeye deformity between TT (25%) and TD (7.8%), with a risk ratio of 0.33 in favour of TD (p<00001). A significant difference between TT and TD and reduced operative time in favour of TT (p<00001). No significant differences between TT and TD in ASES score (p=0.71), VAS (p=0.79), EFSI (p=0.85), FSSI (p=0.23), and arm cramping pain (p=0.61). | PROMS:   - TD = TT (CS) – inconclusive? - TD = TT (ASES, VAS, EFSI, FSSI)   COMPLICATIONS:   - TT > TD (Popeye deformity) - TT = TD (cramping arm pain)   OPERATIVE TIME:   - TD < TT |
| Zhu et al. (2021) | Meta-analysis | I | 454 | LHBT pathology:   - TD (n=227) - TT (n=227)   Mean follow-up time:   - TD (12-25 months) - TT (12-25.1 months) | CS, ASES, VAS, EFSI, FSSI, Popeye deformity  *Results dichotomised by demographics (age and gender). | No statistically significant difference between TT and TD for PROMS including CS (p=0.23), ASES (p=0.56), pain VAS (p=0.96) or EFSI (p=0.65), regardless of surgical treatment. A statistically significant difference in the incidence and risk of Popeye deformity in favour of TD (7.5%; RR 3.07; p<0.001) compared with TT (24.7%). | PROMS:   - TD = TT (CS, ASES, VAS, EFSI)   COMPLICATIONS:   - TT > TD (Popeye deformity) |

List of Abbreviations: Abduction (Abd); Activities of Daily Living (ADL); Arthroscopic Intracuff Tenodesis (AITD); American Shoulder and Elbow Surgeons (ASES); Arthroscopic Suprapectoral Tenodesis (ASTD); Arthroscopic Tenodesis (ATD); Arthroscopic Tenotomy (ATT); Body Mass Index (BMI); Constant Score (CS); Disabilities of the Arm, Shoulder and Hand (DASH); Elbow Flexion Strength Index (EFSI); External Rotation (ER); Forearm Supination Strength Index (FSSI); Flexion (Flex); Internal Rotation (IR); Level of Evidence (LOE); Long Head of Biceps (LHB); Long Head of Biceps Tendon (LHBT); Mean Difference (MD); Mayo Elbow Performance Score; (MEPS); Not Stated (ns); Open Tenodesis (OTD); P-value (p); Patient Reported Outcome Measure (PROMS); Quick - Disabilities of the Arm, Shoulder and Hand (Q-DASH); Pearson's Correlation Coefficient (r); Range of Motion (ROM); Relative Risk Ratios (RR); Rotator Cuff (RC); Short Form 36 Item Health Survey - Physical Component Summary (SF-36 PCS); Short Form 36 Item Health Survey – Mental Component Summary (SF-36 MCS); Short Form 12 Item Health Survey - Physical Component Summary (SF-12 PCS); Shoulder (Sh); Single Assessment Numeric Evaluation (SANE); Standard Deviation (SD); Supination (Sup); Simple Shoulder Test (SST); Superior Labrum Anterior to Posterior (SLAP); Tenderness on Palpation (TOP); Tenodesis (TD); Tenotomy (TT); University of California at Los Angeles (UCLA); Visual Analog Scale (VAS); Western Ontario Rotator Cuff Index (WORC); Western Ontario Shoulder Instability Index (WOSI).

References

1. Abraham VT, Tan BH, Kumar VP. Systematic Review of Biceps Tenodesis: Arthroscopic Versus Open. Arthroscopy. 2016;32(2):365-71.

2. Aflatooni JO, Meeks BD, Froehle AW, Bonner KF. Biceps tenotomy versus tenodesis: patient-reported outcomes and satisfaction. J Orthop Surg Res. 2020;15(1):56.

3. Ahmed AF, Toubasi A, Mahmoud S, Ahmed GO, Al Ateeq Al Dosari M, Zikria BA. Long head of biceps tenotomy versus tenodesis: a systematic review and meta-analysis of randomized controlled trials. Shoulder Elbow. 2021;13(6):583-91.

4. Anil U, Hurley ET, Kingery MT, Pauzenberger L, Mullett H, Strauss EJ. Surgical treatment for long head of the biceps tendinopathy: a network meta-analysis. J Shoulder Elbow Surg. 2020;29(6):1289-95.

5. Belay ES, Wittstein JR, Garrigues GE, Lassiter TE, Scribani M, Goldner RD, et al. Biceps tenotomy has earlier pain relief compared to biceps tenodesis: a randomized prospective study. Knee Surg Sports Traumatol Arthrosc. 2019;27(12):4032-7.

6. Belk JW, Kraeutler MJ, Houck DA, Chrisman AN, Scillia AJ, McCarty EC. Biceps tenodesis versus tenotomy: a systematic review and meta-analysis of level I randomized controlled trials. J Shoulder Elbow Surg. 2021;30(5):951-60.

7. Belk JW, Thon SG, Hart J, McCarty EC, Jr., McCarty EC. Subpectoral versus suprapectoral biceps tenodesis yields similar clinical outcomes: a systematic review. J ISAKOS. 2021;6(6):356-62.

8. Creech MJ, Yeung M, Denkers M, Simunovic N, Athwal GS, Ayeni OR. Surgical indications for long head biceps tenodesis: a systematic review. 2016. p. 2156-66.

9. Duchman KR, DeMik DE, Uribe B, Wolf BR, Bollier M. Open Versus Arthroscopic Biceps Tenodesis: A Comparison of Functional Outcomes. Iowa Orthop J. 2016;36:79-87.

10. Ge H, Zhang Q, Sun Y, Li J, Sun L, Cheng B. Tenotomy or tenodesis for the long head of biceps lesions in shoulders: a systematic review and meta-analysis. PLoS One. 2015;10(3):e0121286.

11. Horan M, Dekker T, Goldenberg B, Dornan G, Peebles L, Provencher M, et al. Superior Labrum Anterior-Posterior (SLAP) Repair Versus Subpectoral Biceps Tenodesis for Isolated SLAP II Lesions in Overhead Athletes Aged Younger 35 Years: A Comparison of Minimum Two-Year Outcomes. Orthopaedic Journal of Sports Medicine. 2020;8(7_suppl6).

12. Kooistra B, Gurnani N, Weening A, van Deurzen D, van den Bekerom M. Tenotomy or Tenodesis for Tendinopathy of the Long Head of the Biceps Brachii: An Updated Systematic Review and Meta-analysis. Arthrosc Sports Med Rehabil. 2021;3(4):e1199-e209.

13. Lim TK, Moon ES, Koh KH, Yoo JC. Patient-related factors and complications after arthroscopic tenotomy of the long head of the biceps tendon. Am J Sports Med. 2011;39(4):783-9.

14. Liu H, Song X, Liu P, Yu H, Zhang Q, Guo W. Clinical Outcomes of Arthroscopic Tenodesis Versus Tenotomy for Long Head of the Biceps Tendon Lesions: A Systematic Review and Meta-analysis of Randomized Clinical Trials and Cohort Studies. Orthop J Sports Med. 2021;9(4):2325967121993805.

15. MacDonald P, Verhulst F, McRae S, Old J, Stranges G, Dubberley J, et al. Biceps Tenodesis Versus Tenotomy in the Treatment of Lesions of the Long Head of the Biceps Tendon in Patients Undergoing Arthroscopic Shoulder Surgery: A Prospective Double-Blinded Randomized Controlled Trial. Am J Sports Med. 2020;48(6):1439-49.

16. Mariani EM, Cofield RH, Askew LJ, Li GP, Chao EY. Rupture of the tendon of the long head of the biceps brachii. Surgical versus nonsurgical treatment. Clin Orthop Relat Res. 1988;228(228):233-9.

17. Mirzayan R, McCrum C, Butler RK, Alluri RK. Risk Factors and Complications Following Arthroscopic Tenotomy of the Long Head of the Biceps Tendon. Orthop J Sports Med. 2020;8(2):2325967120904361.

18. Pozzetti Daou J, Nagaya DY, Matsunaga FT, Sugawara Tamaoki MJ. Does Biceps Tenotomy or Tenodesis Have Better Results After Surgery? A Systematic Review and Meta-analysis. Clin Orthop Relat Res. 2021;479(7):1561-73.

19. van Deurzen DFP, Gurnani N, Alta TDW, Willems JH, Onstenk R, van den Bekerom MPJ. Suprapectoral versus subpectoral tenodesis for Long Head Biceps Brachii tendinopathy: A systematic review and meta-analysis. Orthopaedics & traumatology, surgery & research. 2020;106(4):693-700.

20. Woodmass JM, McRae SMB, Lapner PL, Sasyniuk T, Old J, Stranges G, et al. Effect of age, gender, and body mass index on incidence and satisfaction of a Popeye deformity following biceps tenotomy or tenodesis: secondary analysis of a randomized clinical trial. J Shoulder Elbow Surg. 2021;30(8):1733-40.

21. Zhou P, Liu J, Deng X, Li Z. Biceps tenotomy versus tenodesis for lesions of the long head of the biceps tendon: A systematic review and meta-analysis of randomized controlled trials. Medicine (Baltimore). 2021;100(3):e23993.

22. Zhu XM, Leroux T, Ben-David E, Dennis B, Gohal C, Kirsch JM, et al. A meta-analysis of level I evidence comparing tenotomy vs tenodesis in the management of long head of biceps pathology. J Shoulder Elbow Surg. 2021;30(5):961-8.
