# Supplementary material for: The long head of biceps at the shoulder: a scoping review
Source: BMC Musculoskelet Disord. 2023 Mar 28;24:232. doi: 10.1186/s12891-023-06346-5 (PMC10044783; doi:10.1186/s12891-023-06346-5)
Supplement: Supplementary file 21 — Supplementary Material 21 [file 12891_2023_6346_MOESM21_ESM.docx]

# Additional file 21: Supplementary Table 19_BMC.docx; Surgical management - Healthy LHB and rotator cuff pathology

| Author | Study type | LOE | No | Pathology/Intervention | Post-operative outcomes | Clinical outcomes | Implications |
| --- | --- | --- | --- | --- | --- | --- | --- |
| Boileau et al. (2007) | Clinical trial | III | 68 (72) | Irreparable RC tear:   - adjunctive ATD (n=33) - adjunctive ATT (n=39)   Mean follow-up time:   - ATD = 34 months (24-50) - ATT = 36 months (24-76) | CS, AROM & PROM (Sh Flex, ER, IR), Popeye sign, Cramping arm pain, Pain in the bicipital groove, Patient satisfaction, Duration of follow-up, Imaging evaluation (X-RAY/CTA), Acromiohumeral distance, Acetabularization, GHJ OA. | Fifty-three patients (78%) patients satisfied with postoperative result (very satisfied 46%, satisfied 32%, disappointed 15%, and 7% dissatisfied)  Significant improvements in overall CS (p<0.001) for pain, activity, and mobility but not for strength. Significant improvements in overall active Sh forward Flex ROM (p<0.001). No significant difference in reduced acromiohumeral distance (mean 1.1 ± 1.9 mm) between pre and postoperative measures. No significant difference in CS or cramping arm pain. Significant higher incidence of “Popeye” sign in patients treated with ATT (62%) vs. ATD (3%) (p<0.001). | SATISFACTION (satisfied)   - ATD = ATT   PROMS:   - ATD = ATT (CS)   ROM:   - ATD = ATT (Sh Flex)   COMPLICATIONS:   - ATT > ATD (Popeye deformity) |
| DeFroda et al. (2021) | Cost analysis | IV | 3635 | Repairable RC tear (cost comparison):   - RCR + adjunctive ATD (n=2847) - RCR + adjunctive OTD (n=788)   *Follow-up time not applicable | Surgical costs, Operative time, Hospital admission, Facility location, Surgical caseload, Demographic, Comorbidity level, and Concomitant surgery. | Significant higher mean surgical costs of RCR with ATD ($31,461) vs. RCR with OTD ($21,013; p<0.001). Significantly lower surgical costs of RCR with OTD $5542 vs. ATD (p<0.001). Significantly lower time costs of RCR with 9 minutes for ATD vs. OTD (p=0.002).  *Note - Secondary outcomes dichotomised by patient demographics are not included in this analysis. | Cost comparison:   - RCR + ATD > RCR + OTD (higher surgical costs - RCR + ATD < RCR + OTD (lower time costs) |
| Hughes et al. (2021) | Clinical trial | III | 100 | Repairable SSPs:   - RCR (n=57) - RCR + adjunctive ATD (n=18) - RCR + adjunctive OTD (n=9) - RCR + adjunctive TT (n=16)   Follow-up time:   - All types (Min 12 months) | Operative time, Cost analysis, Complication, VAS pain, SSV, ROM (Sh), Strength (Sh). | Significantly longer surgical times in RCR + adjunctive OTD vs. RCR in isolation (p<0.05). Significantly fewer operative costs for RCR + no LHBT surgery vs. RCR + adjunctive OTD (p<0.05) and RCR + adjunctive TT (p<0.05). Significant fewer operative costs for RCR + adjunctive TT vs. SSP repair + adjunctive OTD (p<0.05). No significant differences between surgical groups for complications (p=0.90) and all PROMS, VAS pain, ROM, and strength parameters (p > 0.05). Significant variation in surgical times and operative costs between groups (p<0.05):   - SSP repair and open LHB TD (114 minutes/$7427) - SSP repair and arthroscopic LHB TD (94 minutes/$6274) - SSP repair and LHB TT (n=16) - (84 minutes/$4684) - SSP repair with no LHBT surgery (73 minutes/$4397) | SURGICAL TIMES:   - RCR + OTD > RCR   SURGICAL COSTS:   - RCR + OTD > RCR - RCR + TT > RCR - RCR + OTD > RCR + TT   PROMS & COMPLICATIONS:   - Nil significant differences |
| Leroux et al. (2015) | Systematic review and meta-analysis | IV | 565 | Repairable SSPs:   - RCR + adjunctive TD (n=302) - RCR + adjunctive TT (n=263)   Mean follow-up time:   - All types (12-62.4 months) | UCLA, ASES, CS, SST, VAS, Popeye deformity, Cramping arm pain, Patient satisfaction. | A statistically significant difference (p<0.01) in postoperative CS after TD (92.8) vs TT (90.6) at a mean follow-up of 25.5 months. However, the difference was less than the reported minimal clinically important difference of 10.4 points. The rate of Popeye deformity was significantly (p<0.01) less after TD (3.9%) vs TT (15.5%). No significant difference in the rate of biceps cramping or patient satisfaction. | SATISFACTION:   - TD=TT   PROMS:   - TD > TT (CS)   COMPLICATIONS:   - TT > TD (Popeye deformity) - TT = TD (Cramping arm pain) |
| Meraner et al. (2016) | Clinical trial | III | 53 | Repairable RC tear:   - RCR + adjunctive TD (n=24) - RCR + adjunctive TT (n=29)   Mean follow-up time:   - All types 34 months (27–38) | CS, VAS, AROM (Sh Flex, Abd), Cramping arm pain, Popeye deformity, Sh force (isometric Flex, Abd). | No significant difference in post-surgical outcomes between TD vs TT for CS, VAS, and ROM:   - CS (86.6% vs. 81.3%; p = 0.120) - VAS (0 vs. 0; p = 0.421) - ROM - Flex (180º vs.180º; p = 0.833) - ROM – Abd (180º vs.180º; p = 0.472)   Low incidence of cramps TD vs TT (0% vs. 0%) and Popeye deformity TD vs TT (0% vs 1.9 %; p = 1.000). Significant differences between TT vs TD in post-surgical improvements in mean (5.6 vs. 6.5; p = 0.004) and maximum (6.5 vs. 8.0; p = 0.002) Sh Abd force, but not in mean (12.5 vs.13.0, p = 0.509) and maximum (14.5 vs.14.5, p = 0.921) Flex force. | PROMS:   - RCR + TD = RCR + TT (CS + VAS)   AROM:   - RCR + TD = RCR + TT (Flex + Abd)   COMPLICATIONS:   - TT > TD (Popeye deformity) - TT > TD (cramping arm pain)   STRENGTH:   - TD > TT (Sh Abd) - TD = TT (Sh Flex) |
| Walch et al. (2005) | Case study | III | 291 | Irreparable RC tears:   - ATT (n=307)   Mean follow-up time:   - 57 months (24-168) | CS, AROM (Sh Flex, ER, IR), Drop arm sign, Hornblower’s sign, Patient satisfaction. Imaging evaluation (XRAY, CTA, MRI): Acromiohumeral distance, Glenohumeral OA, Fatty infiltration of ISP and SSC. | Significant increase in mean CS from 48.4 points preoperatively to 67.6 points postoperatively (p<0.0001). 87% of patients were satisfied or very satisfied with the result. Acromiohumeral interval decreased by a mean of 1.3 mm postoperatively and was associated with a longer follow-up duration (p<0.0001). | SATISFACTION:   - ATT (high)   PROMS:   - ATT (CS)   ACROMIOCLAVICULAR DISTANCE   - ATT (decreased and associated with longer follow-up) |

List of Abbreviations: Abduction (Abd); Active Range of Motion (AROM); American Shoulder and Elbow Surgeons (ASES); Arthroscopic Tenodesis (ATD); Arthroscopic Tenotomy (ATT); Constant Score (CS); Computed Tomographic Arthrography (CTA); External Rotation (ER); Flexion (Flex); Glenohumeral Joint (GHJ); Infraspinatus (ISP); Internal Rotation (IR); Level of Evidence (LOE); Magnetic Resonance Imaging (MRI); Open Tenodesis (OTD); Osteoarthritis (OA); Passive Range of Motion (PROM); P-value (p); Patient Reported Outcome Measure (PROMS); Range of Motion (ROM); Rotator Cuff (RC); Rotator Cuff Repair (RCR); (SSC) Subscapularis; (SSP) Supraspinatus; Simple Shoulder Test (SST); Tenodesis (TD); Tenotomy (TT); University of California at Los Angeles (UCLA); Visual Analog Scale (VAS).

References

1. Boileau P, Baque F, Valerio L, Ahrens P, Chuinard C, Trojani C. Isolated arthroscopic biceps tenotomy or tenodesis improves symptoms in patients with massive irreparable rotator cuff tears. J Bone Joint Surg Am. 2007;89(4):747-57.

2. DeFroda SF, Li L, Milner J, Bokshan SL, Owens BD. Cost comparison of arthroscopic rotator cuff repair with arthroscopic vs. open biceps tenodesis. J Shoulder Elbow Surg. 2021;30(2):340-5.

3. Hughes JD, Gibbs CM, Drummond M, Vaswani R, Ayinon C, Fongod E, et al. Failure rates and clinical outcomes after treatment for long-head biceps brachii tendon pathology: a comparison of three treatment types. JSES Int. 2021;5(4):630-5.

4. Leroux T, Chahal J, Wasserstein D, Verma NN, Romeo AA. A Systematic Review and Meta-analysis Comparing Clinical Outcomes After Concurrent Rotator Cuff Repair and Long Head Biceps Tenodesis or Tenotomy. Sports Health. 2015;7(4):303-7.

5. Meraner D, Sternberg C, Vega J, Hahne J, Kleine M, Leuzinger J. Arthroscopic tenodesis versus tenotomy of the long head of biceps tendon in simultaneous rotator cuff repair. Arch Orthop Trauma Surg. 2016;136(1):101-6.

6. Walch G, Edwards TB, Boulahia A, Nove-Josserand L, Neyton L, Szabo I. Arthroscopic tenotomy of the long head of the biceps in the treatment of rotator cuff tears: Clinical and radiographic results of 307 cases. 2005. p. 238-46.
