# Supplementary material for: The long head of biceps at the shoulder: a scoping review
Source: BMC Musculoskelet Disord. 2023 Mar 28;24:232. doi: 10.1186/s12891-023-06346-5 (PMC10044783; doi:10.1186/s12891-023-06346-5)
Supplement: Supplementary file 22 — Supplementary Material 22 [file 12891_2023_6346_MOESM22_ESM.docx]

# Additional file 22: Supplementary Table 20_BMC.docx; Surgical management – Rotator cuff and LHB pathology

| Author | Study type | LOE | No | Pathology/Intervention | Post-operative outcomes | Clinical outcomes | Implications |
| --- | --- | --- | --- | --- | --- | --- | --- |
| Godenèche et al. (2018) | Clinical trial | III | 249 | Repairable RC tear (n=249):   - ARCR n=153 - ORCR n=96   Isolated SSP and no LHB pathology:   - RCR (n=106) - RCR + adjunctive TD (n=9) - RCR + adjunctive TT (n=5)   Isolated SSP and LHB pathology:   - RCR (n=9) - RCR + adjunctive TD (n=70) - RCR + adjunctive TT (n=50)   *Minimum follow-up time:   - All types = ten years | CS, SSV, SST, MRI evaluation of tendon healing (n=182). | RCR +/- adjunctive LHB surgery:   - No significant differences in CS for patients who had RCR and no LHB pathology without adjunctive TD or TT (77.1 ± 11.7) vs. patients who had RCR with LHB pathology with adjunctive TD (79.8 ± 11.5, p = 0.104) or TT (75.3 ± 10.7, p = 0.420). - CS was significantly better for patients undergoing RC with LHB pathology and TD vs. TT (p=0.025).   MRI evaluation (n=182):   - SSP was retorn in 35 Sh (19%). Retear rates were similar for patients who had no adjuvant procedures (21.9%) and those who had adjunctive TD (20.4%, p = 0.472) or TT (16.7%, p = 0.535). | - Long-term study - RCR = isolated SSP repair   PROMS (CS):   - RC tear + normal biceps (RCR = RCR + TD or TD) - RCR + pathological LHB (RCR + TD or TD > RC tear)   *Adjunctive LHB surgery should be avoided. With a normal LHB intraoperatively.  TD provides better clinical outcomes in function and strength than TT in a patient undergoing RC repair with intraoperative LHB biceps pathology. |
| Gurnani et al. (2016) | Systematic review and meta-analysis | IV | 650 | Mixed Sh pathology:   - TD (n=328) - TT (n=322)   Mean follow-up time:   - All types = 24 months | CS, EFSI, FSSI, Popeye deformity, cramping arm pain. | Clinical outcomes:   - No significant difference in CS (p=0.07) between TD (n=293) vs. TT (n=286). - No significant difference in EFSI (p=0.95) between TD (n=190) vs. TT (n=181). - No significant difference in FSSI (p=0.82) between TD (n=109) vs. TT (n=113) - Decreased incidence of Popeye deformity (p<0.0001 in favour of TD (n=309) vs. TT (n=305). - Decreased incidence of cramping arm pain (p=0.01) in favour of TD (n=222) vs. TT (n=224). | PROMS:   - TD = TT (CS, EFSI, FSSI)   COMPLICATIONS:   - TT > TD (Popeye deformity) - TT > TT (cramping arm pain) |
| Lim et al. (2020) | Clinical trial | III | 35 | Concomitant RC tear and SLAP lesions:   - RCR + SLAP repair (n=17) - RCR + adjunctive TD (n=18)   Mean ± SD follow-up time:   - All types = 29.4 ± 11.4 months (24–84) | ROM (Sh Flex, ER, IR), VAS, ASES, CS, UCLA, MRI (RC integrity cuff and SLAP complex). | No significant differences in clinical outcomes between ARCR and SLAP repair and ARCR and adjunctive TD. Significant improvement in the pain (VAS) and all functional (ASES, CS, UCLA) scores for both RCR with SLAP repair and RCR with adjunctive TD (p<0.001). Significant improvement in Sh AROM postoperatively for both RCR + SLAP repair and RCR + adjunctive TD (p<0.05):   - Flex (°) - SLAP repair (p=0.048) vs adjunctive TD (p=0.049) - ER (90°) - SLAP repair (p=0.001) vs adjunctive TD (p=0.002) - ER (0°) - SLAP repair (p=0.001) vs adjunctive TD (p=0.011) - IR (0°) - SLAP repair (p=0.003) vs adjunctive TD (p=0.028)   MRI evaluation:   - Similar retear rate of repaired RC on post-operative MRI in SLAP repair group (11.8%) vs adjunctive LHB TD group (11.1%). | PROMS (VAS, CS, ASES, UCLA):   - RCR + SLAP = RCR + TD   Sh AROM:   - RCR + SLAP = RCR + TD   MRI (RC retear rate):   - RCR + SLAP = RCR + TD |
| Patel et al. (2021) | Systematic review | V | 741 | RC tear pathology:   - RCR + adjunctive TT (n=416)   LHB and SLAP pathology:   - TD (n=325)   Mean ± SD follow-up time:   - All types (42.5±16.5 months) | PROMS (CS, ASES, Q-DASH, SF-12 PCS), patient satisfaction, post-operative complications.  *Results dichotomised by patient demographics | Significant differences between gender groups in the TT group (42.3% males vs. 57.7% females) and in the TD (57.8% males vs. 42.2% females) group (p<0.001). Patients undergoing TT were 13.6 years older than those undergoing TD (p<0.001). High self-reported patient satisfaction scores following both TT (85.6%) and TD (92.3%). Significant increase in CS (p=0.01) ASES (p=0.04), Q-DASH (p=0.007) and SF-12PCS in TD (p=0.002). Significant increase in CS in TT (p=0.01). Post-operative cramping incidence TD (0% to 9%) vs.TT (21%). Popeye deformity incidence TD (0.0% to 6.7%) vs. TT (42% to 62%). | SATISFACTION:   - TD = TT (high)   PROMS:   - TT (increased CS, ASES, Q-DASH and SF-12PCS) - TD (increased CS)   COMPLICATIONS:   - TT > TD (Popeye deformity) - TT > TD (cramping arm pain) |
| Shang et al. (2017) | Meta-analysis | III | 903 | Repairable RC tear:   - RCR + adjunctive TD (n=361) - RCR + adjunctive TT (n=542)   Mean ± SD follow-up time:   - All types = 1.2±1.54 months (0-3) | UCLA, ASES, CS, VAS, EFSI, FSSI, ROM (Sh Flex, ER, IR), Popeye deformity, Arm cramping pain, Patient satisfaction. Results were dichotomised by patient demographics (age and gender). | Significantly higher CS in patients with RCR and adjunctive TD vs RCR and adjunctive TT (p=0.025). No significant difference between RCR and TT vs RCR and TD in UCLA (p=0.592), ASES (p=0.104), VAS (p=0.306), EFSI (p=0.910), FSSI (p=0.391) and all Sh ROM (p>0.05) scores. A significant difference in the incidence and risk of Popeye deformity in favour of RCR and TD (odds ratio 2.777 (1.731-4,455) - p < 0.001) compared with RCR and TT. No significant difference between RCR and TT vs RCR and TD in arm cramping pain (p=0.119) or patient satisfaction (p=0.498) scores. | PROMS:   - RCR + TD > RCR + TT (CS) - RCR + TD = RCR + TT (UCLA, ASES, VAS, EFSI, FSSI, SH ROM)   COMPLICATIONS:   - RCR + TT > RCR + TD (Popeye deformity) - RCR + TD = RCR + TT (cramping arm pain) |
| Watson et al. (2017) | Clinical trial | III | 80 | Repairable RC tear +/- LHB tendinopathy:   - RCR (n=35) - RCR + adjunctive TD (n=17) - RCR + adjunctive TT (n=28)   1-year follow-up data  Mean ± SD follow-up time:   - 48.4±1.7 weeks (45.3-57.0). | ASES, VAS, WORC. | All RC repair patients showed significant improvement in all three PROMS measures. Patients who had either RCR + adjunctive TD or TT demonstrated significantly greater mean improvement in PROM at 1-year follow-up compared with patients who had isolated RCR:   - ASES (mean, 42.7 vs 23.8; p= 0.002) - VAS (mean, 49.2 vs 35.7; p=0.20) - WORC scores (mean, 928 vs 743; p=0.029) - ASES scores at one year were significantly better in RCR + adjunctive TD or TT (91.6 vs 82.5; p=0.023) vs RCR group.   Linear regression found RCR + adjunctive TD or TT to be predictive of a significantly greater improvement in ASES score (p=0.01). Variance analysis revealed that the RCR with adjunctive TT (p = 0.04) and TD (p= 0.01) groups demonstrated more favourable improvement in ASES when compared with RCR alone. | PROMS:   - RCR + TD or RCR + TT > RCR in isolation (ASES, VAS, WORC) |
| Zhang et al. (2015) | Clinical trial | I | 151 | RC tears and LHB lesions:   - ARCR + adjunctive TD (n=74) - ARCR + adjunctive TT (n=77)   Mean follow-up time:   - All types = 25 months (20–29) | CS, Surgical time, Surgical cost, VAS, Popeye deformity, Cramping arm pain, Patient satisfaction level, EFSI, FSSI. | No significant differences in post-operative improvements between TT vs. TD CS (95.6% vs 96.5%), FSSI (0.9 vs 0.9), EFSI (0.9 vs 0.9) and patient satisfaction levels (65 vs 60). No significant difference in pots-operative incidence between TT vs TD in Popeye (n=7 vs n=2) sign and cramping pain (n=9 vs n= 5). Significant lower surgical times in TT vs TD (40.4 vs. 50.4 min; p<0.001). Significant lower VAS scores in TT vs TD at two weeks (3.1 vs. 4.8; p<0.001). | SATISFACTION:   - RCR + TD = RCR + TT (CS)   PROMS:   - RCR + TD = RCR + TT (CS, FFSI, EFSI) - RCR + TT < RCR + TD (Pain VAS @ 2/52)   COMPLICATIONS:   - RCR + TD = RCR + TT (Popeye deformity) - RCR + TD = RCR + TT (cramping arm pain)   SURGICAL TIMES   - RCR + TT < RCR + TD |

*List of Abbreviations: Active Range of Motion (AROM); Arthroscopic Rotator Cuff Repair (ARCR); American Shoulder and Elbow Surgeons (ASES); Constant Score (CS); Elbow Flexion Strength Index (EFSI); External Rotation (ER); Forearm Supination Strength Index (FSSI); Flexion (Flex); Internal Rotation (IR); Level of Evidence (LOE); Long Head of Biceps (LHB); Magnetic Resonance Imaging (MRI); P-value (p); Patient Reported Outcome Measures (PROMS); Quick - Disabilities of the Arm, Shoulder and Hand (Q-DASH); Rotator Cuff (RC); Rotator Cuff Repair (RCR); Range of Motion (ROM); Short Form 12 Item Health Survey - Physical Component Summary (SF-12 PCS); Shoulder (Sh); Standard Deviation (SD); Supraspinatus (SSP); Superior Labrum Anterior to Posterior (SLAP); Tenodesis (TD); Tenotomy (TT); University of California at Los Angeles (UCLA); Visual Analog Scale (VAS); Western Ontario Rotator Cuff index (WORC).*

References

1. Godeneche A, Kempf JF, Nove-Josserand L, Michelet A, Saffarini M, Hannink G, et al. Tenodesis renders better results than tenotomy in repairs of isolated supraspinatus tears with pathologic biceps. J Shoulder Elbow Surg. 2018;27(11):1939-45.

2. Gurnani N, van Deurzen DF, Janmaat VT, van den Bekerom MP. Tenotomy or tenodesis for pathology of the long head of the biceps brachii: a systematic review and meta-analysis. Knee Surg Sports Traumatol Arthrosc. 2016;24(12):3765-71.

3. Lim S, Kim SK, Kim YS. Comparison between SLAP Repair and Biceps Tenodesis with Concomitant Rotator Cuff Repair in Patients Older than 45 Years: Minimum 2-Year Clinical and Imaging Outcomes. Clin Orthop Surg. 2020;12(3):364-70.

4. Patel BH, Agarwalla A, Lu Y, Ouillette RJ, Forsythe B, Amin NH, et al. Isolated Biceps Tenodesis and Tenotomy: A Systematic Review of Indications and Patient Satisfaction. Orthopedics. 2021;44(6):333-40.

5. Shang X, Chen J, Chen S. A meta-analysis comparing tenotomy and tenodesis for treating rotator cuff tears combined with long head of the biceps tendon lesions. PLoS One. 2017;12(10):e0185788.

6. Watson ST, Robbins CB, Bedi A, Carpenter JE, Gagnier JJ, Miller BS. Comparison of Outcomes 1 Year After Rotator Cuff Repair With and Without Concomitant Biceps Surgery. Arthroscopy. 2017;33(11):1928-36.

7. Zhang Q, Zhou J, Ge H, Cheng B. Tenotomy or tenodesis for long head biceps lesions in shoulders with reparable rotator cuff tears: a prospective randomised trial. Knee Surg Sports Traumatol Arthrosc. 2015;23(2):464-9.
